# Iodination of carbohydrate-derived 1,2-oxazines to enantiopure 5-iodo-3,6-dihydro-2*H*-1,2-oxazines and subsequent palladium-catalyzed cross-coupling reactions

**DOI:** 10.3762/bjoc.12.289

**Published:** 2016-12-29

**Authors:** Michal Medvecký, Igor Linder, Luise Schefzig, Hans-Ulrich Reissig, Reinhold Zimmer

**Affiliations:** 1 Freie Universität Berlin, Institut für Chemie und Biochemie, Takustrasse 3, D-14195 Berlin, Germany

**Keywords:** amino alcohol, click reaction, cross-coupling reactions, hydrogenation, iodination, 1,2-oxazines

## Abstract

Iodination of carbohydrate-derived 3,6-dihydro-2*H*-1,2-oxazines of type **3** using iodine and pyridine in DMF furnished 5-iodo-substituted 1,2-oxazine derivatives **4** with high efficacy. The alkenyl iodide moiety of 1,2-oxazine derivatives *syn*-**4** and *anti*-**4** was subsequently exploited for the introduction of new functionalities at the C-5 position by applying palladium-catalyzed carbon–carbon bond-forming reactions such as Sonogashira, Heck, or Suzuki coupling reactions as well as a cyanation reaction. These cross-coupling reactions led to a series of 5-alkynyl-, 5-alkenyl-, 5-aryl- and 5-cyano-substituted 1,2-oxazine derivatives being of considerable interest for further synthetic elaborations. This was exemplarily demonstrated by the hydrogenation of *syn*-**21** and *anti*-**24** and by a click reaction of a 5-alkynyl-substituted precursor.

## Introduction

**Scheme 1:**
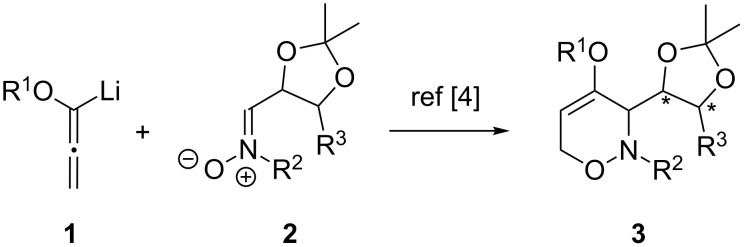
Access to enantiopure 3,6-dihydro-1,2-oxazines **3** via lithiated alkoxyallenes **1** and carbohydrate-derived nitrones **2**.

Over the last decade, we have intensively studied syntheses and applications of 3,6-dihydro-2*H*-1,2-oxazines of type **3** [1–2]. These N,O-heterocycles are easily prepared in enantiopure form by a stereodivergent [3 + 3] cyclization of carbohydrate-derived nitrones **2** and lithiated alkoxyallenes **1** (Scheme 1) [3–4]. Subsequently we investigated modifications and synthetic applications of 1,2-oxazines **3** including the preparation of seven-membered N,O-heterocycles by ring enlargement [5], functionalization of the enol ether unit [6–11], and N,O-cleavage reactions leading to amino alcohols [8,10,12], pyrroles [13] or α,β-unsaturated β-alkoxy-γ-amino-aldehydes and ketones [14]. In this context, a series of publications of our group reported on syntheses of carbohydrate mimetics [15–20] that are based on aminopyrans, aminooxepanes or other aminopolyols and that were examined for example as ligands of L- and P-selectin [21–22]. We previously reported the synthesis of enantiopure 1,2-oxazin-4-yl nonaflates and phosphates starting from precursors of type **3** and their conversion into differently C-4-substituted products employing palladium-catalyzed cross-coupling reactions [23]. In a related study, we have also investigated the synthesis of 4-halogen- and 4,5-bis(halogen)-substituted 6*H*-1,2-oxazines by halogenation of 6*H*-1,2-oxazines and subsequent palladium-catalyzed coupling reactions such as Sonogashira or Suzuki–Miyaura reactions [24] leading to aryl- and alkynyl-functionalized products. The synthetic potential of the mono- and bisalkynyl-substituted 6*H*-1,2-oxazines was additionally demonstrated by Lewis-acid-mediated conversion into highly substituted pyridine derivatives [25] by cycloaddition of in situ generated azapyrylium intermediates [26] and alkynes. Inspired by these previously reported results, we focused our interest on the so far unknown functionalization at the C-5 position of the synthetically useful enantiopure 3,6-dihydro-1,2-oxazines **3**. Herein, we now disclose our results on the iodination of **3** to provide the previously undescribed 5-iodo-1,2-oxazine derivatives **4**. These new intermediates allow subsequent C–C functionalization at the C-5 position employing various palladium-catalyzed cross-coupling reactions thus expanding the library of available enantiopure 3,6-dihydro-2*H*-1,2-oxazines.

## Results and Discussion

**Scheme 2:**
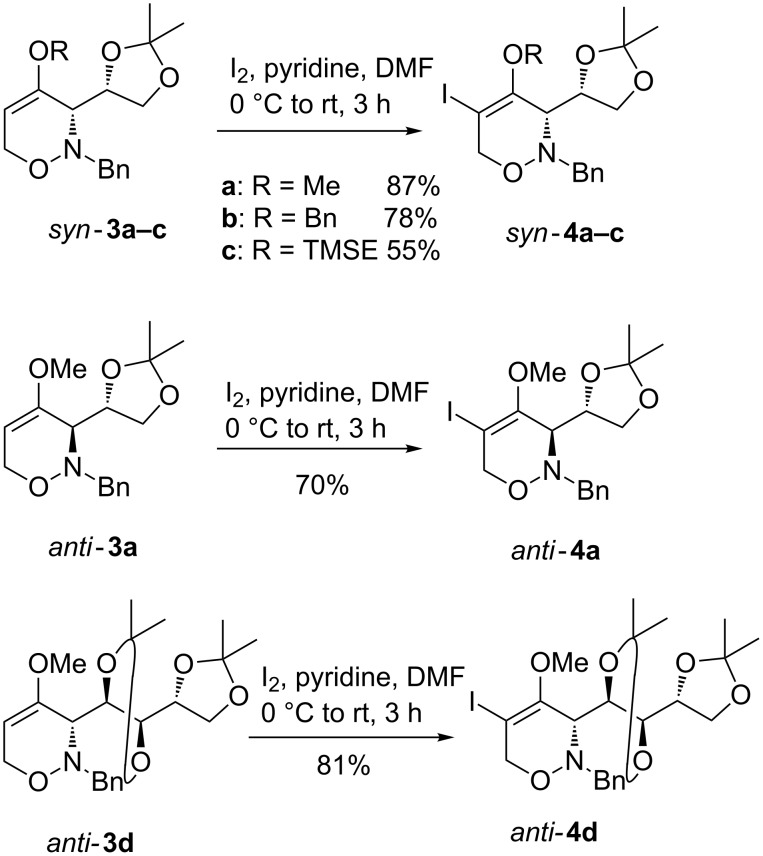
Iodination of 1,2-oxazines *syn*-**3a–c** and *anti*-**3a**,**d** leading to 5-iodo-substituted 1,2-oxazines *syn*-**4a–c** and *anti*-**4a**,**d** [TMSE = (2-trimethylsilyl)ethyl].

Numerous procedures exist for the synthesis of β-iodo enol ethers [27], although the direct β-iodination of enol ethers using a suitable electrophilic iodine reagent is relatively underdeveloped. For the iodination of 4-alkoxy-3,6-dihydro-1,2-oxazines **3**, we selected molecular iodine as the most simple iodination reagent in the presence of a base [28–30]. A clean reaction occurred upon treatment of D-glyceraldehyde-derived *syn*-configured 3,6-dihydro-1,2-oxazines *syn*-**3a–c** with three equivalents of iodine and one equivalent of pyridine as base in DMF at room temperature giving the desired 5-iodo-substituted 1,2-oxazines *syn*-**4a–c** in 55–87% yield after purification by column chromatography (Scheme 2). This operationally simple iodination protocol was also successfully applied to the *anti*-configured 1,2-oxazine *anti*-**3a** and the D-arabinose-derived starting material *anti*-**3d** furnishing the expected iodinated products *anti*-**4a** and *anti*-**4d** in good yields.

**Scheme 3:**
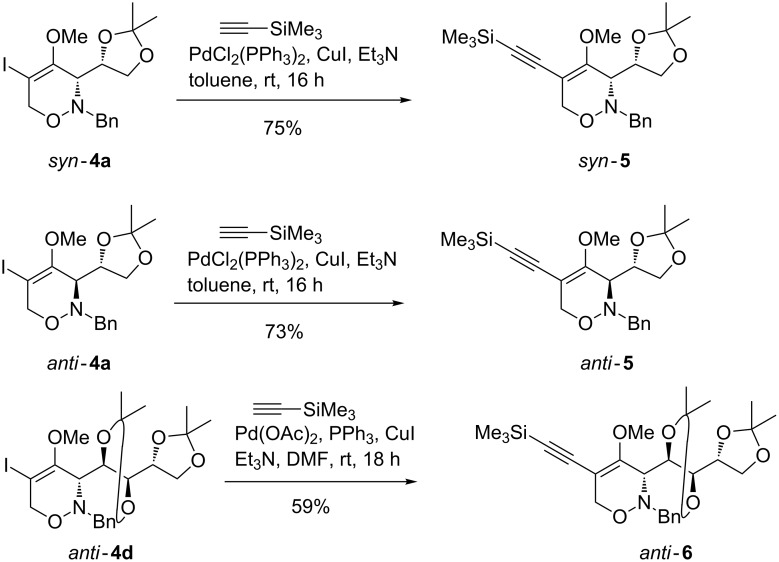
Sonogashira reactions of 4-methoxy-1,2-oxazines *syn*-**4a**, *anti*-**4a** and *anti*-**4d** leading to 5-alkynyl-substituted 1,2-oxazines *syn*-**5**, *anti*-**5** and *anti*-**6**.

Having attained an access to 5-iodo-substituted 1,2-oxazines **4**, we turned our attention to their conversion into subsequent products by taking advantage of the alkenyl iodide functionality for various palladium-catalyzed cross-coupling reactions. As a first approach to form a new C–C bond at C-5 we envisioned the Sonogashira coupling. For this purpose, we selected (trimethylsilyl)acetylene as alkyne component. The Sonogashira couplings of 5-iodo-1,2-oxazines *syn*-**4a** and *anti*-**4a** were carried out under standard conditions using a catalytic system consisting of PdCl_2_(PPh_3_)_2_, CuI and triethylamine in toluene at room temperature to furnish the corresponding 5-(trimethylsilyl)ethynyl-substituted 1,2-oxazines *syn*-**5** and *anti*-**5** in good yields (Scheme 3). When these reaction conditions were applied to the D-arabinose-derived 5-iodo-1,2-oxazine *anti*-**4d**, the desired coupling product *anti*-**6** was formed merely in 28% yield. Gratifyingly, this transformation was considerably improved when an alternative coupling protocol (Pd(OAc)_2_, PPh_3_, CuI in NEt_3_/DMF) was employed, the yield was significantly enhanced and the coupling product *anti*-**6** was obtained in 59% yield.

**Scheme 4:**
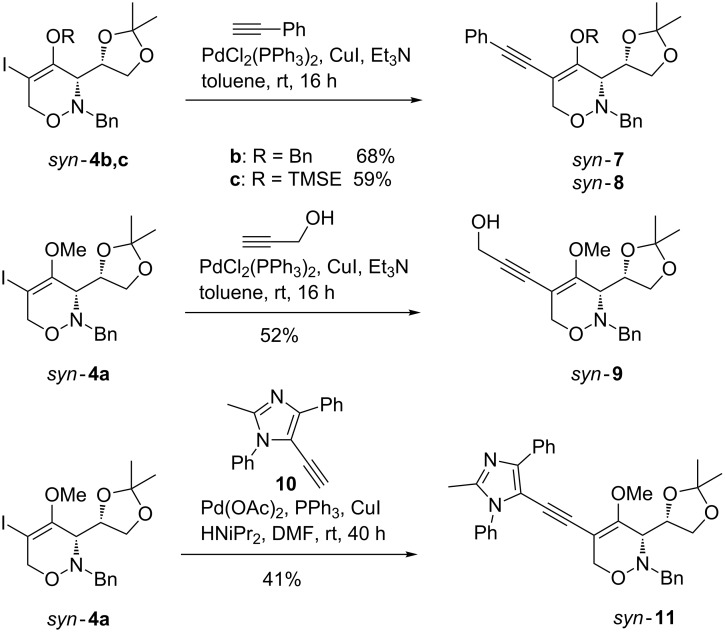
Sonogashira reactions of D-glyceraldehyde-derived 1,2-oxazines *syn*-**4a**–**c** leading to 5-alkynyl-substituted 1,2-oxazines *syn*-**7**, *syn*-**8**, *syn*-**9** and *syn*-**11**.

Sonogashira couplings of 4-benzyloxy- and 4-(2-trimethylsilyl)ethoxy-substituted 1,2-oxazines *syn*-**4b** and *syn*-**4c** with phenylacetylene worked equally well and provided under the standard conditions the desired phenylethynyl-substituted products *syn*-**7** and *syn*-**8**, respectively, in comparable yields (Scheme 4). Even the unprotected propargyl alcohol could be applied under these coupling conditions affording the corresponding 5-alkynyl-substituted 1,2-oxazine *syn*-**9**, albeit in moderate yield (52%). In the last example of Scheme 4, *syn*-**4a** was coupled with an imidazolyl-substituted alkyne **10**, that also derives from lithiated methoxyallene [31] and that was already successfully applied as alkyne component in other Sonogashira couplings presented in former publications [23,32]. The reaction of *syn*-**4a** and alkyne **10** using palladium acetate, triphenylphosphine, and copper(I) iodide in a solvent mixture of diisopropylamine and DMF at room temperature gave the 5-(imidazolylethynyl)-substituted 1,2-oxazine *syn*-**11** in 41% yield.

**Scheme 5:**
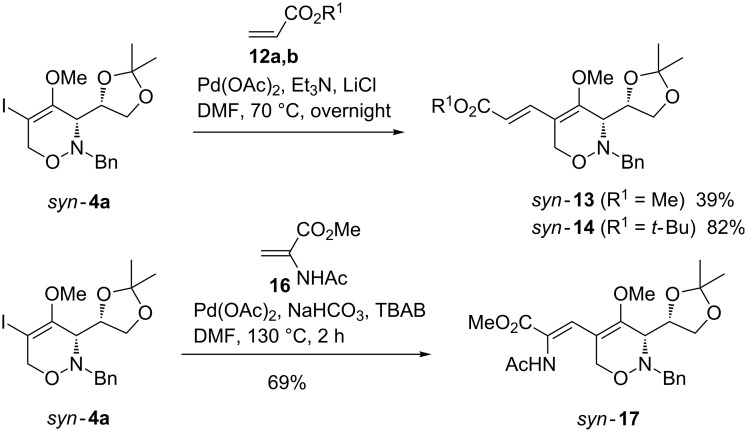
Heck reactions of 1,2-oxazine *syn*-**4a** leading to 5-alkenyl-substituted 1,2-oxazines *syn*-**13**, *syn*-**14** and to dehydroamino acid *syn*-**17**.

Next, we briefly studied 5-iodo-1,2-oxazine *syn*-**4a** as substrate in Heck reactions. The substrate was reacted with the alkyl acrylates **12a** (R^1^ = Me) and **12b** (R^1^ = *t-*Bu) under phosphine-free conditions [33–34] using 6 mol % of palladium(II) acetate, triethylamine as base and lithium chloride [35] leading to the expected coupling products *syn*-**13** and *syn*-**14** in 39% and 82% yield, respectively (Scheme 5). In both cases, only the *E*-configured 2-substituted alkyl acrylates were isolated. The moderate yield in the Heck reaction with methyl acrylate **12a** is very likely caused by the tendency of this olefin to polymerize under the conditions applied. Consequently, the change of the olefin component from the methyl to the *tert*-butyl ester allowed the preparation of the corresponding coupling product *syn*-**14** in significantly better yield. The use of dehydroamino acids such as olefin **16** [36] in Heck reactions [37–42] is also of interest, since these coupling products are useful intermediates for the synthesis of non-proteinogenic amino acids [43]. To our delight, the Heck coupling of *syn*-**4a** and **16** could be efficiently achieved employing Jeffery´s conditions [44]. When the coupling was performed using palladium(II) acetate, solid NaHCO_3_ and tetra-*n*-butylammonium bromide (TBAB) at 130 °C [37–38,42] the desired β-1,2-oxazinyl-substituted dehydroamino acid derivative *syn*-**17** was obtained in 69% yield. We did not prove the *Z*-configuration of the external C=C bond, since it is well documented that in related cross-coupling reactions of **16** exclusively the *Z*-isomers are formed [40].

**Scheme 6:**
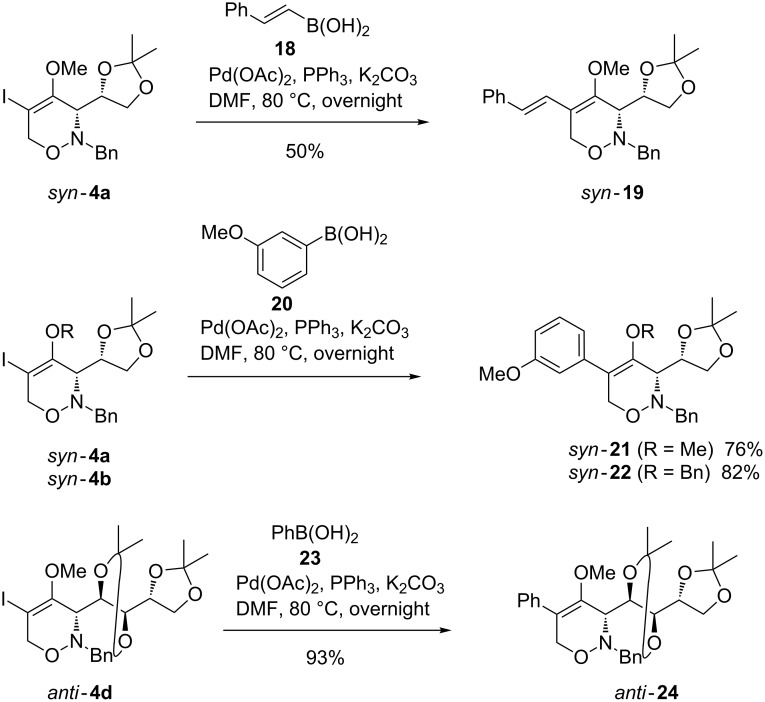
Suzuki–Miyaura reactions of 1,2-oxazines *syn*-**4a**, *syn*-**4b** and *anti*-**4d** leading to 5-styryl-substituted 1,2-oxazine *syn*-**19** and 5-aryl-substituted 1,2-oxazines *syn*-**21**, *syn*-**22** and *anti*-**24**.

The 5-iodo-1,2-oxazines *syn*-**4a,b** reacted smoothly under standard conditions of Suzuki–Miyaura coupling reactions [45] with vinylboronic acid **18** and 3-methoxyphenylboronic acid **20** to furnish the expected 5-styryl-substituted derivative *syn*-**19** and the 5-aryl-substituted derivatives *syn*-**21** and *syn*-**22**, respectively, in moderate to good yield (Scheme 6). Notably, the D-arabinose-derived 5-iodo-1,2-oxazine *anti*-**4d** was also efficiently converted into the 5-phenyl-substituted compound *anti*-**24** employing phenylboronic acid **23** under the same reaction conditions in excellent yield.

**Scheme 7:**
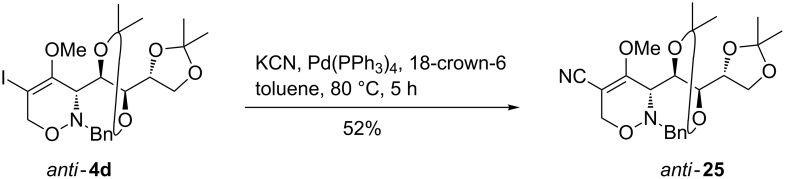
Cross-coupling reaction of 1,2-oxazine *anti*-**4d** leading to 5-cyano-substituted 1,2-oxazine *anti*-**25**.

In the last example of palladium-catalyzed reactions, we set out to prove a 5-iodo-1,2-oxazine **4** as substrate in a cyanation reaction that would lead to a 5-cyano-substituted derivative. The installation of a cyano group at the 5-position would lead to a push–pull system that could open new synthetic opportunities including the attack of nucleophiles at C-4. Metal cyanides such as KCN represent synthetically valuable C-1 building blocks that could efficiently be coupled by palladium catalysis to alkenyl triflates [46–47] or alkenyl halides [48–49] forming α,β-unsaturated nitriles. As shown in Scheme 7, we adopted a protocol described by Yamamura and Murahashi [48] and found that the palladium-catalyzed coupling of *anti*-**4d** with potassium cyanide in the presence of 18-crown-6 at 80 °C in toluene afforded the desired 5-cyano-substituted product *anti*-**25** in moderate yield (not optimized).

**Scheme 8:**
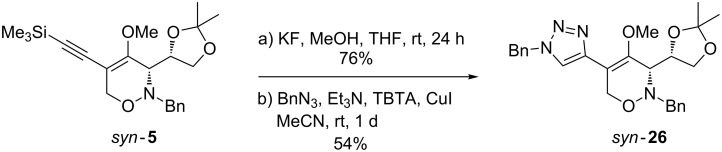
Desilylation of 1,2-oxazine *syn*-**5** and subsequent click reaction with benzyl azide leading to 5-(1,2,3-triazolyl)-substituted 1,2-oxazine *syn*-**26**.

The synthetic usefulness of the obtained 5-alkynyl-, 5-alkenyl- and 5-aryl-substituted 1,2-oxazine derivatives depends on their ability to undergo subsequent transformations. Due to their high degree of functionalization, the C-5-substituted 1,2-oxazine derivatives prepared by the coupling reactions described above should be versatile precursors for a variety of subsequent reactions, e.g., acid-catalyzed hydrolysis, hydrogenations or cyclization reactions. For example, we envisioned that 3,6-dihydro-2*H*-1,2-oxazines bearing the newly installed alkynyl group at C-5 are ideal candidates for efficient subsequent transformations. A very popular and widely applied reaction of terminal alkynes is the copper-catalyzed azide–alkyne cycloaddition, also termed as click reaction, efficiently leading to 1,4-disubstituted 1,2,3-triazoles [50]. After the desilylation of *syn*-**5** using potassium fluoride in methanol (Scheme 8) the resulting mono-substituted alkyne was subjected to an established protocol using benzyl azide, copper(I) iodide, triethylamine and TBTA [51] (for a recent review see [52]). The cycloaddition proceeded well and afforded the expected 5-(1,2,3-triazolyl)-substituted 1,2-oxazine *syn*-**26** in moderate yield (54%).

**Scheme 9:**
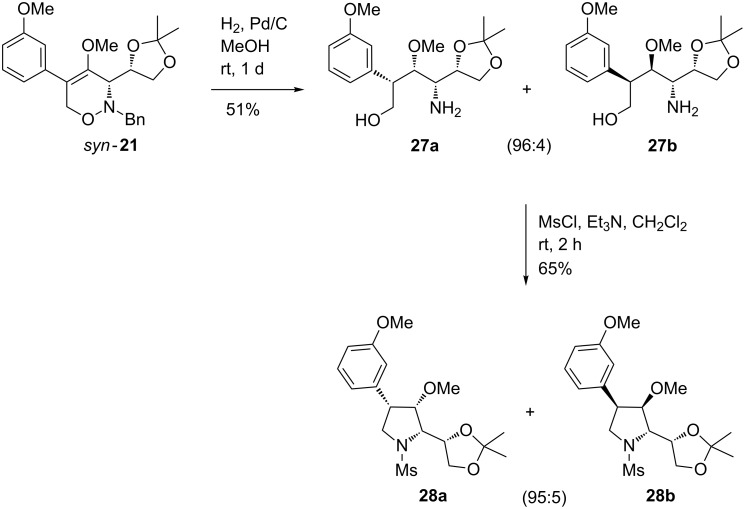
Hydrogenation of 1,2-oxazine *syn*-**21** leading to γ-amino alcohols **27a**,**b** and subsequent ring closure to pyrrolidine derivatives **28a**,**b**.

Hydrogenolysis belongs to the well-established transformations of 1,2-oxazines, often successfully leading to valuable compounds including 1,4-amino alcohols or pyrrolidine derivatives. We therefore briefly examined the reaction of 5-aryl-substituted 1,2-oxazines *syn*-**21** and *anti*-**24** under previously established conditions [53]. The hydrogenolysis of 1,2-oxazine derivative *syn*-**21** in methanol using palladium on charcoal as catalyst afforded the expected α-(3-methoxyphenyl)-substituted γ-amino alcohol derivative **27** in 51% yield as 96:4 mixture of two diastereomers (Scheme 9). Subsequent ring closure of γ-amino alcohol **27** by treatment with mesyl chloride in the presence of triethylamine [53] furnished the *N*-mesylated pyrrolidine derivative **28** in 65% yield with excellent diastereoselectivity (dr 95:5).

**Scheme 10:**
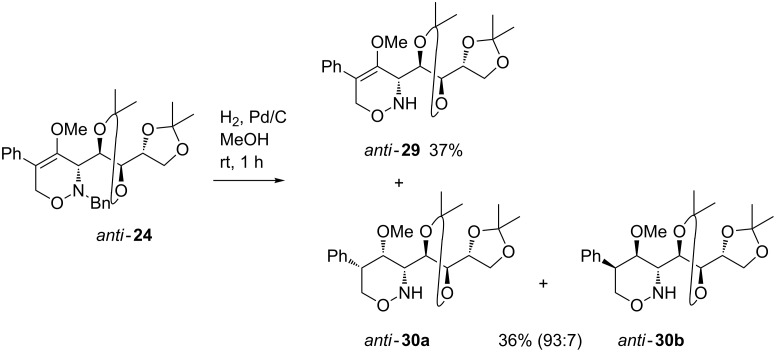
Hydrogenation of 1,2-oxazine *anti*-**24** to products *anti*-**29** and *anti*-**30**.

In contrast, when these hydrogenolysis conditions were applied to the D-arabinose-derived 5-phenyl-1,2-oxazine *anti*-**24** merely an inseparable complex mixture of products was obtained. After shortening of the reaction time from one day to one hour, we were able to isolate two products, the *N*-debenzylated 3,6-dihydro-2*H*-1,2-oxazine *anti*-**29** in 37% yield and the 3,4,5,6-tetrahydro-2*H*-1,2-oxazine *anti*-**30** in 36% yield as a 93:7 mixture of diastereomers (Scheme 10). The dependence of product distribution, especially in the latter case, revealed that a specific reaction sequence is operating during the hydrogenolysis processes. As already discussed in previous publications [23,53], the hydrogenolysis of 1,2-oxazines of type **3** with palladium on charcoal very likely starts with a fast *N*-debenzylation, followed by the reduction of the C-4/C-5 double bond forming the corresponding 3,4,5,6-tetrahydro-2*H*-1,2-oxazines that after cleavage of the N–O bond provide the corresponding amino alcohols. In the second reaction step, the hydrogen attacks the C=C bond mainly from the less hindered side (here *trans* to the fairly bulky 3-dioxolanyl group) leading to the preferred configuration of *anti*-**30a** as depicted in Scheme 10.

The three successful transformations demonstrate the potential of 1,2-oxazines with substituents at C-5 for further elaborations, they also show that careful optimizations are required in each individual case, in particular for the hydrogenolysis reactions.

## Conclusion

We have demonstrated that the enol ether unit of 3,6-dihydro-2*H*-1,2-oxazines **3** can efficiently be converted into the corresponding 5-iodo-substituted compounds **4** under mild reaction conditions using molecular iodine in the presence of pyridine as base. The obtained alkenyl iodides **4** are ideal candidates for further transformations. As shown in this report, the subsequent cross-coupling reactions at C-5 position considerably broaden the scope of available 3,6-dihydro-2*H*-1,2-oxazines that are highly functionalized and have high potential for further synthetic elaborations, in particular for the preparation of enantiopure acyclic and cyclic amino alcohols.

## Supporting Information

File 1 General information, all experimental procedures and analytical data.

File 2 Copies of ^1^H and ^13^C NMR spectra of compounds **4**–**9**, **11**, **14**, **19**, **21**, **24**–**27, 29** and **30**.
