# Supplementary material for: Iodination of carbohydrate-derived 1,2-oxazines to enantiopure 5-iodo-3,6-dihydro-2H-1,2-oxazines and subsequent palladium-catalyzed cross-coupling reactions
Source: Beilstein J Org Chem. 2016 Dec 29;12:2898–905. doi: 10.3762/bjoc.12.289 (PMC5238527; doi:10.3762/bjoc.12.289)
Supplement: File 1 — General information, all experimental procedures and analytical data. [file Beilstein_J_Org_Chem-12-2898-s001.pdf]

Supporting Information

for

Iodination of carbohydrate-derived 1,2-oxazines to enantiopure 5-iodo-3,6-dihydro-2*H*-1,2-oxazines and subsequent palladium-catalyzed cross-coupling reactions

Michal Medvecký[1], Igor Linder[1], Luise Schefzig[1], Hans-Ulrich Reissig*[1] and Reinhold Zimmer*[1]

Address: [1]Freie Universität Berlin, Institut für Chemie und Biochemie, Takustrasse 3, D-14195 Berlin, Germany

Email: Hans-Ulrich Reissig - hans.reissig@chemie.fu-berlin.de, Reinhold Zimmer - rzimmer@zedat.fu-berlin.de

* Corresponding author

General information, all experimental procedures and analytical data

Contents

1) General information S2
2) Experimental procedures S2

General information

Reactions were generally performed under argon in flame-dried flasks. Solvents and reagents were added by syringes. Solvents were dried using standard procedures. Triethylamine was distilled from calcium hydride and stored under argon over KOH. *N,N*-Dimethylformamide was purchased in p. A. purity grade and stored under argon over activated 4 Å molecular sieves. Dichloromethane was purified with the MB SPS-800-dry solvent system. Hexanes were distilled from CaH_2, ethyl acetate was distilled from K_2CO_3 and $CaCl_2$. Products were purified by flash chromatography on silica gel (230–400 mesh, Merck).

^{1}H NMR [$CHCl_3$ (δ = 7.26 ppm) or TMS (δ = 0.00 ppm) as internal standard] and ^{13}C NMR spectra [$CDCl_3$ (δ = 77.16 ppm) as internal standard] were recorded on Bruker (AC 500, AVIII 700) and JEOL (ECX 400, Eclipse 500) instruments in $CDCl_3$ solution. Integrals are in accordance with assignments; coupling constants are given in Hz. For detailed peak assignments 2D spectra were measured (COSY, HMQC, HMBC). IR spectra were measured with an FT-IR spectrometer Nicolet 5 SXC or with a Nexus FT-IR equipped with a Nicolet Smart DuraSamplIR ATR. MS and HRMS analyses were performed with Varian Ionspec QFT-7 (ESI-FT ICRMS), Agilent 6210 (ESI–TOF) or Finnigan MAT 711 (EI, 80 eV, 8 kV) instruments. Elemental analyses were carried out with CHN-Analyzer Vario EL or Vario EL III instruments. Optical rotations ($[\alpha]_D$) were determined with Perkin-Elmer 141 or Perkin-Elmer 241 polarimeter at the temperatures given. Melting points were measured with a Reichert apparatus (Thermovar) and are uncorrected.

General procedure 1 (GP1): Iodination of 1,2-oxazine derivatives **3**

To a solution of 1,2-oxazine **3** (1 equiv) in DMF (4 mL/mmol **3**) were successively added iodine (4 equiv) and pyridine (1 equiv) at 0 °C under argon atmosphere. The mixture was stirred for 3 h at room temperature and subsequently quenched with sat. aq $NaHCO_3$ solution (10 mL/mmol **3**). After extraction with CH_2Cl_2 (3 × 15 mL/mmol **3**) the combined organic phases were washed with sat. aq $Na_2S_2O_3$ solution and H_2O (1 × 15 mL each/mmol **3**) and subsequently dried ($MgSO_4$). After removal of the solvent under reduced pressure the crude product was purified by flash chromatography (hexanes/EtOAc).

(3*S*,4'*S*)-2-Benzyl-3-(2',2'-dimethyl-1',3'-dioxolan-4'-yl)-5-iodo-4-methoxy-3,6-dihydro-2*H*-1,2-oxazine (*syn*-4a)

According to GP1, the reaction of *syn*-**3a** (1.86 g, 6.10 mmol), iodine (6.22 g, 24.5 mmol) and pyridine (0.490 g, 6.10 mmol) in DMF (25 mL) gave after flash chromatography (silica gel, hexanes/EtOAc, 4:1 to 2:1) *syn*-**4a** (2.30 g, 87%) as colorless oil.

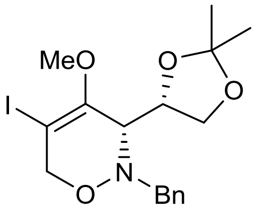

$[\alpha]_D^{22}$ 76.8 (*c* 0.35, $CHCl_3$).

IR (neat): ν = 3105-2835 (=C-H, C-H), 1660 cm^{-1} (C=C).

^{1}H NMR ($CDCl_3$, 400 MHz): δ = 1.34, 1.37 (2 s, 3 H each, Me), 3.62 (s, 3 H, OMe), 3.71 (br dd, *J* ≈ 1.5, 6.5 Hz, 1 H, 3-H), 3.84 (dd, *J* = 7.7, 8.8 Hz, 1 H, 5'-H), 4.00 (dd, *J* = 5.9, 8.8 Hz, 1 H, 5'-H), 4.10-4.18 (m, 3 H, 6-H, NCH_2), 4.39 (dd, *J* = 1.8, 15.0 Hz, 1 H, 6-H), 4.49-4.57 (m, 1 H, 4'-H), 7.23-7.44 (m, 5 H, Ph).

^{13}C NMR ($CDCl_3$, 125.8 MHz): δ = 26.0, 26.6 (2 q, Me), 58.3 (t, NCH_2), 58.5 (q, OMe), 63.7 (d, C-3), 66.4 (t, C-5'), 67.9 (t, C-6), 74.8 (d, C-4'), 79.9 (s, C-5), 108.6 (s, C-2'), 127.1, 128.2, 128.5, 137.4 (3 d, s, Ph), 155.5 (s, C-4).

Anal. calcd. for $C_{17}H_{22}INO_4$ (431.3): C 47.35, H 5.14, N 3.25; found: C 47.55, H 4.62, N 3.21.

(3*S*,4'*S*)-2-Benzyl-4-benzyloxy-3-(2',2'-dimethyl-1',3'-dioxolan-4'-yl)-5-iodo-3,6-dihydro-2*H*-1,2-oxazine (*syn*-4b)

According to GP1, the reaction of *syn*-**3b** (0.500 g, 1.31 mmol), iodine (1.33 g, 5.24 mmol) and pyridine (0.104 g, 1.31 mmol) in DMF (5 mL) gave after flash chromatography (silica gel, hexanes/EtOAc, 6:1) *syn*-**4b** (0.516 g, 78%) as colorless solid, mp 109 °C.

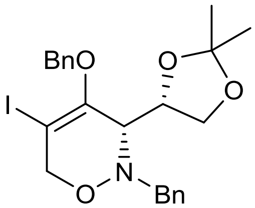

$[\alpha]_D^{22}$ 127.5 (*c* 1.26, $CHCl_3$).

IR (KBr): ν = 3090-2835 (=C-H, C-H), 1655 cm^{-1} (C=C).

^{1}H NMR ($CDCl_3$, 500 MHz): δ = 1.34, 1.39 (2 s, 3 H each, Me), 3.71 (br dd, *J* ≈ 1.6, 6.0 Hz, 1 H, 3-H), 3.91 (dd, *J* = 7.7, 8.8 Hz, 1 H, 5'-H), 3.98 (d, *J* = 13.9 Hz, 1 H, NCH_2Ph), 4.03 (dd, *J* = 6.0, 8.8 Hz, 1 H, 5'-H), 4.06 (d, *J* = 13.9 Hz, 1 H, NCH_2Ph), 4.17 (d, *J* = 15.0 Hz, 1 H, 6-H), 4.43 (dd, *J* = 1.6, 15.0 Hz, 1 H, 6-H), 4.55 (td, *J* = 6.0, 7.7 Hz, 1 H, 4'-H), 4.87, 4.94 (2 d, *J* = 11.6 Hz, 1 H each, OCH_2Ph), 7.25-7.43 (m, 10 H, Ph).

^{13}C NMR ($CDCl_3$, 125.8 MHz): δ = 26.0, 26.6 (2 q, Me), 58.1 (t, NCH_2), 63.5 (d, C-3), 66.7 (t, C-5'), 71.9 (t, OCH_2), 72.2 (t, C-6), 75.3 (d, C-4'), 78.5 (s, C-5), 108.7 (s, C-2'), 127.4, 128.2, 128.3, 128.4, 128.6, 128.7, 136.2, 137.0 (5 d, 2 s, Ph), 150.7 (s, C-4).

HRMS (ESI-ToF): calcd for $C_{23}H_{27}INO_4$ $[M+H]^+$: 508.0985; found 508.0986.

Anal. calcd. for $C_{23}H_{26}INO_4$ (507.4): C 54.45, H 5.17, N 2.76; found: C 54.15, H 5.16, N 2.76.

(3*S*,4'*S*)-2-Benzyl-3-(2',2'-dimethyl-1',3'-dioxolan-4'-yl)-5-iodo-4-(trimethylsilyl-ethoxy)-3,6-dihydro-2*H*-1,2-oxazine (*syn*-4c)

According to GP1, the reaction of *syn*-**3c** (0.391 g, 1.00 mmol), iodine (1.02 g, 4.00 mmol) and pyridine (0.079 g, 1.00 mmol) in DMF (4 mL) gave after flash chromatography (silica gel, hexanes/EtOAc, 4:1 to 1:1) *syn*-**4c** (0.284 g, 55%) as yellow oil.

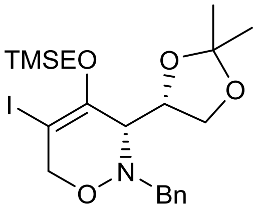

$[\alpha]_D^{25}$ 102.1 (*c* 0.73, $CHCl_3$).

IR (neat): ν = 3085-2840 (=C-H, C-H), 1655 cm^{-1} (C=C).

^{1}H NMR ($CDCl_3$, 250 MHz): δ = 0.01 (s, 9 H, $SiMe_3$), 1.00-1.12 (m, 2 H, CH_2Si), 1.33, 1.37 (2 s, 3 H each, Me), 3.61 (br d, *J* = 6.3 Hz, 1 H, 3-H), 3.72-4.06 (m, 5 H, 5'-H, 6-H, OCH_2), 4.12 (s, 2 H, NCH_2), 4.43 (dd, *J* = 1.5, 14.9 Hz, 1 H, 6-H), 4.49 (dt, *J* = 6.3, 6.9 Hz, 1 H, 4'-H), 7.20-7.43 (m, 5 H, Ph).

^{13}C NMR ($CDCl_3$, 100.6 MHz): δ = -1.34 (q, $SiMe_3$), 18.6 (t, $SiCH_2$), 26.0, 26.7 (2 q, Me), 58.3 (t, NCH_2), 63.7 (d, C-3), 66.6 (t, C-5'), 68.1 (t, C-6), 72.6 (t, OCH_2), 74.8 (d,

C-4'), 77.9 (s, C-5), 108.7 (s, C-2'), 127.3, 128.3, 128.6, 137.2 (3 d, s, Ph), 151.1 (s, C-4).

HRMS (ESI-ToF): calcd for $C_{21}H_{33}INO_4Si$ [M+H]$^+$: 518.1218; found 518.1233.

(3*R*,4'*S*)-2-Benzyl-3-(2',2'-dimethyl-1',3'-dioxolan-4'-yl)-5-iodo-4-methoxy-3,6-dihydro-2*H*-1,2-oxazine (*anti*-4a)

According to GP1, the reaction of *anti*-**3a** (0.386 g, 1.26 mmol), iodine (1.28 g, 5.04 mmol) and pyridine (0.100 g, 1.26 mmol) in DMF (5 mL) gave after flash chromatography (silica gel, hexanes/EtOAc, 6:1) *anti*-**4a** (0.380 g, 70%) as yellow oil.

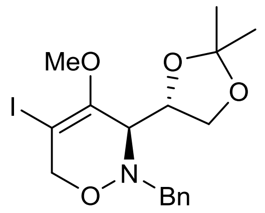

$[\alpha]_D^{22}$ 87.1 (*c* 0.85, $CHCl_3$).

IR (neat): ν = 3085-2835 (=C-H, C-H), 1660 cm^{-1} (C=C).

^{1}H NMR ($CDCl_3$, 400 MHz): δ = 1.34, 1.38 (2 s, 6 H, Me), 3.46 (d, *J* = 6.9 Hz, 1 H, 3-H), 3.68 (s, 3 H, OMe), 3.90 (dd, *J* = 6.4, 8.6 Hz, 1 H, 5'-H), 4.07-4.14 (m, 3 H, 5'-H, NCH_2), 4.28 (d, *J* = 15.3 Hz, 1 H, NCH_2), 4.40-4.55 (m, 2 H, 5'-H, 6-H), 7.27-7.42 (m, 5 H, Ph).

^{13}C NMR ($CDCl_3$, 125.8 MHz): δ = 25.3, 26.6 (2 q, Me), 58.0 (q, OMe), 58.1 (t, NCH_2), 63.0 (d, C-3), 67.9 (t, C-5'), 70.2 (t, C-6), 73.4 (s, C-5), 77.4 (d, C-4'), 109.9 (s, C-2'), 128.1, 128.9, 129.1, 136.6 (3 d, s, Ph), 152.7 (s, C-4).

HRMS (ESI-ToF): calcd for $C_{17}H_{22}INO_4Na$ [M+Na]$^+$: 454.0486; found 454.0493.

(3*S*,4'*S*,4''*R*,5''*R*)-2-Benzyl-5-iodo-4-methoxy-3-(2',2',2'',2''-tetramethyl-4',4''-bi-1,3-dioxolan-5'-yl)-3,6-dihydro-2*H*-1,2-oxazine (*anti*-4d)

According to GP1, the reaction of *anti*-**3d** (0.466 g, 1.15 mmol), iodine (1.17 g, 4.60 mmol) and pyridine (0.091 g, 1.15 mmol) in DMF (5 mL) gave after flash chromatography (silica gel, hexanes/EtOAc, 6:1) *anti*-**4d** (0.495 g, 81%) as yellow oil.

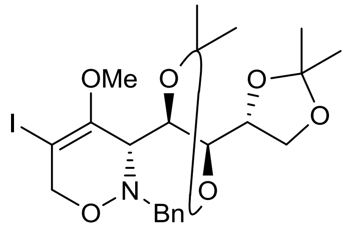

anti-**4d**

$[\alpha]_D^{22}$ 73.7 (*c* 1.45, $CHCl_3$).

IR (neat): ν = 3090-2835 (=C-H, C-H), 1660 cm^{-1} (C=C).

^{1}H NMR ($CDCl_3$, 400 MHz): δ = 1.335, 1.34, 1.36, 1.38 (4 s, 12 H, Me), 3.67 (s, 3H, OMe), 3.91 (dd, *J* = 6.7, 8.1 Hz, 1 H, 5''-H), 4.03-4.29 (m, 8 H, NCH_2, 3-H, 4'-H, 4''-H, 5'-H, 5''-H, 6-H), 4.41 (dd, *J* = 1.9, 15.3 Hz, 1 H, 6-H), 7.26-7.41 (m, 5 H, Ph).

^{13}C NMR ($CDCl_3$, 101.8 MHz): δ = 25.6, 26.5, 27.1, 27.3 (4 q, 4 Me), 58.0 (t, NCH_2), 58.2 (q, OMe), 62.9 (d, C-3), 66.3 (t, C-6), 70.5 (t, C-5'), 75.6 (d, C-4'), 78.5 (d, C-4''), 80.0 (d, C-5'), 89.7 (s, C-5), 109.5, 109.9 (2 s, C-2', C-2''), 127.8, 128.6, 128.9, 136.6 (3 d, s, Ph), 152.2 (s, C-4).

HRMS (ESI-ToF): calcd for $C_{22}H_{30}INO_6$ Na $[M+Na]^+$: 554.1016; found 554.1066.

Anal. calcd. for $C_{22}H_{30}INO_6$ (531.9): C 49.68, H 5.69, N 2.64; found: C 49.40, H 5.63, N 2.76.

General procedure 2 (GP2): Sonogashira coupling of 5-iodo-1,2-oxazines **4**

In a heat-gun-dried and argon-flushed flask, 5-iodo-1,2-oxazine **4** (1 equiv), the corresponding alkyne (1.5 equiv), $PdCl_2(PPh_3)_2$ (5–10 mol %), CuI (3–5 mol %), and Et_3N (3 equiv) were dissolved in toluene (7–9 mL/mmol **4**) and the reaction mixture was stirred at room temperature for 16 h. Then, the solvent was removed under reduced pressure and the residue was dissolved in EtOAc (10 mL/mmol **4**), washed with water (10 mL/mmol **4**) and dried (Na_2SO_4). Purification of the crude product by column chromatography (silica gel, hexanes/EtOAc) afforded the 5-alkynyl-substituted 1,2-oxazine.

(3*S*,4'*S*)-2-Benzyl-3-(2',2'-dimethyl-1,3-dioxolan-4'-yl)-4-methoxy-5-[(trimethyl-silyl)ethynyl]-3,6-dihydro-2*H*-1,2-oxazine (*syn*-5)

According to GP2, 5-iodo-1,2-oxazine *syn*-**4a** (0.431 g, 1.00 mmol), trimethylsilyl-acetylene (0.198 g, 1.50 mmol), $PdCl_2(PPh_3)_2$ (36 mg, 0.052 mmol), CuI (6 mg, 0.032 mmol), and Et_3N (0.42 mL, 3.00 mmol) were dissolved in toluene (7 mL) and the reaction mixture was stirred at room temperature. Workup and purification by column

chromatography (silica gel, hexanes/EtOAc, 6:1) according to GP2 afforded the 5-trimethylsilylethynyl-substituted 1,2-oxazine *syn*-**5** (0.300 g, 75%) as a pale yellow solid, melting range 104-110 °C.

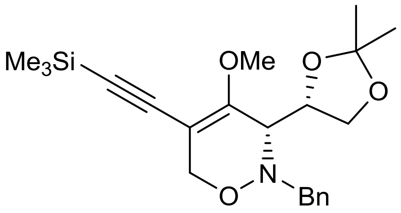

$[\alpha]_D^{22}$ 1.6 (*c* 0.63, $CHCl_3$).

IR (KBr): ν = 3100-2850 (=C-H, C-H), 2220 (C≡CH), 1600 cm^{-1} (C=C).

^{1}H NMR (500 MHz, $CDCl_3$): δ = 0.17 (s, 9 H, TMS), 1.36, 1.39 (2 s, 6 H, Me), 3.32 (br d, *J* ≈ 6.9 Hz, 1 H, 3-H), 3.52-3.95 (m, *2* H, 5'-H), 4.03 (d, *J* = 14.0 Hz, 1 H, 6-H), 4.08 (s, 3 H, OMe), 4.14 (s, 2 H, CH_2Ph), 4.32 (dd, *J* = 1.3, 14.0 Hz, 1 H, 6-H), 4.49-4.55 (m, 1 H, 4'-H), 7.16-7.30, 7.38-7.45 (2 m, 3 H, 2 H, Ph).

^{13}C NMR (125.8 MHz, $CDCl_3$) δ = -0.26 (q, TMS), 26.1, 26.6 (2 q, Me), 58.1 (t, NCH_2), 58.6 (q, OMe), 63.3 (d, C-3), 66.6 (t, C-5'), 67.4 (t, C-6), 74.7 (d, C-4'), 94.1, 99.4, 100.1 (3 s, C≡C, C-5), 108.6 (s, C-2'), 127.1, 128.2, 128.6, 137.5 (3 d, s, Ph), 155.8 (s, C-4).

Anal. calcd. for $C_{22}H_{31}NO_4Si$ (401.6): C 65.80, H 7.78, N 3.49; found: C 65.25, H 7.69, N 3.50.

(3*R*,4'*S*)-2-Benzyl-3-(2',2'-dimethyl-1,3-dioxolan-4'-yl)-4-methoxy-5-[(trimethyl-silyl)ethynyl]-3,6-dihydro-2*H*-1,2-oxazine (*anti*-5)

According to GP2, 5-iodo-1,2-oxazine *anti*-**4a** (0.340 g, 0.788 mmol), trimethyl-silylacetylene (0.17 mL, 1.19 mmol), $PdCl_2(PPh_3)_2$ (28 mg, 0.039 mmol), CuI (5 mg, 0.026 mmol), and Et_3N (0.33 mL, 2.36 mmol) were dissolved in toluene (10 mL) and the reaction mixture was stirred at room temperature. Workup and purification by column chromatography (silica gel, hexanes/EtOAc, 10:1) according to GP2 afforded the 5-alkynyl-substituted 1,2-oxazine *anti*-**5** (0.230 g, 73%) as a pale yellow oil.

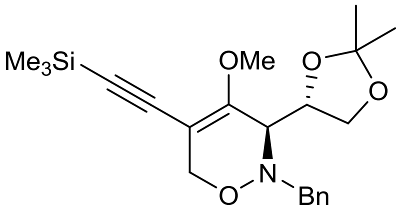

$[\alpha]_D^{22}$ 17.5 (*c* 0.6, $CHCl_3$).

IR (neat): ν = 3090-2850 (=C-H, C-H), 2200 (C≡CH), 1650 cm^{-1} (C=C).

^{1}H NMR (500 MHz, $CDCl_3$): δ = 0.18 (s, 9 H, TMS), 1.33, 1.39 (2 s, 6 H, Me), 3.25 (br d, *J* ≈ 5.7 Hz, 1 H, 3-H), 3.98-4.05 (m, 3 H, 5'-H, CH_2Ph), 4.06 (s, 3 H, OMe), 4.13-4.18 (m, 2 H, CH_2Ph, 6-H), 4.26 (dd, *J* = 1.4, 14.3 Hz, 1 H, 6-H), 4.49 (q, *J* = 6.1 Hz, 1 H, 4'-H), 7.24-7.38 (m, 5 H, Ph).
^{13}C NMR (126 MHz, $CDCl_3$): δ = -0.06 (q, TMS), 25.3, 26.5 (2 q, Me), 58.2 (t, NCH_2), 59.0 (q, OMe), 62.5 (d, C-3), 65.0 (t, C-6), 67.2 (t, C-5'), 77.0 (d, C-4'), 94.5, 99.4, 100.6 (3 s, C≡C, C-5), 109.4 (s, C-2'), 127.5, 128.5, 128.7, 137.0 (3 d, s, Ph), 156.7 (s, C-4).
HRMS (ESI-ToF): calcd for $C_{22}H_{32}NO_4Si$ $[M+H]^+$: 402.2100; found: 402.2101.

(3*S*,4'*S*,4''*R*,5'*R*)-2-Benzyl-4-methoxy-3-(2',2',2'',2''-tetramethyl-4',4''-bi-1,3-dioxolan-5'-yl)-5-[(trimethylsilyl)ethynyl]-3,6-dihydro-2*H*-1,2-oxazine (*anti*-6)
In a heat-gun-dried and argon-flushed flask, *anti*-**4d** (0.300 g, 0.565 mmol), triisopropylsilylacetylene (0.056 g, 0.570 mmol), $Pd(OAc)_2$ (6.5 mg, 0.028 mmol), CuI (11 mg, 0.057 mmol), and Et_3N (2 mL) were dissolved in DMF (10 mL) and the reaction mixture was stirred at room temperature for 18 h. Then, the solvent was removed under reduced pressure and the residue was dissolved in EtOAc (10 mL), washed with water (3 × 5 mL) and dried (Na_2SO_4). Purification of the crude product by column chromatography (silica gel, hexanes/EtOAc, 10:1) afforded the 5-alkynyl-substituted 1,2-oxazine *anti*-**6** (0.168 g, 59%) as a yellow oil.

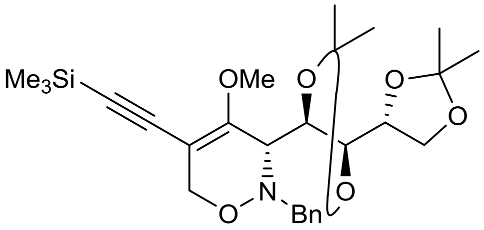

$[\alpha]_D^{22}$ 34.7 (*c* 0.15, $CHCl_3$).
IR (neat): ν = 3100-2920 (=C-H, C-H), 2210 (C≡CH), 1650 cm^{-1} (C=C).
^{1}H NMR (700 MHz, C_6D_6): δ = 0.13 (s, 9 H, TMS), 1.27, 1.29, 1.35, 1.38 (4 s, 3 H each, Me), 3.69 (br s, 1 H, 3-H), 3.98-4.04 (m, 3 H, NCH_2, 5''-H), 4.04 (s, 3 H, OMe), 4.07-4.11 (m, 2 H, NCH_2, 4''-H), 4.16-4.20 (m, 1 H, 6-H), 4.33 (dd, *J* = 1.5, 14.0 Hz, 1 H, 6-H), 4.45 (t, *J* = 7.4 Hz, 1 H, 4'-H), 4.53 (dd, *J* = 2.9, 7.7 Hz, 1 H, 5'-H), 7.05-7.08, 7.12-7.14, 7.33-7.36 (3 m, 1 H, 2 H, 2 H, Ph).
^{13}C NMR (176 MHz, C_6D_6): δ = -0.1 (q, TMS), 25.7, 26.7, 27.3, 27.4 (4 q, Me), 58.3 (t, NCH_2), 58.8 (q, OMe), 63.0 (d, C-3), 66.2 (t, C-6), 67.5 (t, C-5''), 77.8 (d, C-4''),

79.2 (d, C-4'), 80.6 (d, C-5'), 94.0 (s, C-5), 99.6, 101.2 (2 s, C≡C), 109.5, 109.7 (2 s, C-2', C-2''), 127.5, 128.5, 129.0, 137.9 (3 d, s, Ph), 157.1 (s, C-4).

HRMS (pos. ESI): calcd for $C_{27}H_{39}NO_6SiNa$ $[M+Na]^+$ 524.2439; found 524.2450.

(3*S*,4'*S*)-2-Benzyl-4-benzyloxy-3-(2',2'-dimethyl-1,3-dioxolan-4'-yl)-5-(phenylethynyl)-3,6-dihydro-2*H*-1,2-oxazine (*syn*-7)

According to GP2, 5-iodo-1,2-oxazine *syn*-**4b** (0.429 g, 0.845 mmol), phenylacetylene (0.14 mL, 1.27 mmol), $PdCl_2(PPh_3)_2$ (31 mg, 0.042 mmol), CuI (5 mg, 0.026 mmol), and Et_3N (0.35 mL, 2.53 mmol) were dissolved in toluene (6 mL) and the reaction mixture was stirred at room temperature. Workup and purification by column chromatography (silica gel, hexanes/EtOAc, 7:1 to 4:1) according to GP2 afforded the 5-phenylethynyl-substituted 1,2-oxazine *syn*-**7** (0.566 g, 68%) as a yellow oil.

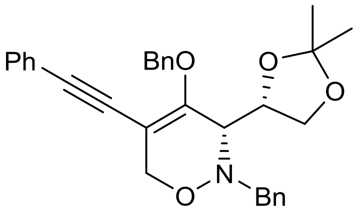

$[\alpha]_D^{23}$ 52.8 (*c* 0.80, $CHCl_3$).

IR (neat): ν = 3100-2850 (=C-H, C-H), 2220 (C≡CH), 1600 cm^{-1} (C=C).

^{1}H NMR (500 MHz, $CDCl_3$): δ = 1.34, 1.40 (2 s, 6 H, Me), 3.42 (br d, *J* ≈ 7.7 Hz, 1 H, 3-H), 3.89 (t, *J* = 8.4 Hz, 1 H, 5‘-H), 3.95 (dd, *J* = 5.8, 8.4 Hz, 1 H, 5‘-H), 4.14 (s, 3 H, OMe), 4.20 (t, *J* = 14.1 Hz, 1 H, 6-H), 4.46 (dd, *J* = 1.9, 14.1 Hz, 1 H, 6-H), 4.56-4.62 (m, 1 H, 4‘-H), 5.31, 5.63 (2 d, *J* = 12.1 Hz, 1 H each, OCH_2), 7.26-7.44 (m, 5 H, Ph).

^{13}C NMR (125.8 MHz, $CDCl_3$): δ = 26.1, 26.6 (2 q, Me), 58.2 (t, NCH_2), 63.3 (d, C-3), 66.9 (t, C-5'), 67.2 (t, C-6), 72.7 (t, OCH_2), 74.9 (d, C-4'), 83.5, 95.2, 96.3 (3 s, C≡C, C-5), 108.7 (s, C-2'), 123.2, 127.2, 127.9, 128.1, 128.2, 128.3, 128.5, 128.6, 131.0, 136.7, 137.5 (s, 8 d, 2 s, Ph), 154.1 (s, C-4).

HRMS (ESI-ToF): calcd for $C_{31}H_{31}NO_4Na$ $[M+Na]^+$ 504.2145; found: 504.2098.

(3*S*,4'*S*)-2-Benzyl-3-(2',2'-dimethyl-1,3-dioxolan-4'-yl)-5-(phenylethynyl)-4-[(trimethylsilyl)ethyloxy]-3,6-dihydro-2*H*-1,2-oxazine (*syn*-8)

According to GP2, 5-iodo-1,2-oxazine *syn*-**4c** (0.110 g, 0.213 mmol), phenylacetylene (34.5 μL, 0.32 mmol), $PdCl_2(PPh_3)_2$ (5 mg, 0.02 mmol), CuI (3 mg, 0.02 mmol), and iPr_2NH (0.5 mL) were dissolved in toluene (1 mL) and the reaction mixture was

stirred at room temperature. Workup and purification by column chromatography (silica gel, hexanes/EtOAc, 9:1) according to GP2 afforded the 5-phenylethynyl-substituted 1,2-oxazine *syn*-**8** (62 mg, 59%) as a yellow oil.

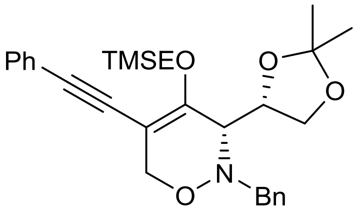

$[\alpha]_D^{20}$ 31.6 (*c* 0.98, $CHCl_3$).

IR (neat): ν = 3110-2860 (=C-H, C-H), 2220 (C≡CH), 1670 cm^{-1} (C=C).

^{1}H NMR (500 MHz, $CDCl_3$): δ = 0.02 (s, 9 H, $SiMe_3$), 1.02-1.16 (m, 2 H, CH_2Si), 1.38, 1.41 (2 s, 3 H each, Me), 3.33 (br d, *J* = 7.0 Hz, 1 H, 3-H), 3.93-4.01 (m, 2 H, 5'-H), 4.13-4.22 (m, 3 H, 6-H, NCH_2), 4.32-4.37 (m, 1 H, OCH_2), 4.43 (dd, *J* = 1.3, 13.8 Hz, 1 H, 6-H), 4.56 (td, *J* = 7.0, 7,2 Hz, 1 H, 4'-H), 4.66-4.73 (m, 1 H, OCH_2), 7.23-7.38, 7.42-7.45 (2 m, 8 H, 2 H, Ph).

^{13}C NMR (125.8 MHz, $CDCl_3$): δ = -1.45 (q, $SiMe_3$), 18.8 (t, $SiCH_2$), 26.2, 26.6 (2 q, Me), 58.3 (t, NCH_2), 63.7 (d, C-3), 66.9 (t, C-5'), 67.4 (t, C-6), 69.0 (t, OCH_2), 74.6 (d, C-4'), 84.1, 93.9, 94.4 (3 s, C≡C, C-5), 108.6 (s, C-2'), 123.4, 127.1, 128.0, 128.2, 128.3, 128.6, 131.0, 136.7 (s, 6 d, s, Ph), 154.2 (s, C-4).

Anal. calcd. for $C_{31}H_{31}NO_4$ (481.6): C 77.31, H 6.49, N 2.91; found: C 77.55, H 6.62, N 3.29.

(3*S*,4'*S*)-2-Benzyl-3-(2',2'-dimethyl-1,3-dioxolan-4'-yl)-4-methoxy-5-(3-hydroxy-prop-1-yn-1-yl)-3,6-dihydro-2*H*-1,2-oxazine (*syn*-9)

According to GP2, 5-iodo-1,2-oxazine *syn*-**4a** (0.231 g, 0.536 mmol), propargyl alcohol (44 mg, 0.631 mmol), $PdCl_2(PPh_3)_2$ (37 mg, 0.053 mmol), CuI (20 mg, 0.104 mmol), and Et_3N (0.22 mL, 1.60 mmol) were dissolved in toluene (5 mL) and the reaction mixture was stirred at room temperature. Workup and purification by column chromatography (silica gel, hexanes/EtOAc, 4:1 to 1:2) according to GP2 afforded the 5-alkynyl-substituted 1,2-oxazine *syn*-**9** (0.100 g, 52%) as a pale yellow resin.

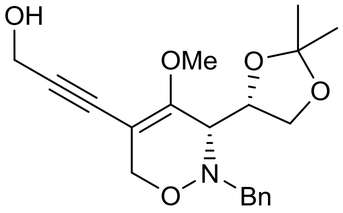

$[\alpha]_D^{23}$ 51.1 (*c* 0.42, $CHCl_3$).

IR (neat): ν = 3435 (O-H), 3110-2845 (=C-H, C-H), 2210 (C≡CH), 1650 cm^{-1} (C=C).

^1H NMR (500 MHz, $CDCl_3$): δ = 1.35, 1.38 (2 s, 6 H, Me), 2.10 (br s, 1 H, OH), 3.38 (br d, *J* ≈ 5.3 Hz, 1 H, 3-H), 3.86 (t, *J* = 6.9 Hz, 1 H, 5'-H), 3.94 (dd, *J* = 4.7, 6.9 Hz, 1 H, 5'-H), 4.00 (s, 3 H, OMe), 4.03 (d, *J* = 11.2 Hz, 1 H, 6-H), 4.11 (s, 2 H, NCH_2), 4.31 (dd, *J* = 1.0, 11.2 Hz, 1 H, 6-H), 4.37 (s. 2 H. OCH_2), 4.49-4.53 (m, 1 H, 4'-H), 7.25-7.50 (m, 5 H, Ph).

^{13}C NMR (125.8 MHz, $CDCl_3$): δ = 26.0, 26.5 (2 q, Me), 51.6 (t, OCH_2), 58.1 (t, CH_2Ph), 58.5 (q, OMe), 62.9 (d, C-3), 66.6 (t, C-5'), 67.2 (t, C-6), 74.7 (d, C-4'), 79.6, 92.9 (2 s, C≡C), 94.0 (s, C-5), 108.6 (s, C-2'), 127.2, 128.2, 128.6, 137.3 (3 d, s, Ph), 155.3 (s, C-4).

Anal. calcd. for $C_{20}H_{25}NO_5$ (359.4): C 66.83, H 7.01, N 3.90; found: C 66.01, H 6.73, N 3.67.

(3*S*,4'*S*)-2-Benzyl-3-(2',2'-dimethyl-1,3-dioxolan-4'-yl)-4-methoxy-5-(2-methyl-1,5-diphenyl-1*H*-imidazol-4-yl)-3,6-dihydro-2*H*-1,2-oxazine (*syn*-11)

In a heat-gun-dried and argon-flushed flask, 5-iodo-1,2-oxazine *syn*-**4a** (0.108 g, 0.250 mmol), alkyne **10** (80 mg, 0.300 mmol), $Pd(OAc)_2$ (4 mg, 0.018 mmol), CuI (3 mg, 0.013 mmol), PPh_3 (13 mg, 0.050 mmol), and iPr_2NH (0.4 mL) were dissolved in DMF (0.4 mL) and the reaction mixture was stirred at room temperature for 40 h. The mixture was quenched with sat. aq. $NaHCO_3$ solution (3 mL) and extracted with CH_2Cl_2 (3 x 30 mL), and dried (Na_2SO_4). Purification by column chromatography (silica gel, hexanes/EtOAc, 4:1 to 1:1) afforded the 5-alkynyl-substituted 1,2-oxazine *syn*-**11** (50 mg, 41%) as a colorless solid, mp = 56-60 °C.

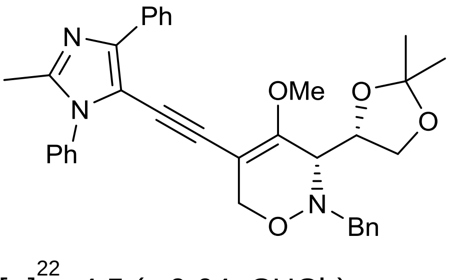

$[\alpha]_D^{22}$ -4.5 (*c* 0.64, $CHCl_3$).

IR (KBr): ν = 3160–2850 (=C-H, C-H), 2190 (C≡C), 1620 cm^{-1} (C=C).

^1H NMR (500 MHz, $CDCl_3$): δ = 1.35, 1.39 (2 s, 3 H each, Me), 2.29 (s, 3 H, Me), 3.33 (br d, *J* = 6.9 Hz, 1 H, 3-H), 3.86 (dd, *J* = 8.3, 8.5 Hz, 1 H, 5'-H), 3.92 (dd, *J* = 6.7, 8.5 Hz, 1 H, 5'-H), 3.94 (s, 3 H, OMe), 4.12 (d, *J* = 13.7 Hz, 1 H, 6-H), 4.13 (s, 2

H, NCH_2), 4.39 (dd, J = 1.1, 13.7 Hz, 1 H, 6-H), 4.50-4.55 (m, 1 H, 4'-H), 7.10-7.15, 7.21-7.34, 7.38-7.43 (3 m, 2 H, 4 H, 9 H, Ph).

^{13}C NMR (125.8 MHz, $CDCl_3$): δ = 14.1, 26.1, 26.5 (3 q, Me), 58.1 (t, CH_2Ph), 58.6 (q, OMe), 63.4 (d, C-3), 66.7 (t, C-5'), 67.2 (t, C-6), 74.9 (d, C-4'), 83.9, 88.7 (2 s, C≡C), 93.7 (s, C-5), 108.6 (s, C-2'), 121.4, 127.1, 127.7, 135.8, 136.5, 137.2, 146.1 (7 s, C-4, Ph, imidazole), 127.6, 128.0, 128.2, 128.6, 128.7, 128.8, 128.9, 129.5 (8 d, Ph).

HRMS (ESI-ToF): calcd for $C_{35}H_{36}N_3O_4$ $[M+H]^+$ 562.2706; found: 562.2708.

General procedure 3 (GP3): Heck reaction of 5-iodo-1,2-oxazines **4**

A reaction flask containing 5-iodo-1,2-oxazine **4** (1 equiv) in DMF (2.5–4 mL/mmol **4**) was degassed and filled with argon. LiCl (1.6 equiv) and Et_3N (3 equiv) were added, followed by the corresponding acrylate (1.5 equiv). After stirring at 70 °C overnight, the mixture was taken up in EtOAc and H_2O (20 mL/mmol **4**). The aqueous layer was extracted with EtOAc (3 × 20 mL/mmol **4**). The combined organic extracts were washed brine (20 mL/mmol **4**), dried (Na_2SO_4), filtered and concentrated to dryness. The residue was purified by column chromatography (silica gel, hexanes/EtOAc) to afford the corresponding coupling product.

(*E*)-Methyl 3-[(3*S*,4'*S*)-2-Benzyl-3-(2',2'-dimethyl-1,3-dioxolan-4'-yl)-4-methoxy-3,6-dihydro-2*H*-1,2-oxazin-5-yl]acrylate (*syn*-13)

According to GP3, the reaction of *syn*-**4a** (0.218 g, 0.503 mmol), methyl acrylate **12a** (68 μL, 0.755 mmol), $Pd(OAc)_2$ (7 mg, 0.03 mmol), LiCl (34.2 mg, 0.806 mmol), and NEt_3 (0.21 mL, 1.51 mmol), in DMF (2 mL) gave after column chromatography (silica gel, hexanes/EtOAc, 4:1) *syn*-**13** (75 mg, 39%) as a yellow oil.

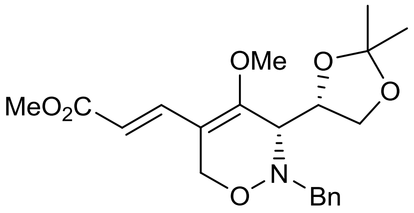

$[\alpha]_D^{22}$ 88.5 (*c* 0.65, $CHCl_3$).

IR (neat): ν = 3080-2940 (=C-H, C-H), 1700 (C=O), 1640 cm^{-1} (C=C).

^{1}H NMR (500 MHz, $CDCl_3$): δ = 1.37, 1.39 (2 s, 3 H each, Me), 3.71, 3.75 (2 s, 3 H each, OMe), 3.80 (br d, J = 5.1 Hz, 1 H, 3-H), 3.83 (dd, J = 7.7, 9.0 Hz, 1 H, 5'-H), 4.04 (dd, J = 6.4, 9.0 Hz, 1 H, 5'-H), 4.09, 4.12 (AB system, J_{AB} = 12.9 Hz, 2 H,

CH_2Ph), 4.24 (d, *J* = 14.1 Hz, 1 H, 6-H), 4.42 (br d, *J* = 14.3 Hz, 1 H, 6-H), 4.52-4.62 (m, 1 H, 4'-H), 5.53 (d, *J* = 16.3 Hz, 1 H, =CH), 7.25-7.42 (m, 5 H, Ph), 7.83 (d, *J* = 16.1 Hz, 1 H, =CH).

^{13}C NMR (125.8 MHz, $CDCl_3$): δ = 25.9, 26.4 (2 q, Me), 51.7 (q, CO_2*Me*), 57.9 (q, OMe), 58.2 (t, CH_2Ph), 59.5 (d, C-3), 64.4 (t, C-6), 66.5 (t, C-5'), 75.1 (d, C-4'), 108.6 (s, C-2'), 113.7 (s, C-5), 114.4 (d, =CH), 127.4, 128.4, 128.6 (3 d, Ph), 135.8 (d, =CH), 137.0 (s, Ph), 154.7 (s, C-4), 167.5 (s, *C*O_2Me).

Anal. calcd. for $C_{21}H_{27}NO_6$ (389.4): C 64.77, H 6.99, N 3.60; found: C 65.25, H 6.52, N 3.31.

(*E*)-*tert*-Butyl 3-[(3*S*,4'*S*)-2-Benzyl-3-(2',2'-dimethyl-1,3-dioxolan-4'-yl)-4-methoxy-3,6-dihydro-2*H*-1,2-oxazin-5-yl]acrylate (*syn*-14)

According to GP3, the reaction of *syn*-**4a** (0.510 g, 1.18 mmol), *tert*-butyl acrylate **12b** (0.26 mL, 1.77 mmol), $Pd(OAc)_2$ (16 mg, 0.071 mmol), LiCl (80 mg, 1.89 mmol), and NEt_3 (0.49 mL, 3.55 mmol), in DMF (5 mL) gave after column chromatography (silica gel, hexanes/EtOAc, 4:1) *syn*-**14** (0.420 g, 82%) as an orange oil.

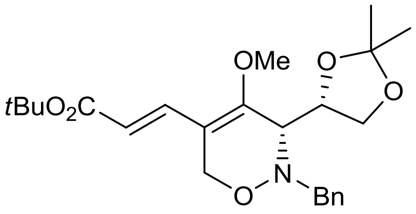

$[\alpha]_D^{22}$ 142.9 (*c* 1.44, $CHCl_3$).

IR (neat): ν = 3070-2840 (=C-H, C-H), 1700 (C=O), 1630, 1610 cm^{-1} (C=C).

^{1}H NMR (500 MHz, $CDCl_3$): δ = 1.36, 1.38 (2 s, 3 H each, Me), 1.49 (s, 9 H, *t*-Bu), 3.69 (s, 3 H, OMe), 3.77 (dd, *J* = 1.2, 5.5 Hz, 1 H, 3-H), 3.84 (dd, *J* = 7.7, 8.9 Hz, 1 H, 5'-H), 4.03 (dd, *J* = 5.7, 8.9 Hz, 1 H, 5'-H), 4.09, 4.11 (d, *J* = 13.8 Hz, 1 H each, 6-H), 4.24 (d, *J* = 14.3 Hz, 1 H, CH_2Ph), 4.49 (dd, *J* = 7.4, 14.3 Hz, 1 H, CH_2Ph), 4.56 (dt, *J* = 5.7, 7.7 Hz, 1 H, 4'-H), 5.50 (dd, *J* = 7.4, 16.4 Hz, 1 H, =CH), 7.26-7.42 (m, 5 H, Ph), 7.77 (d, *J* = 16.4 Hz, 1 H, =CH).

^{13}C NMR (125 MHz, $CDCl_3$): δ = 25.9, 26.4 (2 q, Me), 28.2 (q, *t*-Bu), 58.0 (t, CH_2Ph), 58.2 (q, OMe), 59.5 (d, C-3), 64.4 (t, C-6), 66.5 (t, C-5'), 75.2 (d, C-4'), 80.3 (s, *t*-Bu), 99.9 (s, C-5), 108.6 (s, C-2'), 116.9 (d, =CH), 127.4, 128.4, 128.6 (3 d, Ph), 134.6 (d, =CH), 137.1 (s, Ph), 154.0 (s, C-4), 166.6 (s, CO_2*t*-Bu).

HRMS (ESI-ToF): calcd for $C_{24}H_{33}NO_6$ $[M+H]^+$ 432.2386; found: 432.2394.

(Z)-Methyl 2-acetamido-3-[(3S,4'S)-2-benzyl-3-(2',2'-dimethyl-1,3-dioxolan-4'-yl)-4-methoxy-3,6-dihydro-2H-1,2-oxazin-5-yl]acrylate (*syn*-17)

To a solution of *syn*-**4a** (0.226 g, 0.524 mmol) and acrylate **16** (0.120 g, 0.838 mmol) in dry DMF (3 mL) were added $Pd(OAc)_2$ (14 mg, 0.06 mmol), TBAB (0.320 g, 0.996 mmol), and $NaHCO_3$ (0.132 g, 1.57 mmol) and the resulting mixture was heated under argon atmosphere in an ACE-sealed tube at 130 °C for 2 h. After cooling to room temperature the mixture was diluted with EtOAc (10 mL), quenched with H_2O (10 mL) and the aqueous layer was extracted with EtOAc (3 × 10 mL). The combined organic extracts were dried (Na_2SO_4) and the solvent was evaporated under reduced pressure. The residue was purified by column chromatography (silica gel, hexanes/EtOAc, 1:1) to give *syn*-**17** (0.162 g, 69%) as a pale yellow foam.

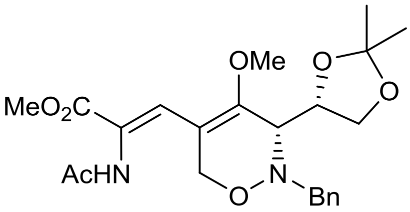

$[\alpha]_D^{20}$ 124.8 (*c* 0.25, $CHCl_3$).

IR (neat): ν = 3200 (N-H), 3085-2850 (=C-H, C-H), 1720, 1695 (C=O), 1670, 1615 cm^{-1} (C=C).

^1H NMR (500 MHz, $CDCl_3$): δ = 1.38, 1.40, 2.09 (3 s, 3 H each, Me), 3.69, 3.80 (2 s, 3 H each, OMe), 3.75 (br d, J = 5.1 Hz, 1 H, 3-H), 3.83-4.09 (m, 2 H, 5'-H), 4.11 (s, 2 H, CH_2Ph), 4.23 (d, J = 14.8 Hz, 1 H, 6-H), 4.40 (br d, J = 15.0 Hz, 1 H, 6-H), 4.51-4.59 (m, 1 H, 4'-H), 6.97 (br s, 1 H, =CH), 7.25-7.40 (m, 5 H, Ph).

^{13}C NMR (125.8 MHz, $CDCl_3$): δ = 23.2, 25.5, 26.4 (3 q, Me), 52.5 (q, CO_2*Me*), 58.2 (q, OMe), 58.4 (t, CH_2Ph), 60.1 (d, C-3), 65.0 (t, C-6), 66.4 (t, C-5'), 75.3 (d, C-4'), 108.6 (s, C-2'), 112.5 (s, C-5), 123.6 (d, =CH), 125.0, 127.4, 128.4, 128.6, 137.0 (s, 3 d, s, Ph, =*C*NHAc), 151.5 (s, C-4), 165.6, 169.8 (2 s, *C*O_2Me, NHAc).

HRMS (ESI-ToF): calcd for $C_{23}H_{30}N_2O_7$ $[M+H]^+$ 447.2131; found: 447.2119.

Anal. calcd. for $C_{23}H_{30}N_2O_7$ (446.5): C 61.87, H 6.77, N 6.27; found: C 61.34, H 6.72, N 6.16.

General procedure 4 (GP4): Suzuki coupling of 5-iodo-1,2-oxazines **4**:

A reaction flask containing 5-iodo-1,2-oxazine **4** (1 equiv), PPh_3 (20 mol %), and $Pd(OAc)_2$ (5 mol %), was degased and filled with argon. DMF (5–10 mL/mmol **4**) and K_2CO_3 (1 equiv) were added, followed by the corresponding boronic acid (1.2 equiv).

After stirring at 70 °C overnight, the mixture was diluted with H_2O (10 mL/mmol **4**) and extracted with EtOAc (3 × 15 mL/mmol **4**). The combined organic extracts were washed with H_2O (10 mL/mmol **4**) and brine (10 mL/mmol **4**), dried (Na_2SO_4), filtered and concentrated to dryness. The residue was purified by column chromatography (silica gel, hexanes/EtOAc) to afford the corresponding coupling product.

(3*S*,4'*S*)-2-Benzyl-3-(2',2'-dimethyl-1',3'-dioxolan-4'-yl)-4-methoxy-5-(2-phenylvinyl)-3,6-dihydro-2*H*-1,2-oxazine (*syn*-19)

According to GP4, the reaction of *syn*-**4a** (0.150 g, 0.417 mmol), 2-phenylvinylboronic acid **18** (74 mg, 0.500 mmol), K_2CO_3 (84 mg, 0.625 mmol), PPh_3 (22 mg, 0.084 mmol), and $Pd(OAc)_2$ (5 mg, 0.022 mmol) in DMF (2 mL) gave after column chromatography (silica gel, hexanes/EtOAc, 4:1) *syn*-**19** (85 mg, 50%) as an orange oil.

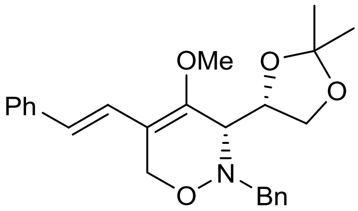

$[\alpha]_D^{22}$ -33.2 (*c* 0.33, $CHCl_3$).

IR (neat): ν = 3105-2840 (=C-H, C-H), 1650, 1600 cm^{-1} (C=C).

^1H NMR (500 MHz, $CDCl_3$): δ = 1.40, 1.42 (2 s, 3 H each, Me), 3.68 (s, 3 H, OMe), 3.70 (br d, *J* = 6.1 Hz,1 H, 3-H), 3.94 (dd, *J* = 7.8, 8.7 Hz, 1 H, 5'-H), 4.08 (dd, *J* = 6.0, 8.7 Hz, 1 H, 5'-H), 4.19 (s, 2 H, CH_2Ph), 4.43 (d, *J* = 14.2 Hz, 1 H, 6-H), 4.59-4.62 (m, 1 H, 4'-H), 4.65 (br d, *J* = 14.0 Hz, 1 H, 6-H), 6.23 (d, *J* = 16.8 Hz, 1 H, =CH), 7.20-7.38, 7.42-7.48 (2 m, 7 H, 4 H, =CH, Ph).

^{13}C NMR (125 MHz, $CDCl_3$): δ = 25.9, 26.5 (2 q, Me), 58.1 (q, OMe), 58.3 (t, CH_2Ph), 59.58 (d, C-3), 63.5 (t, C-6), 66.6 (t, C-5'), 75.6 (d, C-4'), 108.5 (s, C-2'), 116.5 (s, C-5), 120.0, 125.9 (2 d, HC=CH), 126.2, 127.2, 127.4, 128.3, 128.55, 128.6, 137.37, 137.48 (6 d, 2 s, Ph), 148.8 (s, C-4).

HRMS (ESI-ToF): calcd for $C_{25}H_{30}NO_4$ $[M+H^+]$: 408.2175; found: 408.2163.

Anal. calcd. for $C_{25}H_{29}NO_4$ (407.5): C 73.69, H 7.17, N 3.44; found: C 73.16, H 7.02, N 3.36.

(3*S*,4'*S*)-2-Benzyl-3-(2',2'-dimethyl-1',3'-dioxolan-4'-yl)-4-methoxy-5-(3-methoxyphenyl)-3,6-dihydro-2*H*-1,2-oxazine (*syn*-21)

According to GP4, the reaction of *syn*-**4a** (0.427 g, 0.990 mmol), 3-methoxyphenylboronic acid **20** (0.181 g, 1.19 mmol), K_2CO_3 (0.205 g, 1.49 mmol), PPh_3 (52 mg, 0.199 mmol), and $Pd(OAc)_2$ (12 mg, 0.053 mmol) in DMF (5 mL) gave after column chromatography (silica gel, hexanes/EtOAc, 6:1) *syn*-**21** (0.309 g, 76%) as yellow oil.

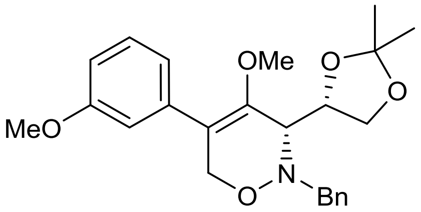

$[\alpha]_D^{22}$ 84.0 (*c* 0.84, $CHCl_3$).

IR (neat): ν = 3090-2830 (=C-H, C-H), 1670 cm^{-1} (C=C).

^{1}H NMR (500 MHz, $CDCl_3$): δ = 1.39, 1.42 (2 s, 3 H each, Me), 3.42 (s, 3 H, OMe), 3.57 (m_c, 1 H, 3-H), 3.81 (s, 3 H, OMe), 3.97 (t, *J* = 8.6 Hz, 1 H, 5'-H), 4.05 (dd, *J* = 5.9, 8.6 Hz, 1 H, 5'-H), 4.19, 4.21 (2 d, *J* = 14.0 Hz, 1 H each, 6-H), 4.29 (d, *J* = 14.8 Hz, 1 H, CH_2Ph), 4.57 (dd, *J* = 1.6, 14.8 Hz, 1 H, CH_2Ph), 4.63 (ddd, *J* = 5.9, 6.7, 8.1 Hz, 1 H, 4'-H), 6.81-6.83, 6.93-6.96, 7.25-7.29, 7.32-7.35, 7.45-7.46 (5 m, 10 H, Ph).

^{13}C NMR (125 MHz, $CDCl_3$): δ = 26.1, 26.6 (2 q, Me), 55.2 (q, OMe), 58.3 (t, C-6), 58.4 (q, OMe), 61.5 (d, C-3), 66.8 (t, C-5'), 67.7 (t, CH_2Ph), 75.3 (d, C-4'), 108.5 (s, C-2'), 112.6, 114.1 (2 d, Ph), 116.7 (s, C-5), 120.6, 127.2, 128.3, 128.6, 129.3, 136.5, 137.7 (5 d, 2 s, Ph), 147.4 (s, C-4), 159.4 (s, Ph).

Anal. calcd. for $C_{24}H_{29}NO_5$ (411.5): C 70.05, H 7.10, N 3.40; found: C 69.78, H 7.12, N 3.59.

(3*S*,4'*S*)-2-Benzyl-4-benzyloxy-3-(2',2'-dimethyl-1',3'-dioxolan-4'-yl)-5-(3-methoxyphenyl)-3,6-dihydro-2*H*-1,2-oxazine (*syn*-22)

According to GP4, the reaction of *syn*-**4b** (0.290 g, 0.572 mmol), 3-methoxyphenylboronic acid **20** (0.104 g, 0.686 mmol), K_2CO_3 (0.118 g, 0.857 mmol), PPh_3 (30 mg, 0.115 mmol), and $Pd(OAc)_2$ (7 mg, 0.031 mmol) in DMF (5 mL) gave after column chromatography (silica gel, hexanes/EtOAc, 6:1) *syn*-**22** (241 mg, 82%) as pale yellow oil.

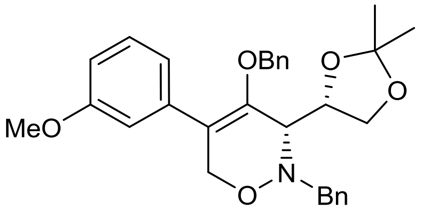

$[\alpha]_D^{22}$ 13.2 (*c* 0.65, $CHCl_3$).

IR (neat): ν = 3090-2840 (=C-H, C-H), 1660 cm^{-1} (C=C).

^{1}H NMR (500 MHz, $CDCl_3$): δ = 1.37, 1.41 (2 s, 3 H each, Me), 3.60 (dd, *J* = 1.5, 6.6 Hz 1 H, 3-H-), 3.73 (s, 3 H, OMe), 4.01 (t, *J* = 8.7 Hz, 1 H, 5'-H), 4.07 (dd, *J* = 5.8, 8.7 Hz, 1 H, 5'-H), 4.14, 4.16 (2 d, *J* = 14.0 Hz, 1 H each, 6-H), 4.26 (d, *J* = 14.9 Hz, 1 H, NCH_2Ph), 4.53 (d, *J* = 11.3 Hz, 1 H, OCH_2Ph), 4.64 (ddd, *J* = 5.8, 6.6, 8.1 Hz, 1 H, 4'-H), 4.67 (dd, *J* = 1.5, 14.9 Hz, 1 H, NCH_2Ph), 4.71 (d, *J* = 11.3 Hz, 1 H, OCH_2Ph), 6.80-7.43 (m, 14 H, Ph).

^{13}C NMR (125 MHz, $CDCl_3$): δ = 26.1, 26.6 (2 q, Me), 55.1 (q, OMe), 58.4 (t, C-6), 61.8 (d, C-3), 67.1 (t, C-5'), 67.5 (t, NCH_2Ph), 72.4 (t, OCH_2Ph), 75.6 (d, C-4'), 108.6 (s, C-2'), 113.0, 113.7 (2 d, Ph), 118.6 (s, C-5), 120.4, 127.1, 127.9, 128.3, 128.3, 128.7, 129.3, 136.5, 136.8, 137.7 (7 d, 3 s, Ph), 146.3 (s, C-4), 159.4 (s, Ph).

HRMS (pos. ESI): calcd for $C_{30}H_{33}NO_5$ [M^+]: 487.2359; found: 487.2367.

(3*S*,4'*S*,4''*R*,5'*R*)-2-Benzyl-4-methoxy-5-phenyl-3-(2',2',2'',2''-tetramethyl-4',4''-bi-1,3-dioxolan-5'-yl)-3,6-dihydro-2*H*-1,2-oxazine (*anti*-24)

According to GP4, the reaction of *syn*-**4d** (0.190 g, 0.358 mmol), phenylboronic acid **23** (52 mg, 0.426 mmol), K_2CO_3 (49 mg, 0.358 mmol), PPh_3 (19 mg, 0.072 mmol), and $Pd(OAc)_2$ (4 mg, 0.018 mmol) in DMF (3.5 mL) gave after column chromatography (silica gel, hexanes/EtOAc, 6:1) *anti*-**24** (160 mg, 93%) as a colorless oil.

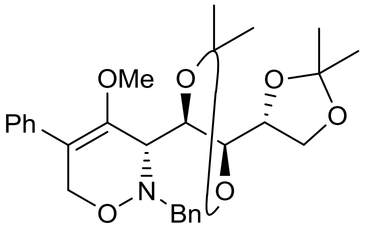

$[\alpha]_D^{25}$ 12.8 (*c* 0.21, $CHCl_3$).

IR (neat): ν = 3090-2835 (=C-H, C-H), 1670 cm^{-1} (C=C).

^{1}H NMR (500 MHz, $CDCl_3$): δ = 1.42, 1.41, 1.37, 1.34 (4 s, 12 H, Me), 3.39 (s, 3 H, OMe), 3.65 (m_c, 1 H, 3-H), 3.99 (dd, *J* = 6.7, 8.1 Hz, 1 H, 5''-H), 4.08-4.15 (m, 2 H,

5''-H, CH_2Ph), 4.23-4.29 (m, 2 H, NCH_2, 4'-H), 4.35 (dd, J = 3.5, 7.6 Hz, 1 H, 5'-H), 4.38-4.44 (m, 2 H, 4''-H, 6-H), 4.53 (dd, J = 1.9, 15.1 Hz, 1 H, 6-H), 7.24-7.29, 7.31-7.37, 7.40-7.45 (3 m, 2 H, 4 H, 4 H, Ph).

^{13}C NMR (125 MHz, $CDCl_3$): δ = 25.6, 26.6, 27.3, 27.3 (4 q, Me), 58.4 (t, NCH_2), 59.0 (q, OMe), 61.6 (d, C-3), 65.8 (t, C-6), 66.4 (t, C-5''), 77.1 (d, C-4'), 78.3 (d, C-4''), 80.0 (d, C-5'), 109.45, 109.50 (2 s, C-2', C-2''), 116.7 (s, C-5), 127.5, 128.3, 128.45, 128.5, 128.8, 135.2, 137.3 (5 d, 2 s, Ph), 147.7 (s, C-4).

HRMS (pos. ESI): calcd for $C_{28}H_{35}NO_6$ $[M+H]^+$: 482.2537; found: 482.2556.

(3*S*,4'*S*,4''*R*,5''*R*)-2-Benzyl-5-cyano-4-methoxy-3-(2',2',2'',2''-tetramethyl-4',4''-bi-1,3-dioxolan-5'-yl)-3,6-dihydro-2*H*-1,2-oxazine (*anti*-25)

To a solution of *anti*-**24** (0.100 g, 0.188 mmol) in dry toluene (5 mL) were successively added KCN (0.047 g, 0.760 mmol), 18-crown-6 (0.019 g, 0.244 mmol), and $Pd(PPh_3)_4$ (0.014 g, 0.010 mmol) under argon atmosphere. After heating at 80 °C for 5 h the mixture was cooled to room temperature and subsequently quenched with sat aq. $NaHCO_3$ solution (5 mL). After extraction with EtOAc (3 × 10 mL), the combined organic phases were dried (Na_2SO_4). Purification by flash chromatography (hexanes/EtOAc = 6:1) afforded 5-cyano derivative *anti*-**25** (0.042 g, 52%) as colorless resin.

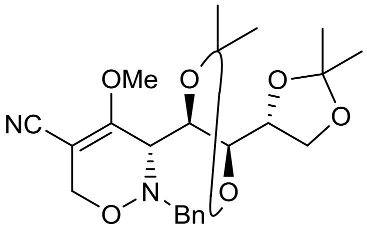

$[\alpha]_D^{23}$ 29.2 (*c* 0.45, $CHCl_3$).

IR (neat): ν = 3065-2850 (=C-H, C-H), 2205 (C≡N) 1645 cm^{-1} (C=C).

^{1}H NMR ($CDCl_3$, 400 MHz): δ = 1.32, 1.36, 1.37, 1.38 (4 s, 12 H, Me), 3.72 (br s, 1 H, 3-H), 3.92-4.05, 4.10-4.15 (2 m, 3 H, 2 H, NCH_2, 3-H, 4''-H, 5'-H, 5''-H), 4.18 (s, 3 H, OMe), 4.21 (d, J = 17.9 Hz, 1 H, NCH_2), 4.26 (d, J = 16.9 Hz, 1 H, 6-H), 4.33 (dd, J = 1.7, 16.9 Hz, 1 H, 6-H), 7.26-7.45 (m, 5 H, Ph).

^{13}C NMR ($CDCl_3$, 101.8 MHz): δ = 25.3, 26.6, 27.0, 27.05 (4 q, 4 Me), 58.4 (t, NCH_2), 58.7 (q, OMe), 63.1 (d, C-3), 65.7 (t, C-6), 67.8 (t, C-5'), 77.6 (d, C-4'), 78.3 (d, C-4''), 79.2 (d, C-5'), 81.4 (s, C-5), 109.70, 109.75 (2 s, C-2', C-2''), 116.7 (s, CN), 127.5, 128.4, 128.5, 136.8 (3 d, s, Ph), 163.7 (s, C-4).

HRMS (ESI-ToF): calcd for $C_{23}H_{31}N_2O_6$ $[M+H]^+$: 431.2177; found 431.2190; $C_{23}H_{30}N_2O_6Na$ $[M+Na]^+$: 453.1996; found 453.2013.

(3*S*,4'*S*)-2-Benzyl-5-(1-benzyl-1*H*-1,2,3-triazol-4-yl)-3-(2,2-dimethyl-1,3-dioxolan-4-yl)-4-methoxy-3,6-dihydro-2*H*-1,2-oxazine (*syn*-26)

A solution of 1,2-oxazine *syn*-**5** (0.100 g, 0.249 mmol) and KF (17.5 mg, 0.300 mmol) in MeOH and THF (8 mL each) was stirred at room temperature for 24 h. After removal of the solvents under reduced pressure, the residue was purified by column chromatography (alumina, neutral, activity grade III; hexanes/EtOAc = 4:1) to give the desilylated product (0.062 g, 76%) as a yellow oil.

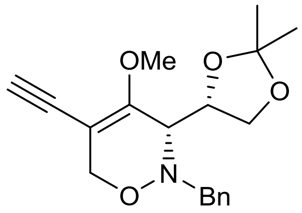

IR (neat): ν = 3090-2885 (=C-H, C-H), 2085 (C≡C), 1645 cm^{-1} (C=C).

^1H NMR (250 MHz, $CDCl_3$): δ = 1.35, 1.38 (2 s, 3 H each, Me), 3.12 (s, 1 H, ≡CH), 3.36 (br d, *J* = 6.9 Hz, 1 H, 3-H), 3.78-3.95 (m, 2 H, 5'-H), 4.02 (s, 3 H, OMe), 4.05 (d, *J* = 13.8 Hz, 1 H, 6-H), 4.11 (s, 2 H, NCH_2), 4.33 (br d, *J* = 13.8 Hz, 1 H, 6-H), 4.48 (dt, *J* = 6.9, 6.0 Hz, 1 H, 4'-H), 7.19-7.42 (m, 5 H, Ph).

^{13}C NMR (100.6 MHz, $CDCl_3$): δ = 26.1, 26.5 (2 q, Me), 58.2 (t, CH_2Ph), 58.5 (q, OMe), 63.0 (d, C-3), 66.6 (t, C-5'), 67.4 (t, C-6), 74.6 (d, C-4'), 78.1, 82.9 (d, s, C≡CH), 93.1 (s, C-5), 108.6 (s, C-2'), 127.2, 128.2, 128.6, 137.4 (3 d, s, Ph), 156.2 (s, C-4).

HRMS (ESI-ToF): calcd for $C_{19}H_{23}NO_4Na$ $[M+Na]^+$ 352.1519; found: 352.1522.

To a solution of the desilylated *syn*-**5** (41 mg, 0.125 mmol) in MeCN (5 mL) benzyl azide (17 mg, 0.125 mmol), TBTA (16.1 mg, 0.03 mmol), triethylamine (6 µL, 0.03 mmol), and CuI (3 mg, 0.03 mmol) were added at room temperature and stirred for 1 d. After filtration of the mixture (silica gel), concentration in vacuo and purification by column chromatography (silica gel, hexanes/EtOAc, 2:1) afforded the product *syn*-**26** (31 mg, 54%) as a pale yellow oil.

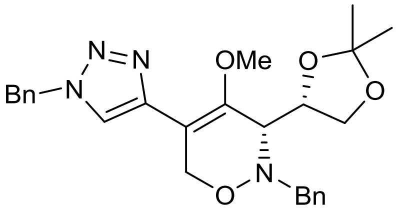

$[\alpha]_D^{22}$ 109.7 (*c* 0.85, $CHCl_3$).

IR (neat): ν = 3065-2870 (=C-H, C-H), 1650, 1500 cm^{-1} (C=C).

^{1}H NMR (400 MHz, $CDCl_3$): δ = 1.34, 1.36 (2 s, 3 H each, Me), 3.59 (s, 3 H, OMe), 3.74 (br d, *J* = 5.6 Hz, 1 H, 3-H), 3.87 (dd, *J* = 7.6, 8.8 Hz, 1 H, 5'-H), 4.02 (dd, *J* = 6.0, 8.8 Hz, 1 H, 5'-H), 4.14, 4.17 (AB system, J_{AB} = 13.7 Hz, 1 H each, CH_2N), 4.55-4.62 (m, 1 H, 4'-H), 4.72 (d, *J* = 15.3 Hz, 1 H, 6-H), 4.94 (dd, *J* = 1.4, 15.3 Hz, 1 H, 6-H), 5.54 (s, 2 H, CH_2N), 7.19-7.42 (m, 10 H, Ph), 7.80 (s, 1 H, $=CH_{triazole}$).

^{13}C NMR (100.6 MHz, $CDCl_3$): δ = 25.8, 26.4 (2 q, Me), 54.0 (t, N*C*H_2Ph), 56.0 (d, C-3), 58.0 (t, N*C*H_2Ph), 58.6 (q, OMe), 64.8 (t, C-6), 66.5 (t, C-5'), 75.6 (d, C-4'), 108.5, 108.8 (2 s, C-5, C-2'), 122.5 (d, $=CH_{triazole}$), 127.3, 127.8, 128.3, 128.6, 129.0, 134.9, 137.2, 141.8, 146.7 (5 d, 4 s, Ph, C-4, $C_{triazole}$).

Anal. calcd. for $C_{26}H_{30}N_4O_4$ (462.5): C 67.51, H 6.54, N 12.11; found: C 67.08, H 6.29, N 11.79.

Hydrogenation of 1,2-oxazine *syn*-**21**

A suspension of Pd on charcoal (10% Pd; 0.157 g) in MeOH (12 mL) was saturated with hydrogen for 1 h. Then, 1,2-oxazine *syn*-**21** (0.157 g, 0.381 mmol), dissolved in MeOH (5 mL), was added and the mixture was stirred under an hydrogen atmosphere for 1 d at room temperature. Filtration through a pad of celite, followed by concentration in vacuo and flash chromatography (CH_2Cl_2/MeOH, 95:5) afforded amino alcohol **27** (0.063 g, 51%; 2 diastereomers = 96:4) as a colorless oil.

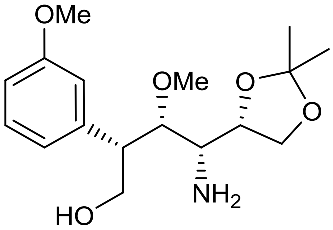

IR (neat): ν = 3400-3200 (O-H, N-H), 3085-2850 cm^{-1} (=C-H, C-H).

^{1}H NMR (CD_3OD, 500 MHz): δ = 1.29, 1.31 (2 s, 3 H each, Me), 2.47 (dd, *J* = 1.4, 7.5 Hz, 1 H, 3-H), 3.03 (dt, *J* = 6.1, 8.4 Hz, 1 H, 4'-H) 3.41 (dd, *J* = 1.4, 8.4 Hz, 1 H, 4-H), 3.45 (s, 3 H, OMe), 3.53 (m_c, 1 H, 1-H), 3.79 (s, 3 H, OMe), 3.83 (dd, *J* = 6.1, 10.8

Hz, 1 H, 5‘-H), 3.98 (dd, J = 5.2, 10.8 Hz, 1 H, 5’-H), 4.09 (m_c, 2 H, 1-H, 2-H), 6.79-6.82, 6.86-6.89, 7.22-7.25 (3 m, 5 H, Ph).

^{13}C NMR (CD_3OD, 125 MHz): δ = 25.9, 27.0 (2 q, Me), 52.6 (d, C-4‘), 55.7 (d, C-3), 56.7 (q, OMe), 61.6 (q, OMe), 63.6 (t, C-5‘), 68.0 (t, C-1), 79.5 (d, C-2), 84.3 (d, C-4), 110.4 (s, C-2’), 113.3, 115.6, 121.9, 130.7, 144.1, 161.4 (4 d, 2 s, Ph).

Additional signals assigned to the minor isomer:

^{1}H NMR (CD_3OD, 500 MHz): δ = 1.295, 1.300 (2 s, 3 H each, Me), 3.48 (s, 3 H, OMe), 3.63-3.70 (m, 1 H), 3.78 (s, 3 H, OMe).

HRMS (ESI-ToF): calcd for $C_{17}H_{21}NO_5Na$ $[M+Na]^+$ 348.1781; found: 348.1759.

(2*R*,4’*S*)-2-(2,2-Dimethyl-[1,3]dioxolan-4-yl)-1-methanesulfonyl-3-methoxy-4-(3-methoxyphenyl)pyrrolidine (28)

To a solution of amino alcohol **27** (0.056 g, 0.172 mmol; dr 96:4) dissolved in dry CH_2Cl_2 (5 mL) MsCl (0.03 mL, 0.043 g, 0.379 mmol) and NEt_3 (0.06 mL, 0.042 g, 0.413 mmol) were added. After stirring under argon atmosphere at the room temperature for 4 h water was added. The two layers were separated and the aqueous layer was extracted with CH_2Cl_2 and the combined extracts were dried with $MgSO_4$. Removal the solvent in vacuo yielded crude product, which was purified by FLC (*i*-hexane: isopropyl alcohol (3:1) to afford pyrrolidine derivative **29** (43 mg, 65%; 2 diastereomers = 95:5) as colorless oil.

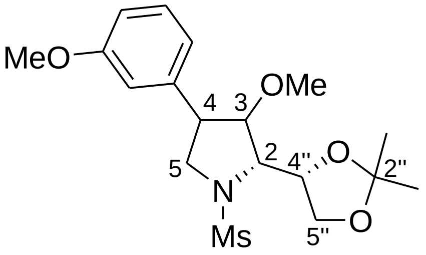

^{1}H NMR ($CDCl_3$, 500 MHz): δ = 1.33, 1.34 (2 s, 3 H each, Me), 3.02 (s, 3 H, Ms), 3.31 (ddd, J = 3.5, 5.9, 9.5 Hz, 1 H, 4-H), 3.38 (ddd, J = 2.0, 6.7, 9.5 Hz, 1 H, 2-H), 3.46 (dd, J = 2.0, 9.5 Hz, 1 H, 3-H), 3.49 (dd, J = 7.3, 8.4 Hz, 1 H, 5‘-H), 3.54 (s, 3 H, OMe), 3.81 (s, 3 H, OMe), 4.04 (dd, J = 6.7, 8.4 Hz, 1 H, 5‘-H), 4.20 (dt, J = 6.7, 7.3 Hz, 1 H, 4’-H), 4.41 (dd, J = 3.5, 9.6 Hz, 1 H, 5-H), 4.65 (dd, J = 5.9, 9.6 Hz, 1 H, 5-H) 6.83-6.89, 7.26-7.29 (2 m, 4 H, Ph).

^{13}C NMR ($CDCl_3$, 125 MHz): δ = 25.3, 26.2 (2 q, Me), 42.5 (q, Ms), 47.3 (d, C-4), 55.2 (q, OMe), 56.8 (C-2), 61.0 (q, OMe), 66.7 (t, C-5‘), 70.8 (t, C-5), 76.1 (d, C-4‘), 81.4 (d, C-3), 109.7 (s, C-2’), 113.4, 115.2, 120.7, 129.8, 138.9, 159.8 (4 d, 2 s, Ph).

A complete characterization of **29** was not possible due to the sensitivity of the product.

Hydrogenation of 1,2-oxazine *anti*-**24**

According to the hydrogenation of *syn*-**21**, a suspension of Pd on charcoal (10% Pd; 0.099 g) in MeOH (10 mL) was saturated with hydrogen for 1 h. Then, 1,2-oxazine *anti*-**24** (0.300 g, 0.623 mmol), dissolved in MeOH (5 mL), was added and the mixture was stirred under an hydrogen atmosphere at room temperature for 1 h. Filtration through a pad of celite, followed by concentration in vacuo and flash chromatography (hexanes/ EtOAc, 6:1 to 0:1) afforded *anti*-**29** (0.090 g, 37%) as colorless foam, *anti*-**30** (0.087 g, 36%; 2 diastereomers = 93:7) as pale yellow wax, and starting material *anti*-**24** (0.040 g, 17%).

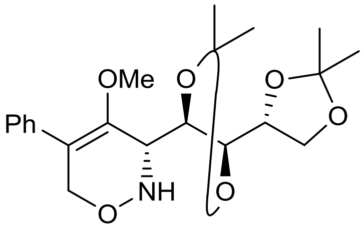

$[\alpha]_D^{22}$ 31.3 (*c* 0.5, $CHCl_3$).

IR (neat): ν = 3300 (N-H), 3065-2870 (=C-H, C-H), 1600 cm^{-1} (C=C).

^{1}H NMR (500 MHz, $CDCl_3$): δ = 1.35 (s, 3 H, Me), 1.40 (s, 3 H, Me), 1.40 (s, 3 H, Me), 1.45 (s, 3 H, Me), 3.39 (s, 3 H, OMe), 3.80 (d_{br}, *J* = 4.6 Hz, 1 H, 3-H), 3.99 (dd, *J* = 6.4, 8.2 Hz, 1 H, 5''-H), 4.11 (dd, *J* = 6.4, 8.2 Hz, 1 H, 5''-H), 4.16 (q, *J* = 6.4 Hz, 1 H, 4''-H), 4.26 (dd, *J* = 4.7, 7.8 Hz, 1 H, 5'-H), 4.32 (dd, *J* = 6.4, 7.8 Hz, 1 H, 4'-H), 4.43 (d, *J* = 14.6 Hz, 1 H, 6-H), 4.53 (dd, *J* = 1.8, 14.6 Hz, 1 H, 6-H), 6.25 (s_{br}, 1 H, NH), 7.22-7.27, 7.30-7.38 (2 m, 1 H, 4 H, Ph).

^{13}C NMR (126 MHz, $CDCl_3$) δ = 25.6, 26.6, 27.09, 27.12 (4 q, Me), 57.6 (d, C-3), 59.0 (q, OMe), 66.9 (t, C-5''), 70.3 (t, C-6), 76.7 (d, C-4''), 79.1 (d, C-4'), 80.1 (d, C-5'), 109.1 (s, C-2''), 109.7 (s, C-2'), 117.4 (s, C-5), 127.4, 128.4, 128.4, 135.4 (3 d, s, Ph), 148.4 (s, C-4).

HRMS (pos. ESI): calcd for $C_{21}H_{29}NO_6$ $[M+H]^+$ 392.2068; found: 392.2072.

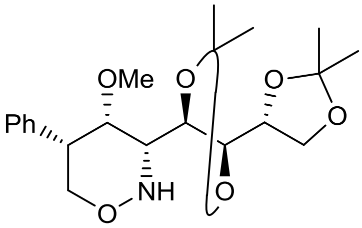

IR (neat): ν = 3310 (N-H), 3100-2850 cm^{-1} (=C-H, C-H).

^{1}H NMR (500 MHz, $CDCl_3$): δ = 1.325, 1.330, 1.40, 1.45 (4 s, 12 H, Me), 2.97-3.04 (m, 4 H, OMe, 5-H), 3.28 (d_{br}, *J* = 9.5 Hz, 1 H, 3-H), 3.66 (s_{br}, 1 H, 4-H), 3.80 (dd, *J* = 6.4, 9.6 Hz, 1 H, 5'-H), 3.88-3.98 (m, 3 H, 4'-H, 5''-H, 6-H), 4.03-4.13 (m, 2 H, 4''-H, 5''-H), 4.32 (t, *J* = 11.6 Hz, 1 H, 6-H), 5.81 (s_{br}, 1 H, NH), 7.22-7.33 (m, 5 H, Ph).
^{13}C NMR (126 MHz, $CDCl_3$): δ = 25.4, 26.6, 27.2, 27.5 (4 q, Me), 46.6 (d, C-5), 60.6 (q, OMe), 65.0 (d, C-3), 67.4 (t, C-5''), 69.6 (t, C-6), 76.0 (d, C-4''), 76.19 (d, C-5'), 76.23 (d, C-4), 81.6 (d, C-4'), 109.2 (s, C-2''), 110.0 (s, C-2'), 127.1, 128.5, 128.6, 139.3 (3 d, s, Ph).
HRMS (pos. ESI): calcd for $C_{21}H_{31}NO_6$ $[M+H]^+$ 394.2224; found: 394.2247.
