# Supplementary material for: Iodination of carbohydrate-derived 1,2-oxazines to enantiopure 5-iodo-3,6-dihydro-2H-1,2-oxazines and subsequent palladium-catalyzed cross-coupling reactions
Source: Beilstein J Org Chem. 2016 Dec 29;12:2898–905. doi: 10.3762/bjoc.12.289 (PMC5238527; doi:10.3762/bjoc.12.289)
Supplement: File 2 — Copies of 1H and 13C NMR spectra of compounds 4–9, 11, 14, 19, 21, 24–27, 29 and 30. [file Beilstein_J_Org_Chem-12-2898-s002.pdf]

## Supporting Information

**for**

# Iodination of carbohydrate-derived 1,2-oxazines to enantiopure 5-iodo-3,6-dihydro-2*H*-1,2-oxazines and subsequent palladium-catalyzed cross-coupling reactions

Michal Medvecký[1], Igor Linder[1], Luise Schefzig[1], Hans-Ulrich Reissig*[1] and Reinhold Zimmer*[1]

Address: [1]Freie Universität Berlin, Institut für Chemie und Biochemie, Takustrasse 3, D-14195 Berlin, Germany

Email: Hans-Ulrich Reissig - hans.reissig@chemie.fu-berlin.de, Reinhold Zimmer - rzimmer@zedat.fu-berlin.de

* Corresponding author

## Copies of $^{1}$H and $^{13}$C NMR spectra of compounds 4–9, 11, 14, 19, 21, 24–27, 29 and 30

NMR data of new compounds

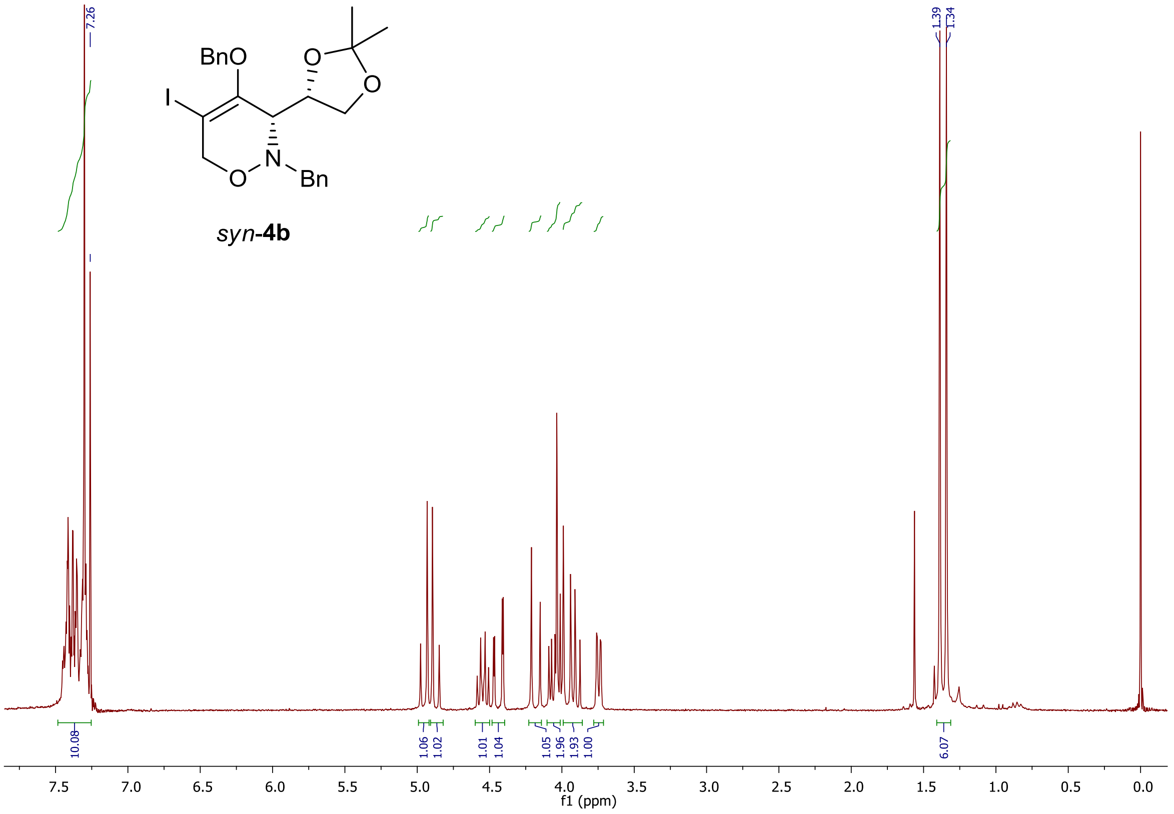


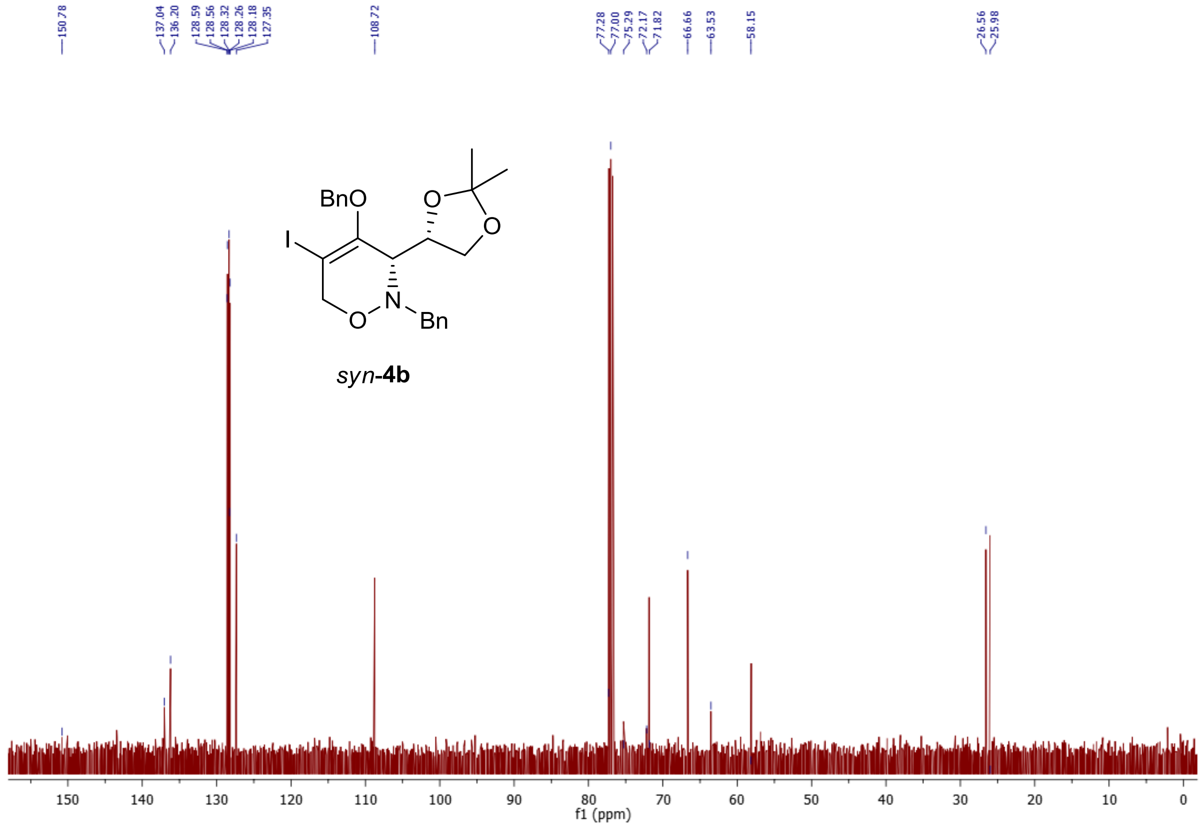

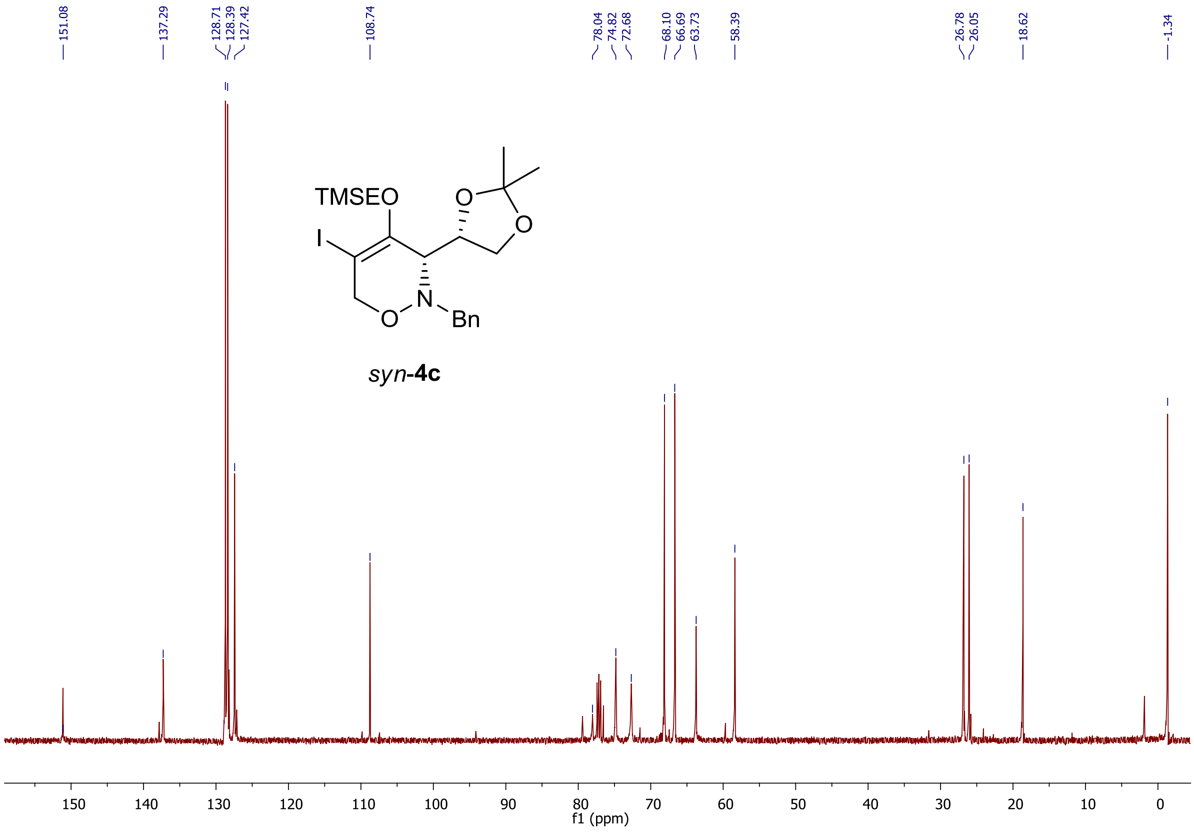
151.08
137.29
128.71
128.39
127.42
108.74
78.04
74.82
72.68
68.10
66.69
63.73
58.39
26.78
26.05
18.62
-1.34
TMSEO
I
O
O
N
O
Bn
syn-4c
150 140 130 120 110 100 90 80 70 60 50 40 30 20 10 0
f1 (ppm)

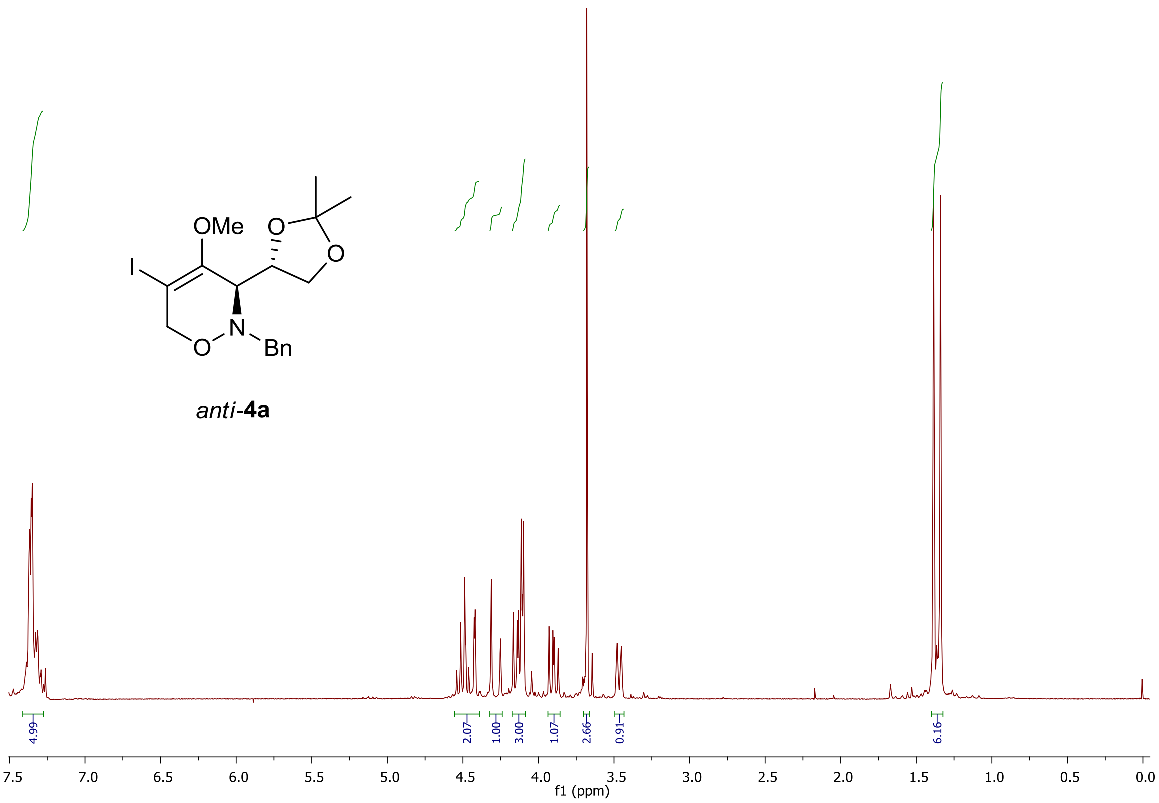
OMe
I
O
O
N
O
Bn
anti-4a
4.99
2.07
1.00
3.00
1.07
2.66
0.91
6.16
7.5 7.0 6.5 6.0 5.5 5.0 4.5 4.0 3.5 3.0 2.5 2.0 1.5 1.0 0.5 0.0
f1 (ppm)

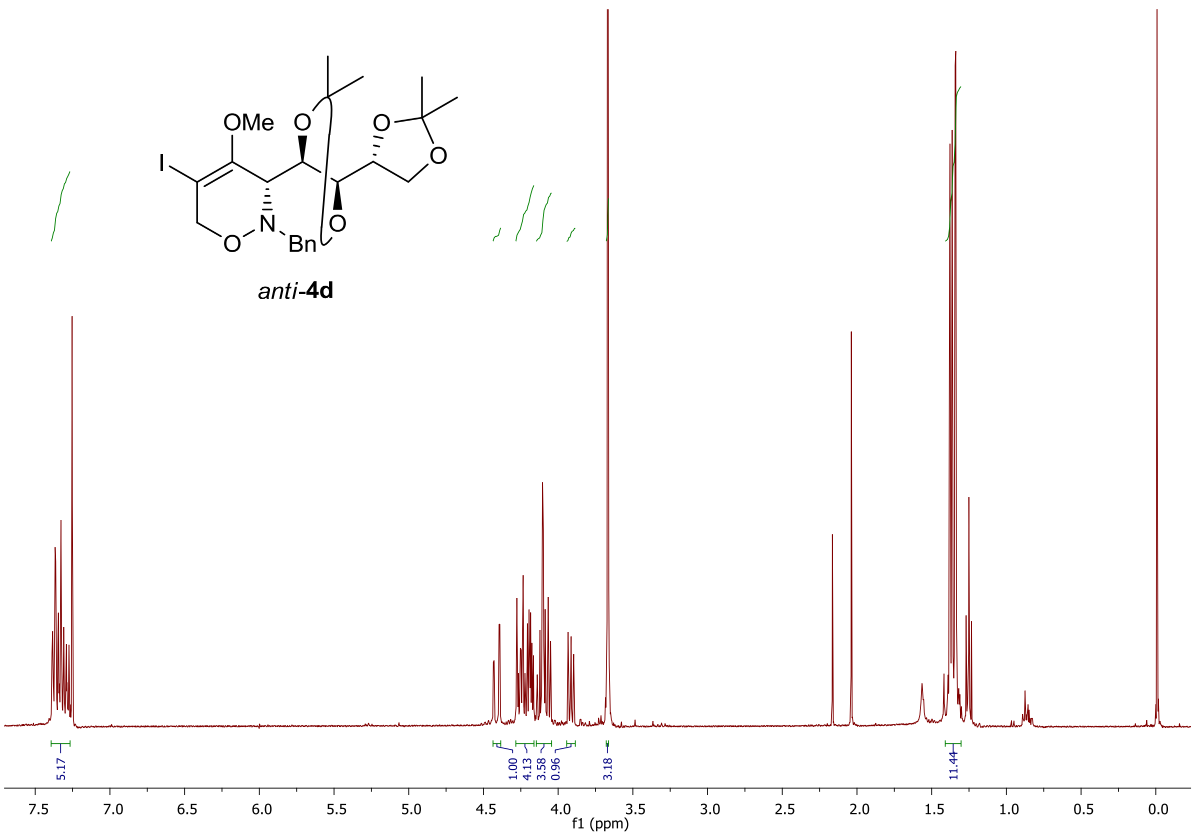
OMe
I
O
N
Bn
O
O
O
O
anti-4d
5.17
1.00
4.13
3.58
0.96
3.18
11.44
7.5
7.0
6.5
6.0
5.5
5.0
4.5
4.0
3.5
3.0
2.5
2.0
1.5
1.0
0.5
0.0
f1 (ppm)

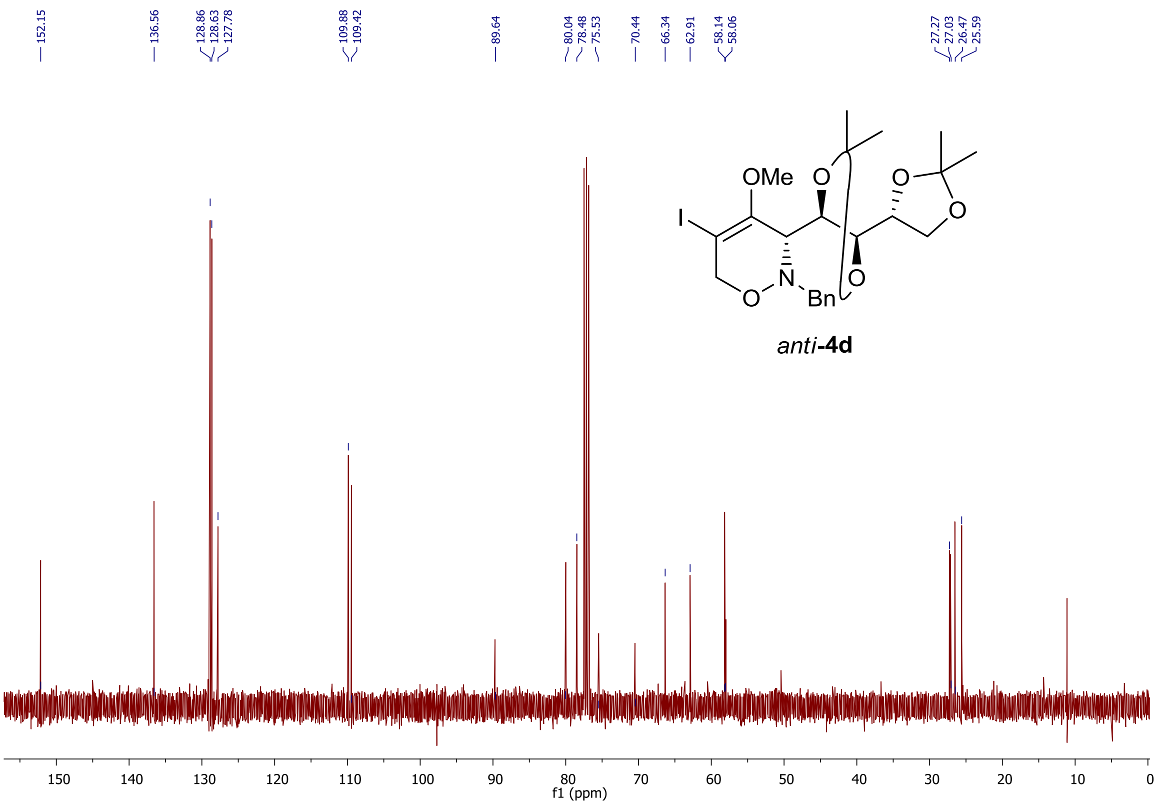
152.15
136.56
128.86
128.63
127.78
109.88
109.42
89.64
80.04
78.48
75.53
70.44
66.34
62.91
58.14
58.06
27.27
27.03
26.47
25.59
OMe
I
O
N
Bn
O
O
O
O
anti-4d
150
140
130
120
110
100
90
80
70
60
50
40
30
20
10
0
f1 (ppm)

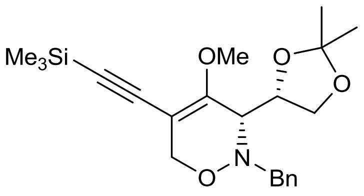
Me3Si
OMe
O
O
N
O
Bn

*syn*-**5**

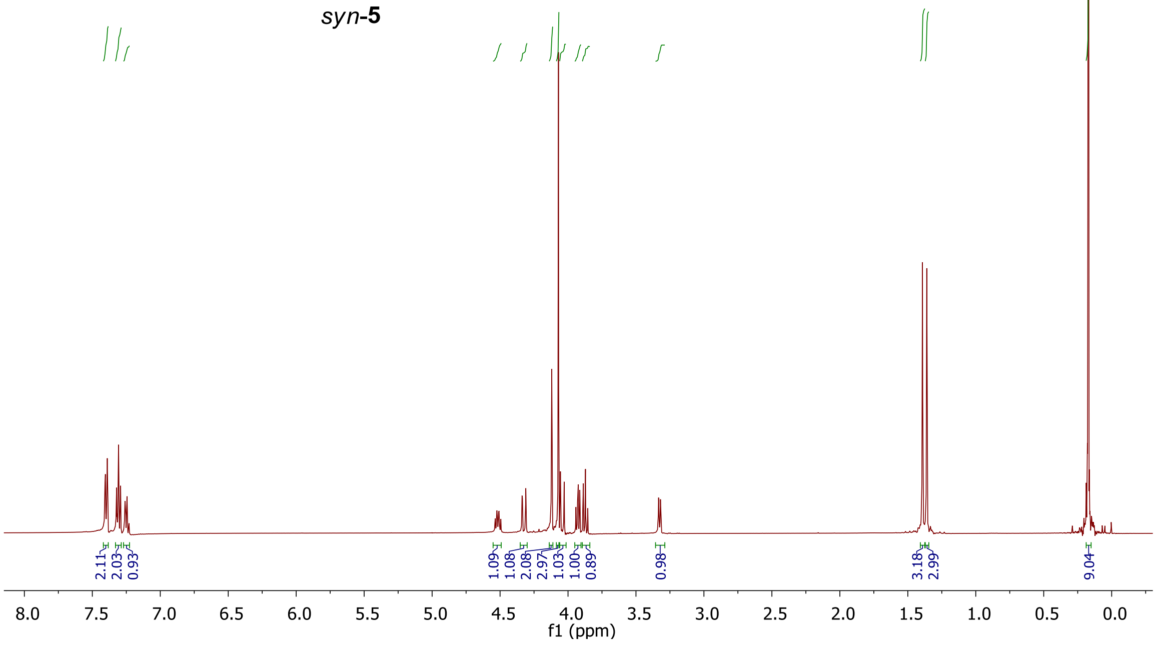
2.11
2.03
0.93
1.09
1.08
2.08
2.97
1.03
1.00
0.89
0.98
3.18
2.99
9.04
8.0 7.5 7.0 6.5 6.0 5.5 5.0 4.5 4.0 3.5 3.0 2.5 2.0 1.5 1.0 0.5 0.0
f1 (ppm)

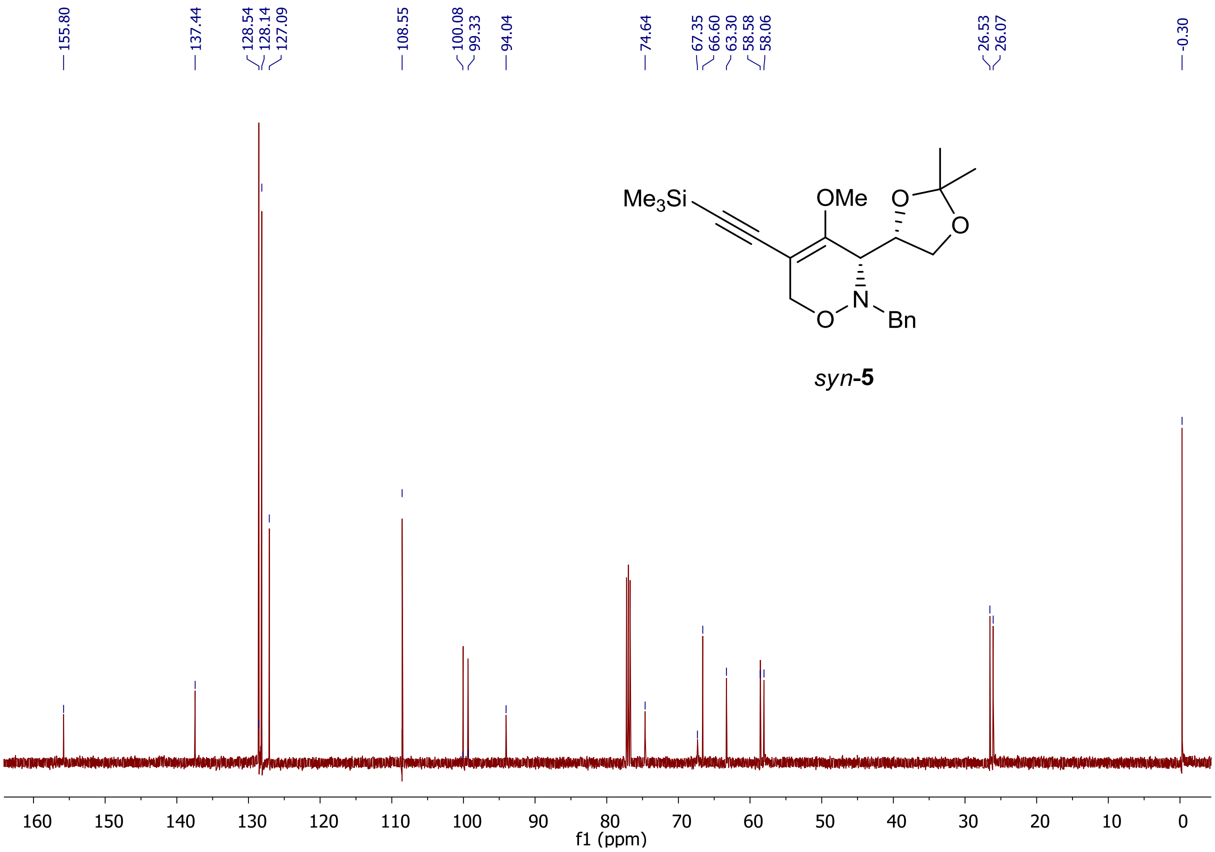
155.80
137.44
128.54
128.14
127.09
108.55
100.08
99.33
94.04
74.64
67.35
66.60
63.30
58.58
58.06
26.53
26.07
-0.30
160 150 140 130 120 110 100 90 80 70 60 50 40 30 20 10 0
f1 (ppm)
Me3Si
OMe
O
O
N
O
Bn
syn-5

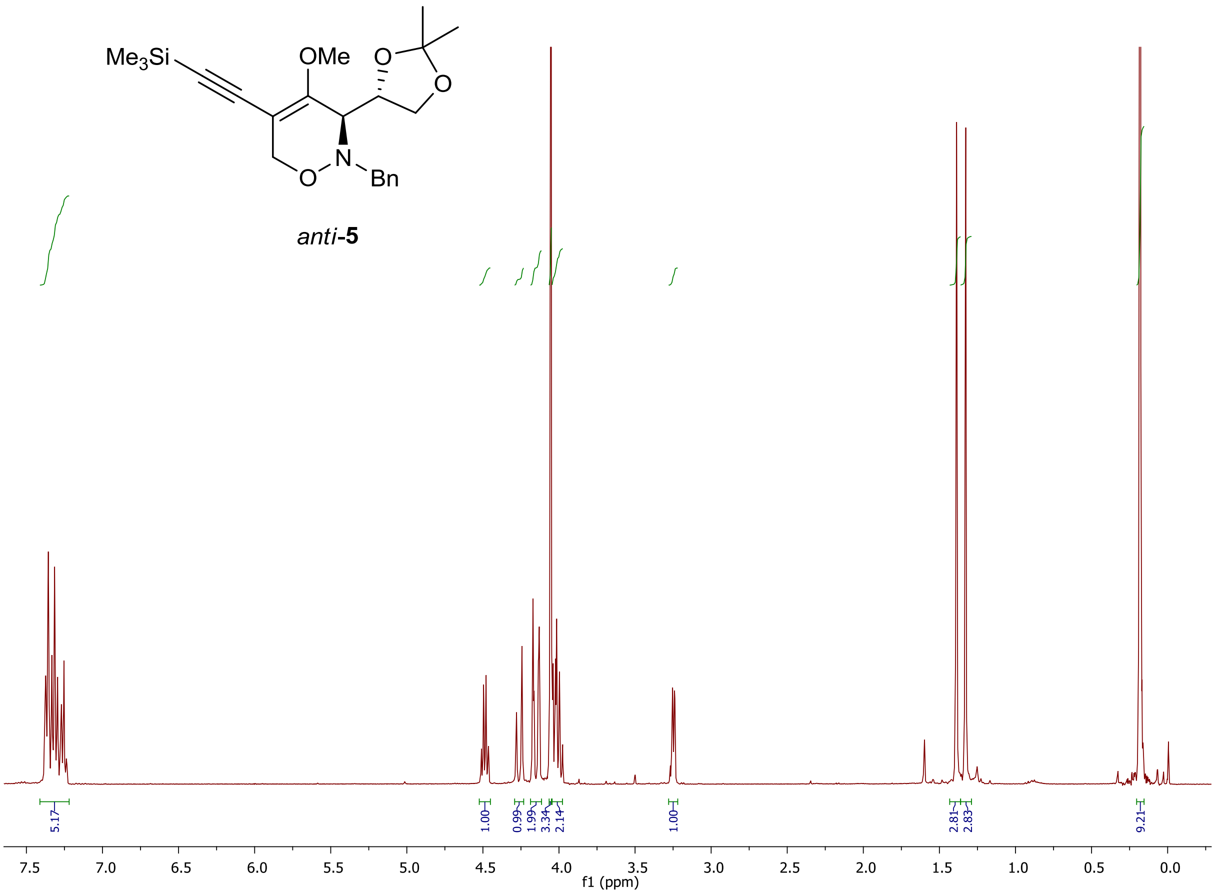
Me3Si
OMe
O
O
N
O
Bn
anti-5
5.17
1.00
0.99
1.99
3.34
2.14
1.00
2.81
2.83
9.21
f1 (ppm)

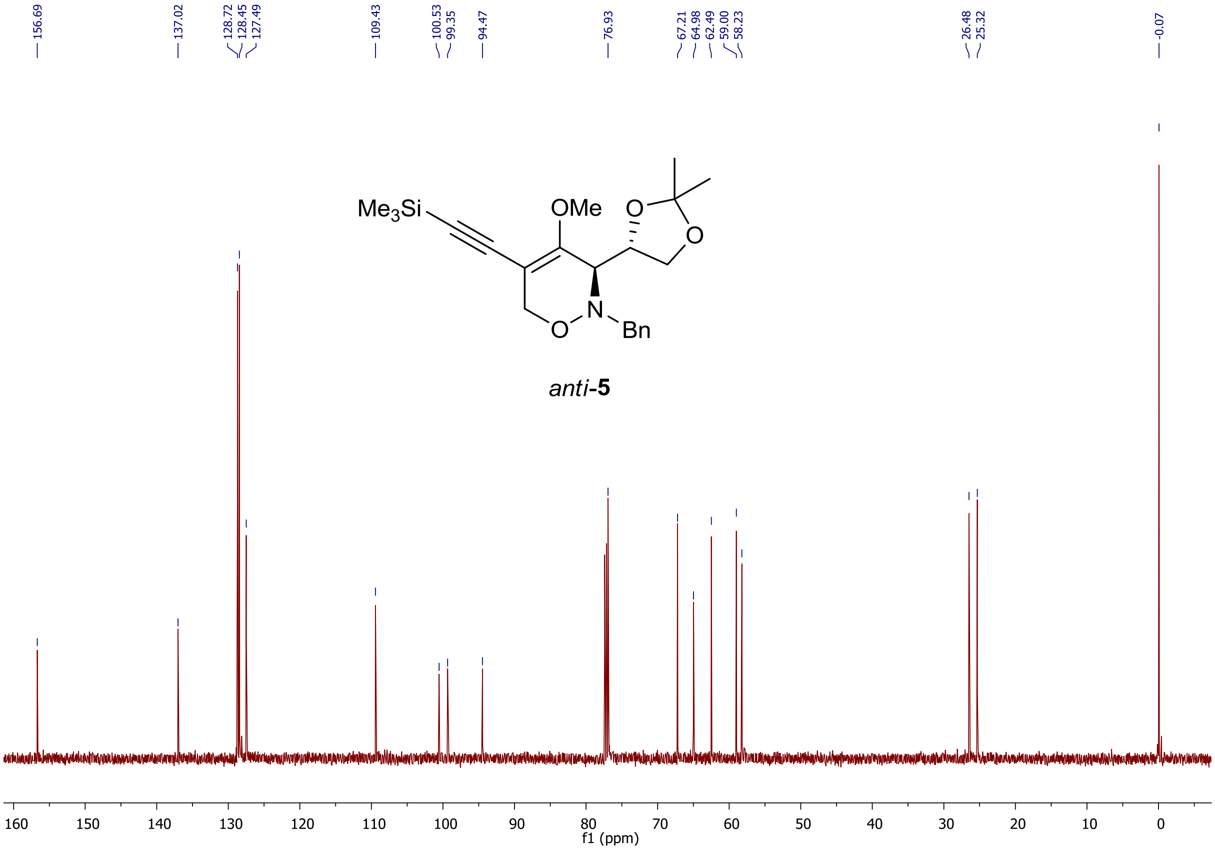
156.69
137.02
128.72
128.45
127.49
109.43
100.53
99.35
94.47
76.93
67.21
64.98
62.49
59.00
58.23
26.48
25.32
-0.07
Me3Si
OMe
O
O
N
O
Bn
anti-5
f1 (ppm)

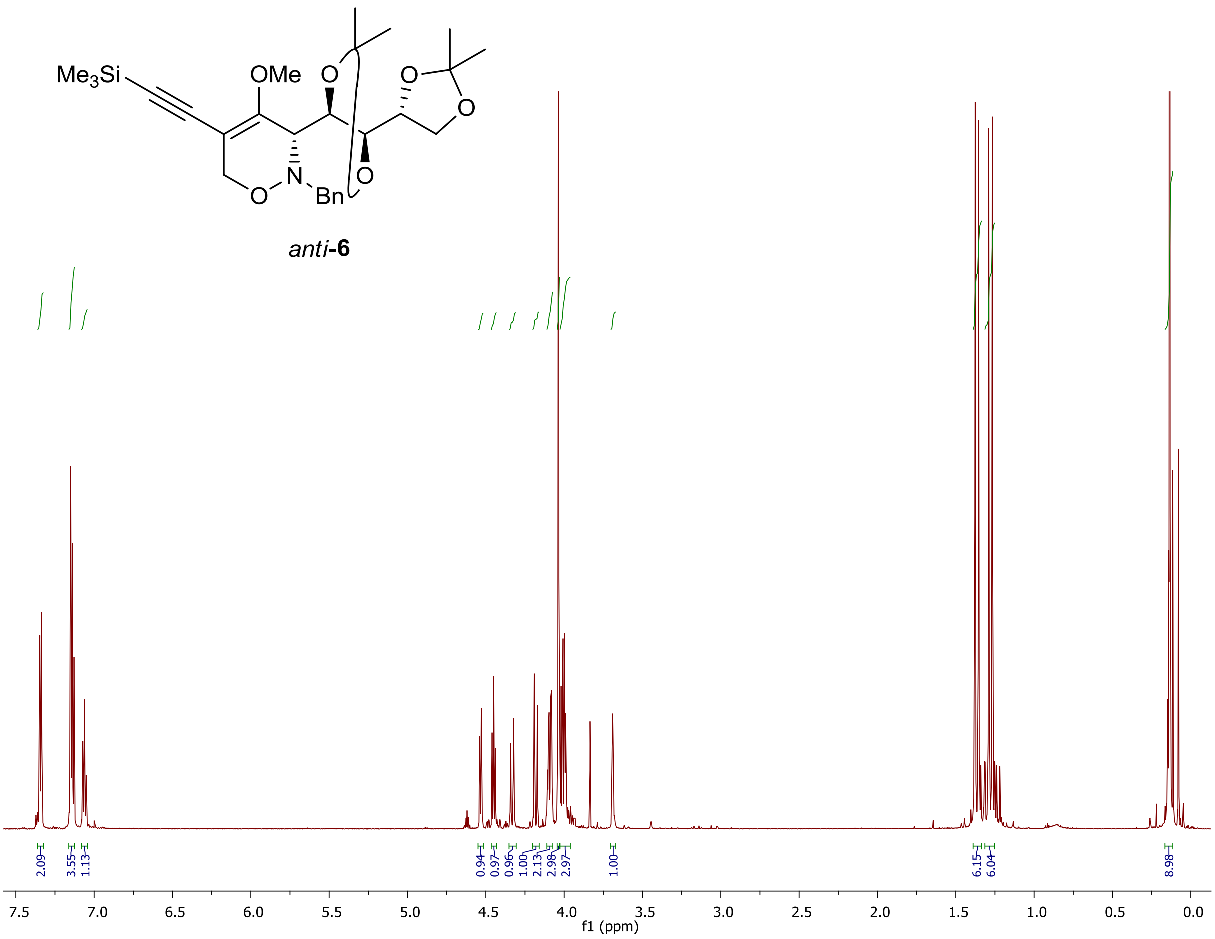
Me3Si
OMe
O
O
O
N
Bn
O
O
anti-6
2.09
3.55
1.13
0.94
0.97
0.96
1.00
2.13
2.98
2.97
1.00
6.15
6.04
8.98
f1 (ppm)

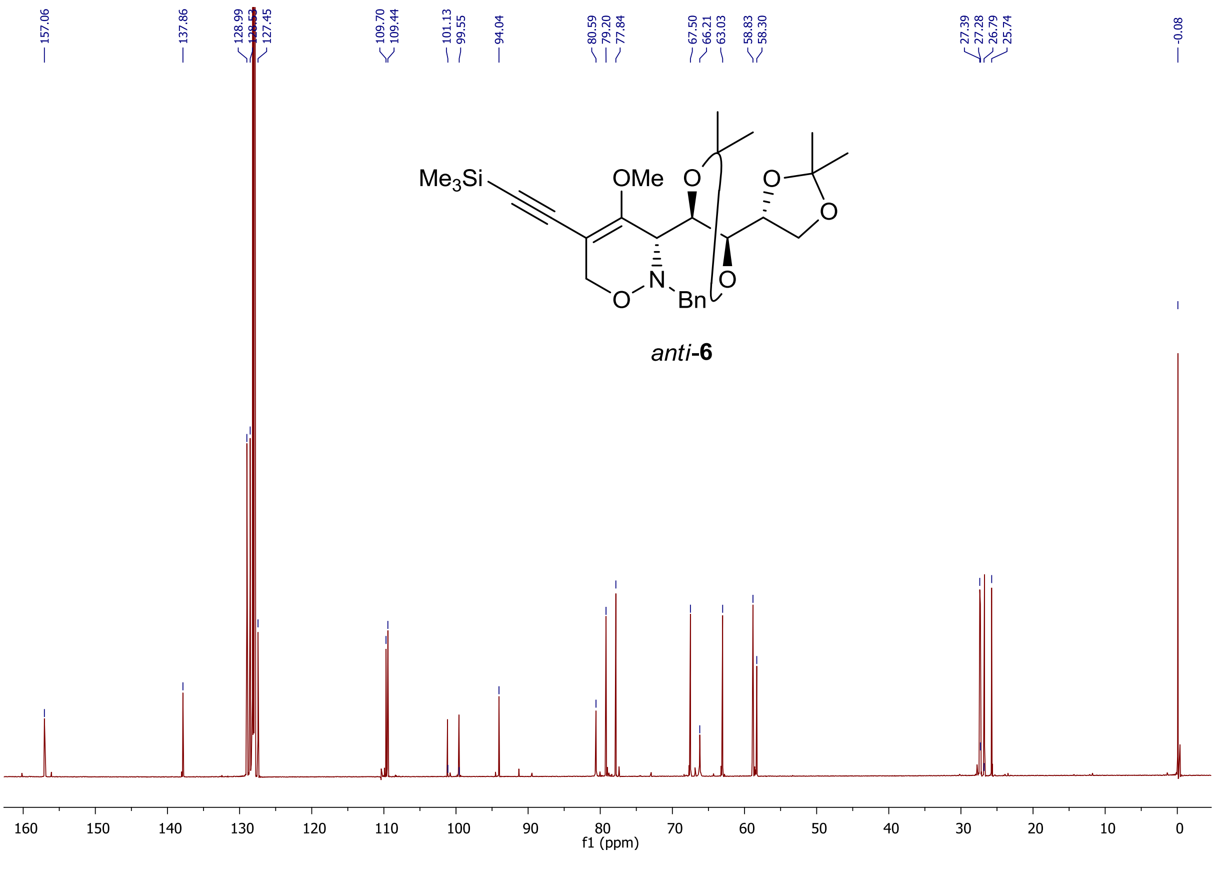
157.06
137.86
128.99
127.45
109.70
109.44
101.13
99.55
94.04
80.59
79.20
77.84
67.50
66.21
63.03
58.83
58.30
27.39
27.28
26.79
25.74
-0.08
Me3Si
OMe
O
O
O
N
Bn
O
O
anti-6
f1 (ppm)

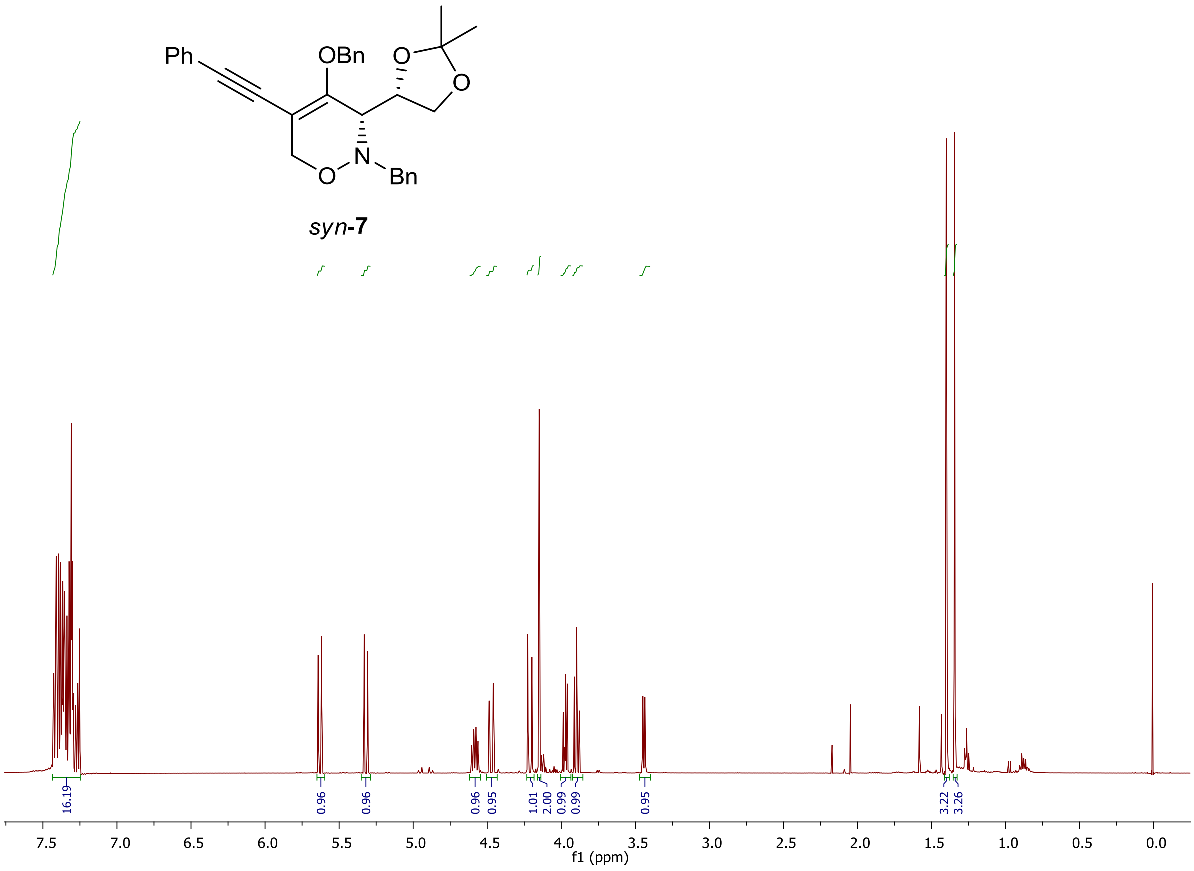
Ph
OBn
O
O
N
O
Bn
syn-7
16.19
0.96
0.96
0.96
0.95
1.01
2.00
0.99
0.99
0.95
3.22
3.26
f1 (ppm)

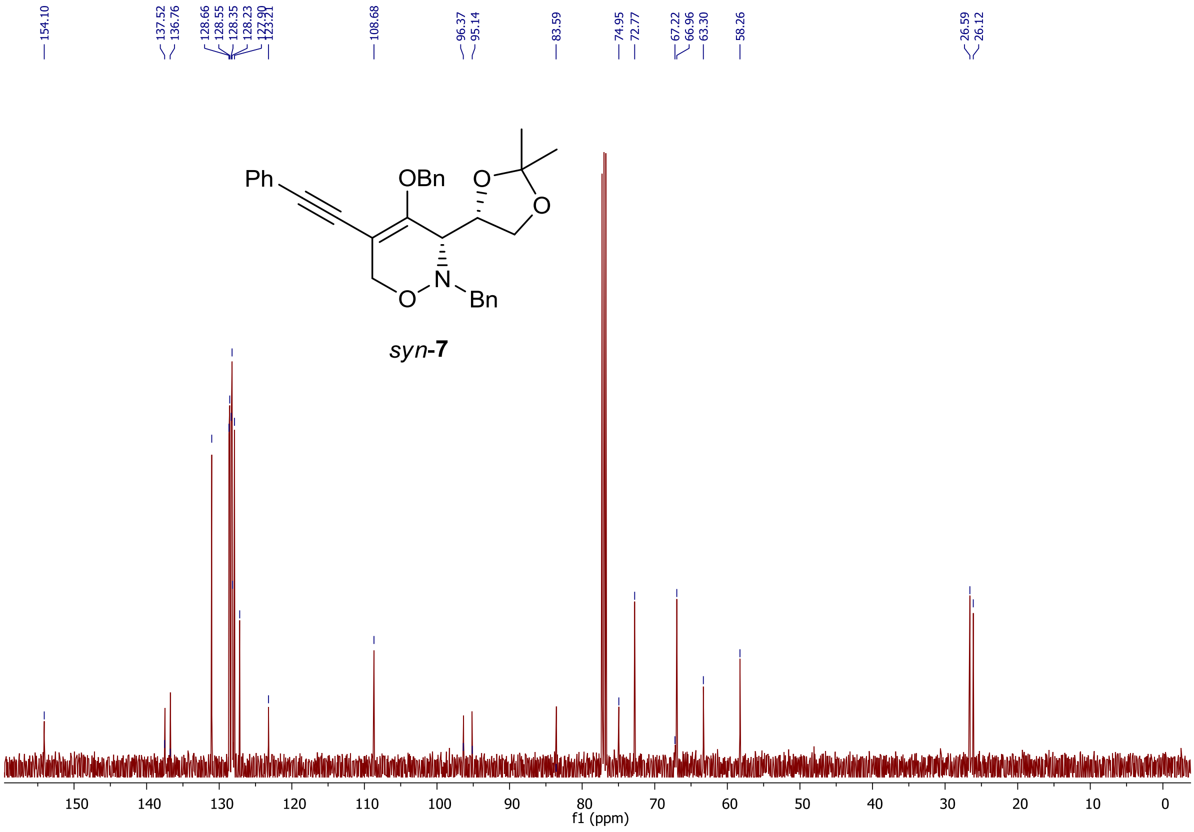
154.10
137.52
136.76
128.66
128.55
128.35
128.23
127.90
123.21
108.68
96.37
95.14
83.59
74.95
72.77
67.22
66.96
63.30
58.26
26.59
26.12
Ph
OBn
O
O
N
O
Bn
syn-7
f1 (ppm)

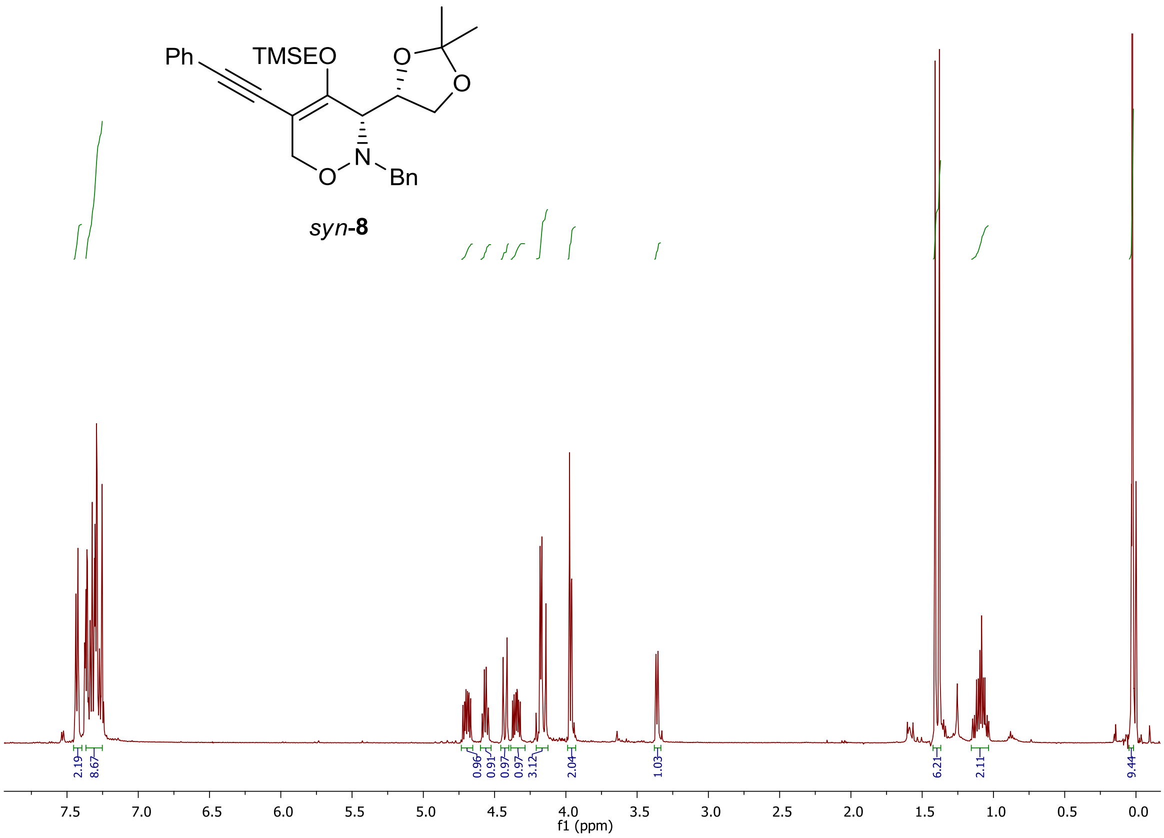
Ph
TMSEO
O
O
N
O
Bn
syn-8
2.19
8.67
0.96
0.91
0.97
0.97
3.12
2.04
1.03
6.21
2.11
9.44
7.5 7.0 6.5 6.0 5.5 5.0 4.5 4.0 3.5 3.0 2.5 2.0 1.5 1.0 0.5 0.0
f1 (ppm)

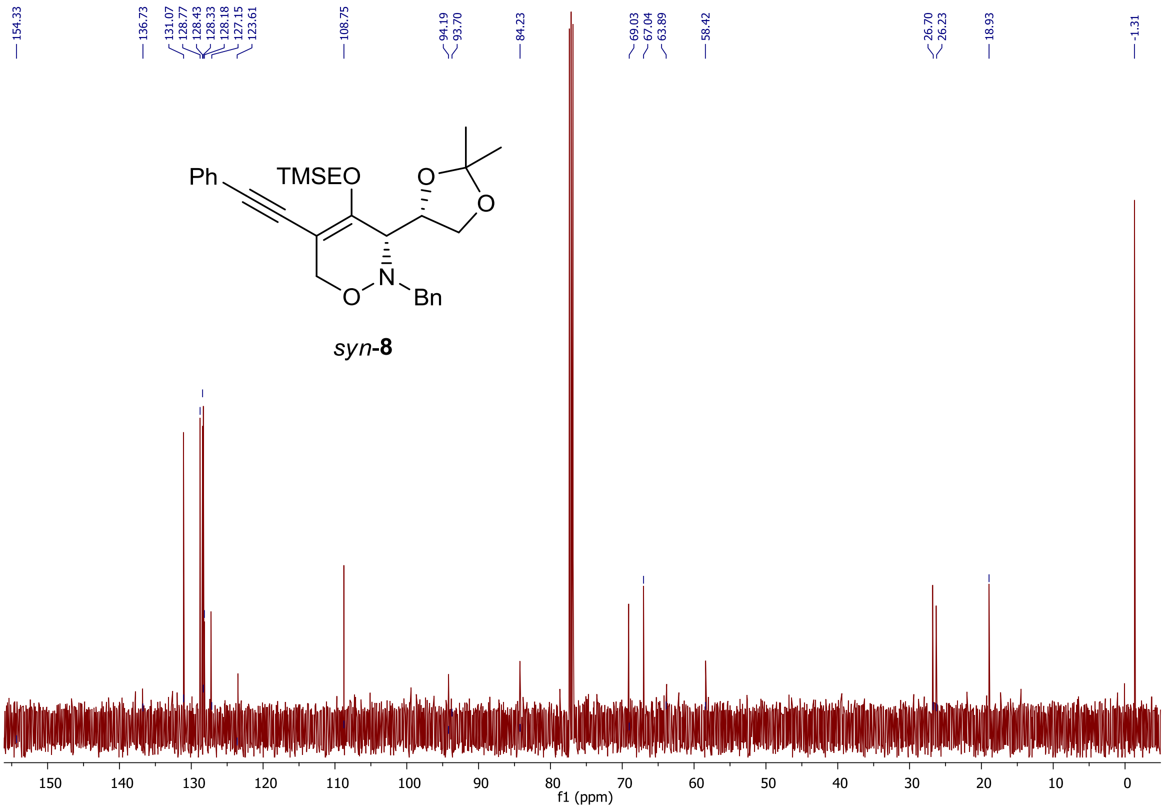
154.33
136.73
131.07
128.77
128.43
128.33
128.18
127.15
123.61
108.75
94.19
93.70
84.23
69.03
67.04
63.89
58.42
26.70
26.23
18.93
-1.31
Ph
TMSEO
O
O
N
O
Bn
syn-8
150 140 130 120 110 100 90 80 70 60 50 40 30 20 10 0
f1 (ppm)

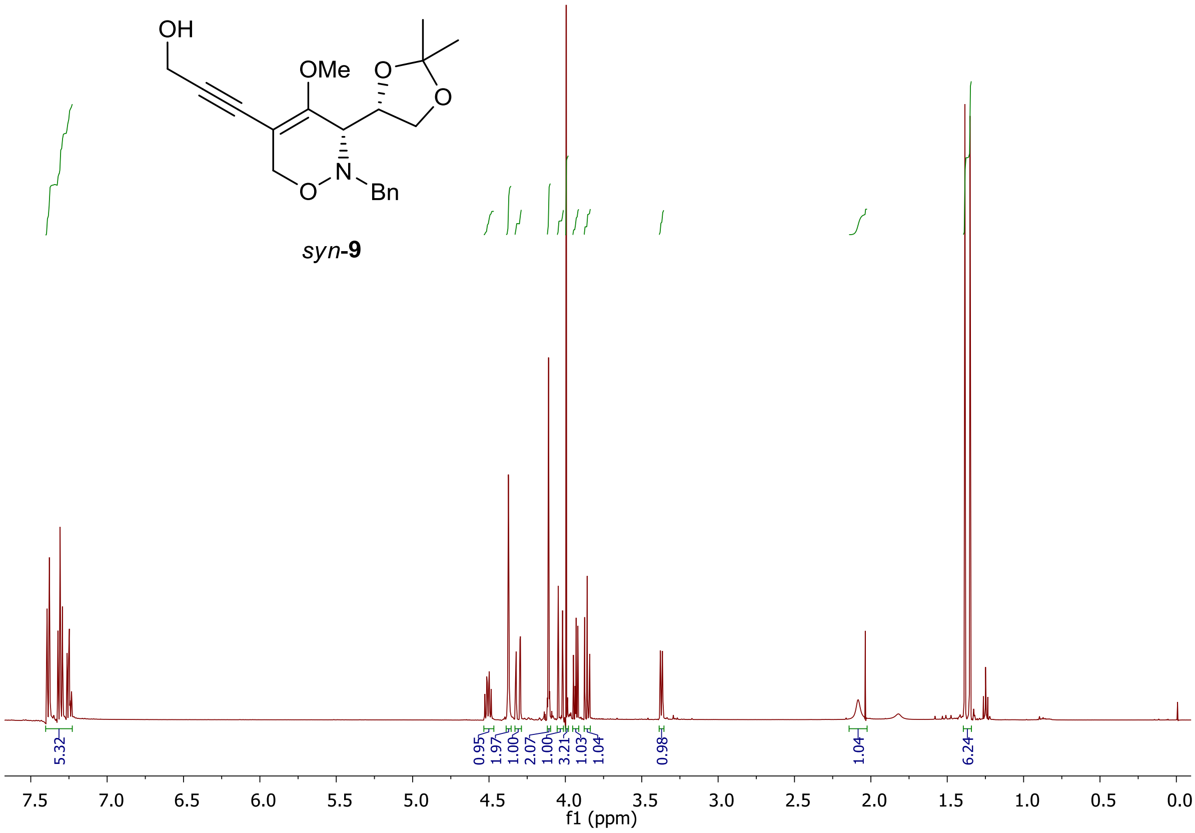
OH
OMe
O
O
N
O
Bn
syn-9
5.32
0.95
1.97
1.00
2.07
1.00
3.21
1.03
1.04
0.98
1.04
6.24
7.5 7.0 6.5 6.0 5.5 5.0 4.5 4.0 3.5 3.0 2.5 2.0 1.5 1.0 0.5 0.0
f1 (ppm)

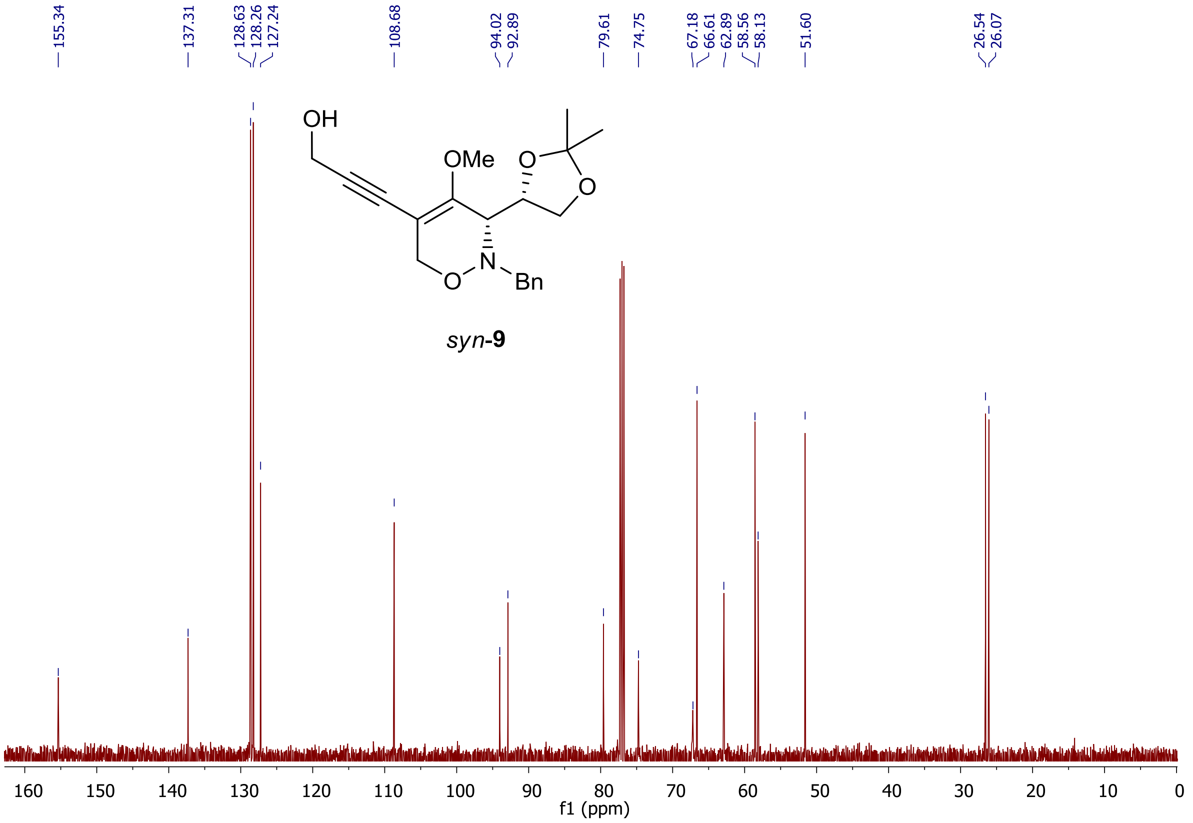
155.34
137.31
128.63
128.26
127.24
108.68
94.02
92.89
79.61
74.75
67.18
66.61
62.89
58.56
58.13
51.60
26.54
26.07
OH
OMe
O
O
N
O
Bn
syn-9
160 150 140 130 120 110 100 90 80 70 60 50 40 30 20 10 0
f1 (ppm)

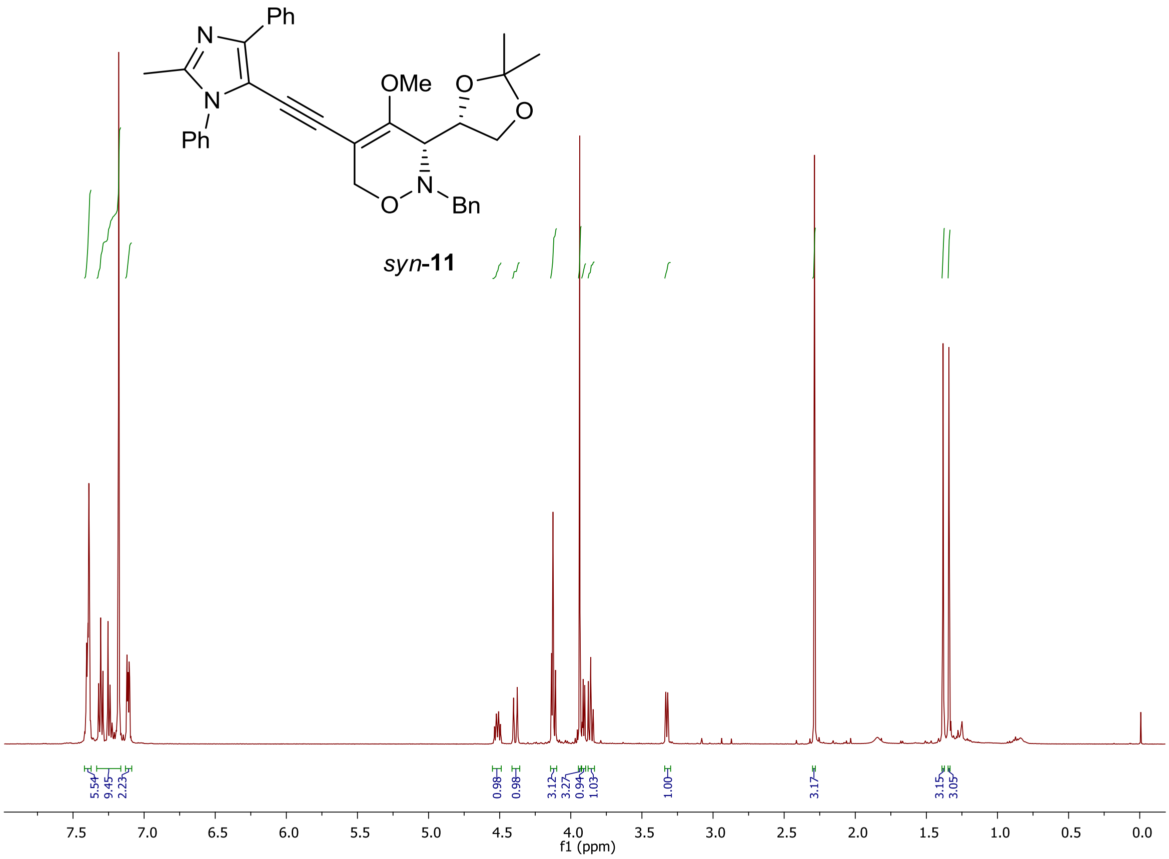

Ph
N
N
Ph
OMe
O
O
O
N
Bn
syn-11
5.54
9.45
2.23
0.98
0.98
3.12
3.27
0.94
1.03
1.00
3.17
3.15
3.05
7.5 7.0 6.5 6.0 5.5 5.0 4.5 4.0 3.5 3.0 2.5 2.0 1.5 1.0 0.5 0.0
f1 (ppm)


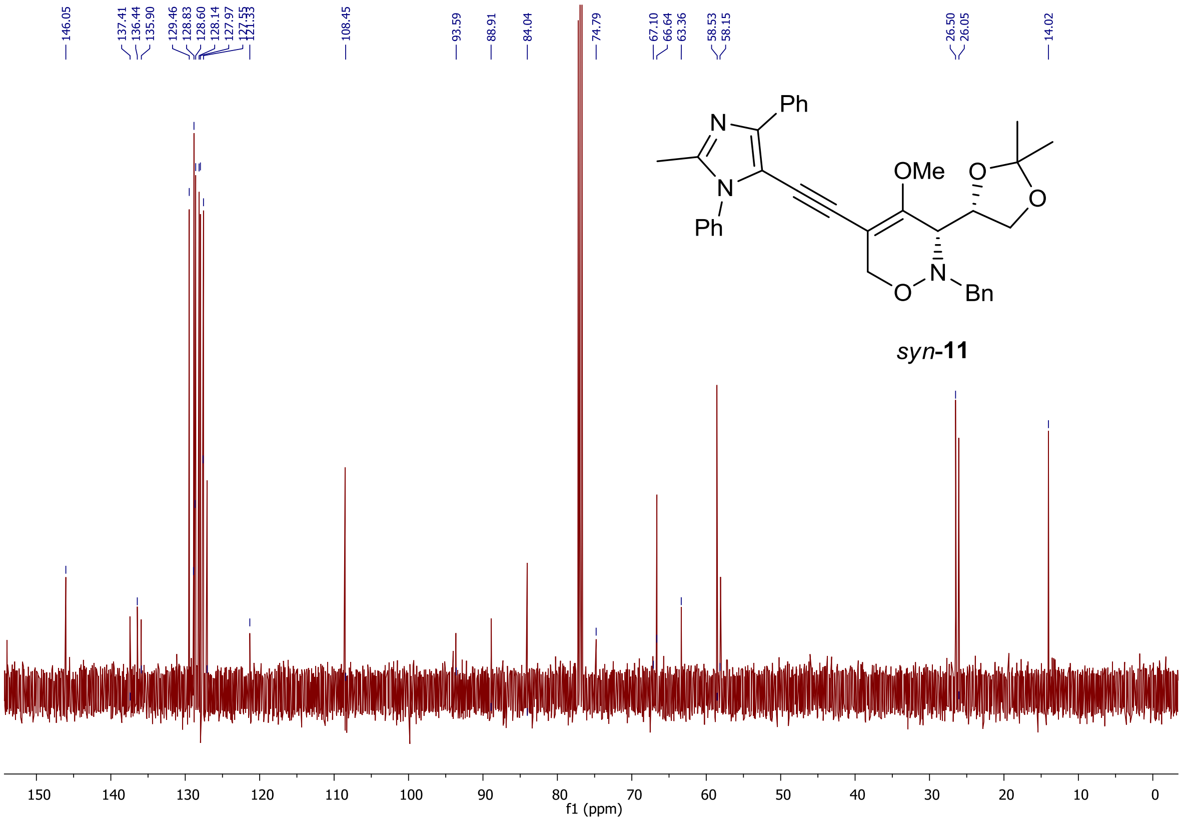

146.05
137.41
136.44
135.90
129.46
128.83
128.60
128.14
127.97
127.55
121.33
108.45
93.59
88.91
84.04
74.79
67.10
66.64
63.36
58.53
58.15
26.50
26.05
14.02
Ph
N
N
Ph
OMe
O
O
O
N
Bn
syn-11
150 140 130 120 110 100 90 80 70 60 50 40 30 20 10 0
f1 (ppm)

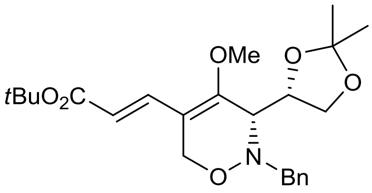
OMe
tBuO2C
O
O
N
O
Bn

*syn*-**14**

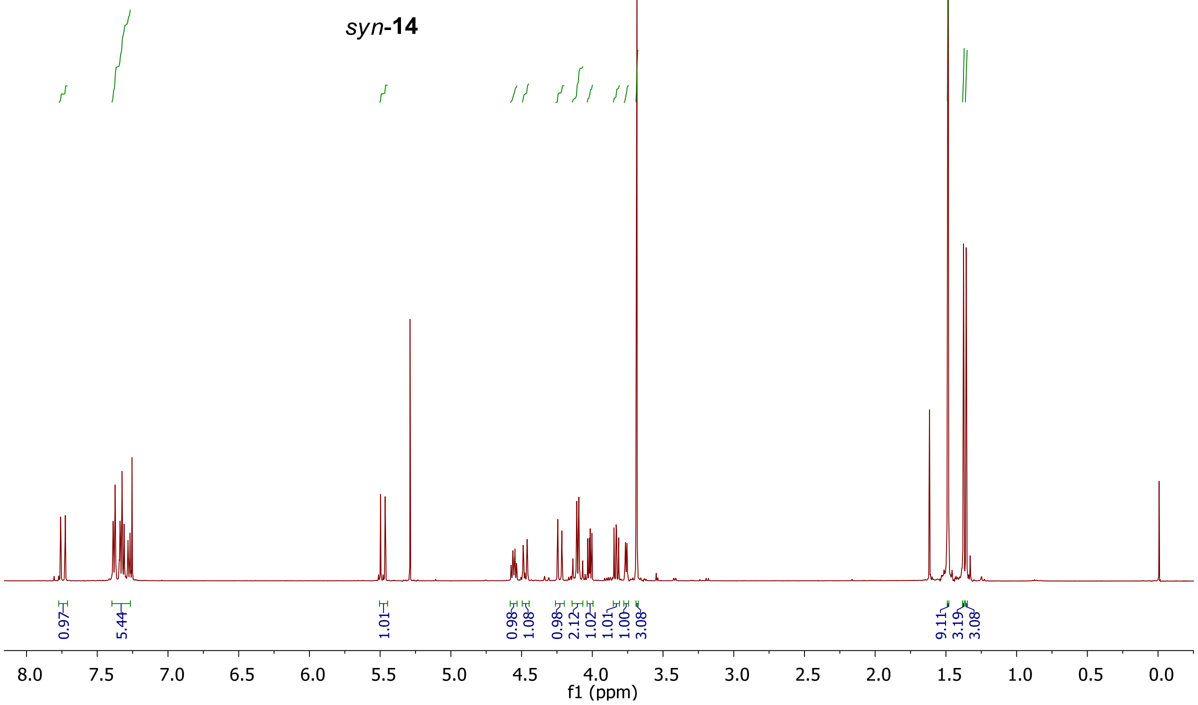
0.97
5.44
1.01
0.98
1.08
0.98
2.12
1.02
1.01
1.00
3.08
9.11
3.19
3.08
8.0 7.5 7.0 6.5 6.0 5.5 5.0 4.5 4.0 3.5 3.0 2.5 2.0 1.5 1.0 0.5 0.0
f1 (ppm)

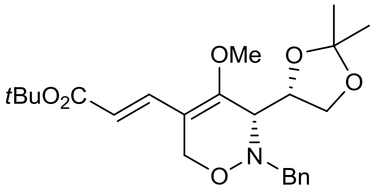
OMe
tBuO2C
O
O
N
O
Bn

*syn*-**14**

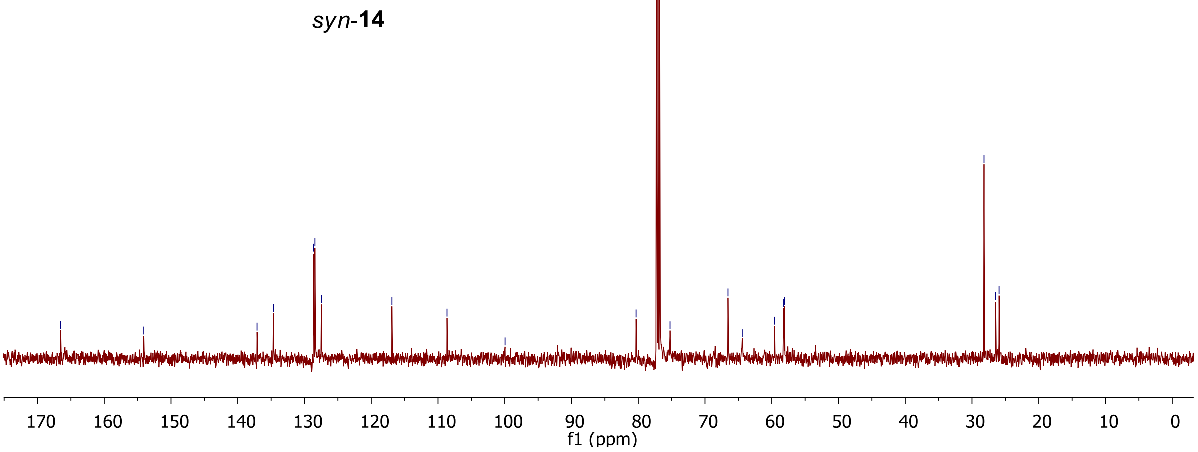
166.53
154.08
137.11
134.66
128.62
128.44
127.48
116.91
108.65
99.95
80.32
75.24
66.55
64.42
59.56
58.21
58.07
28.20
26.45
25.94
170 160 150 140 130 120 110 100 90 80 70 60 50 40 30 20 10 0
f1 (ppm)

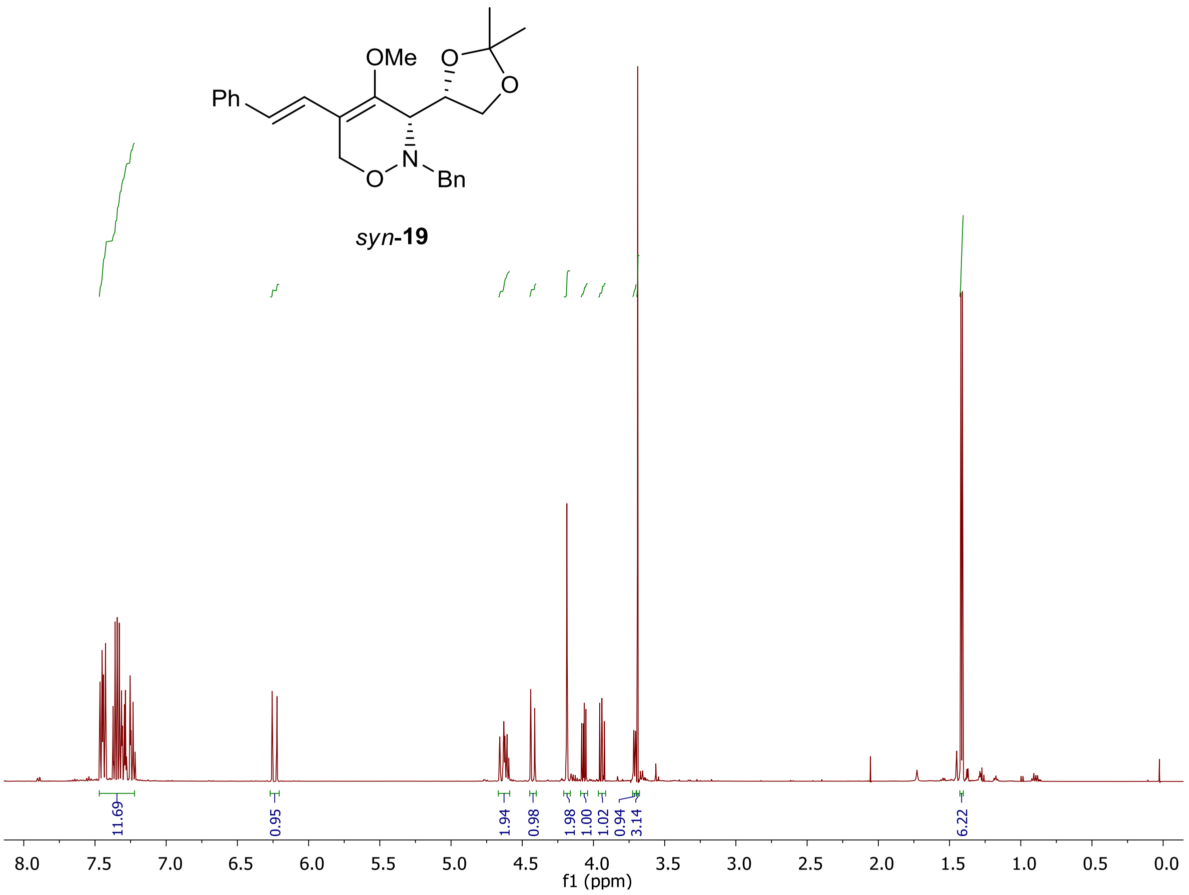
OMe
Ph
O
N
Bn
syn-19
11.69
0.95
1.94
0.98
1.98
1.00
1.02
0.94
3.14
6.22
8.0 7.5 7.0 6.5 6.0 5.5 5.0 4.5 4.0 3.5 3.0 2.5 2.0 1.5 1.0 0.5 0.0
f1 (ppm)

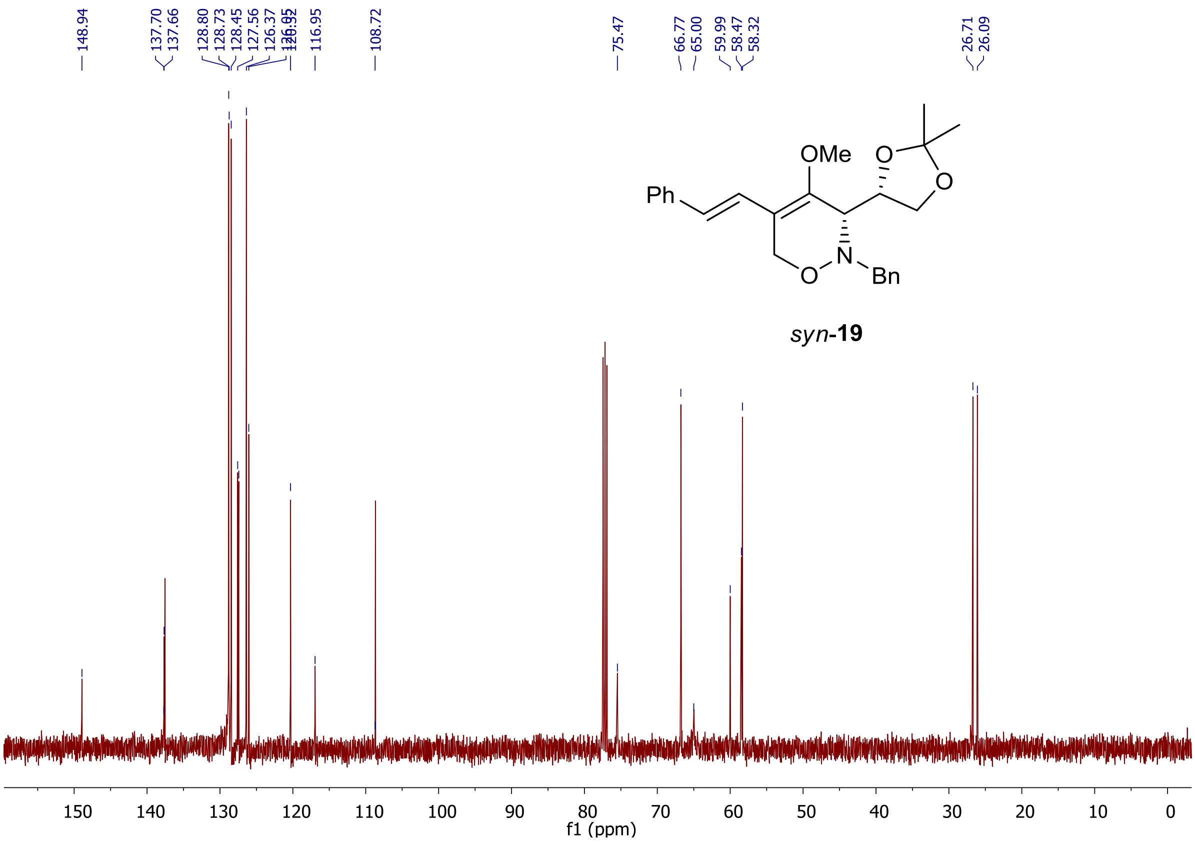
148.94
137.70
137.66
128.80
128.73
128.45
127.56
126.37
116.95
108.72
75.47
66.77
65.00
59.99
58.47
58.32
26.71
26.09
OMe
Ph
O
N
Bn
syn-19
150 140 130 120 110 100 90 80 70 60 50 40 30 20 10 0
f1 (ppm)

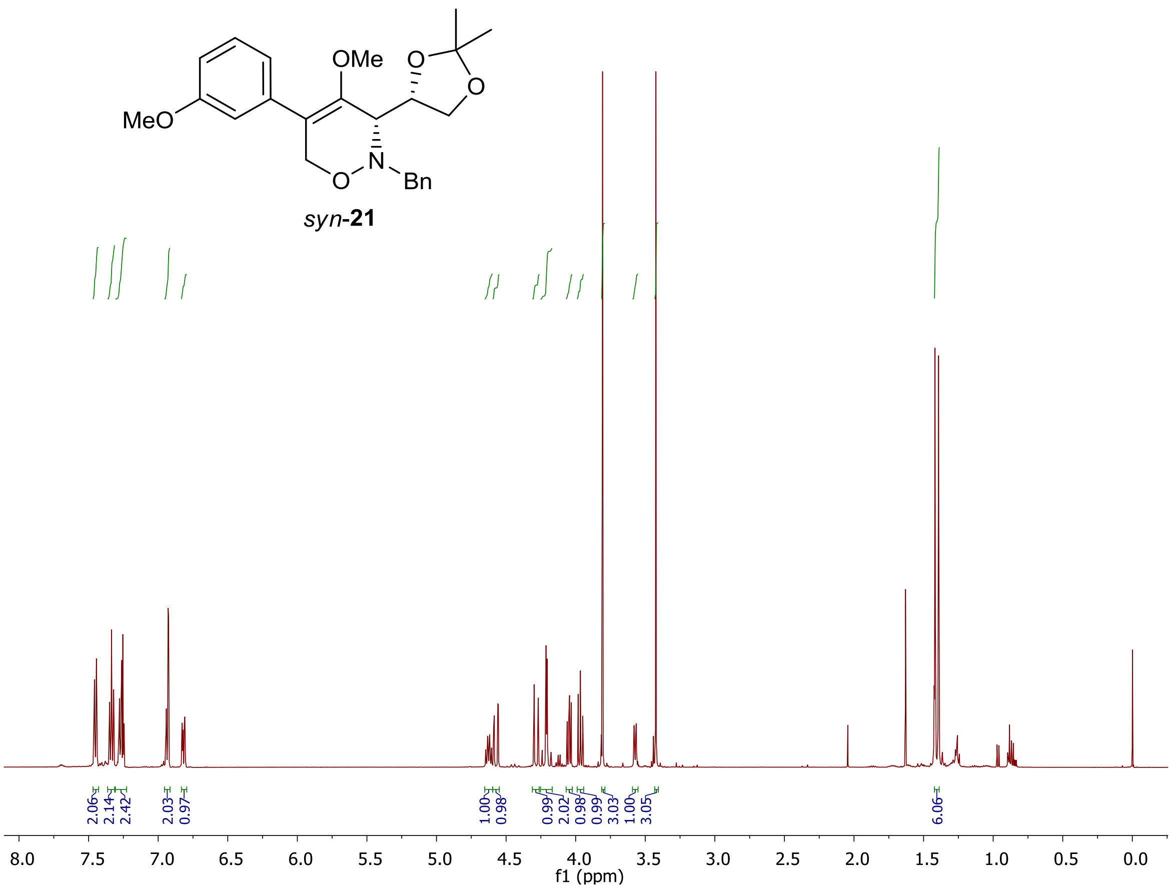
OMe
MeO
N
O
Bn
syn-21
2.06
2.14
2.42
2.03
0.97
1.00
0.98
0.99
2.02
0.98
0.99
3.03
1.00
3.05
6.06
8.0 7.5 7.0 6.5 6.0 5.5 5.0 4.5 4.0 3.5 3.0 2.5 2.0 1.5 1.0 0.5 0.0
f1 (ppm)

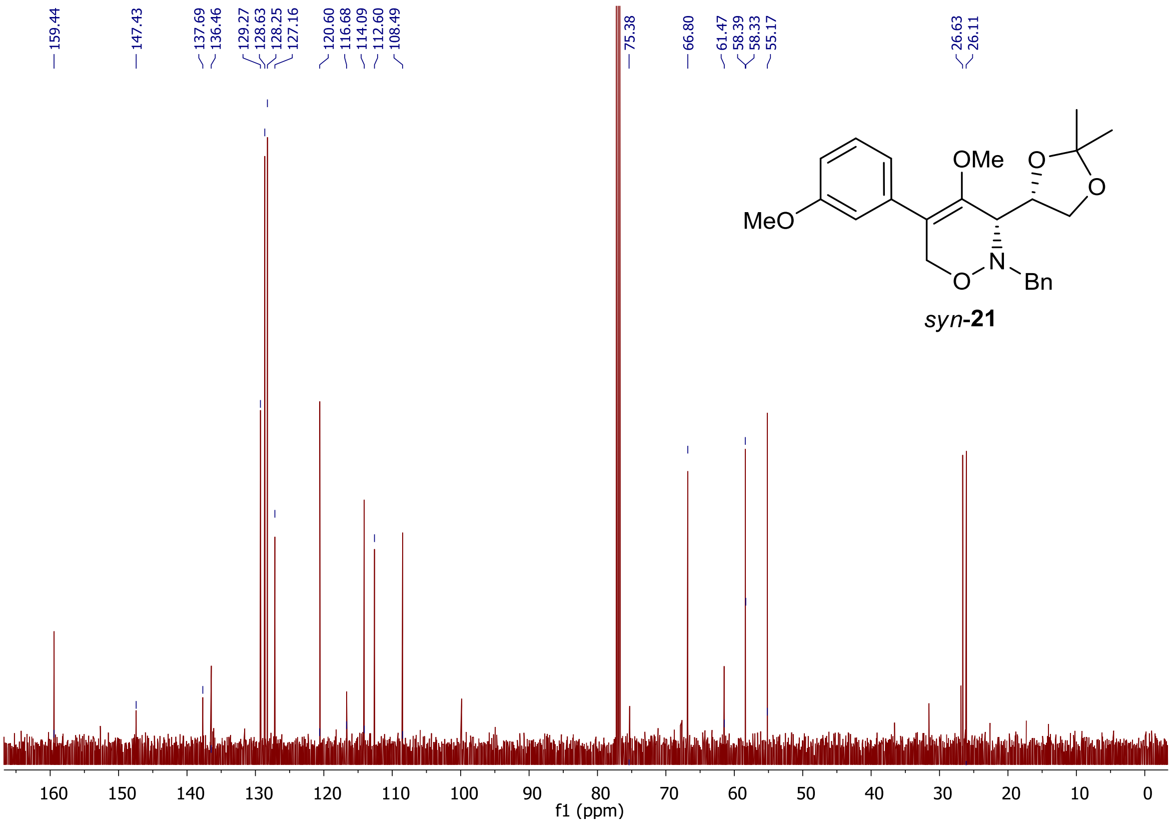
159.44
147.43
137.69
136.46
129.27
128.63
128.25
127.16
120.60
116.68
114.09
112.60
108.49
75.38
66.80
61.47
58.39
58.33
55.17
26.63
26.11
OMe
MeO
N
O
Bn
syn-21
160 150 140 130 120 110 100 90 80 70 60 50 40 30 20 10 0
f1 (ppm)

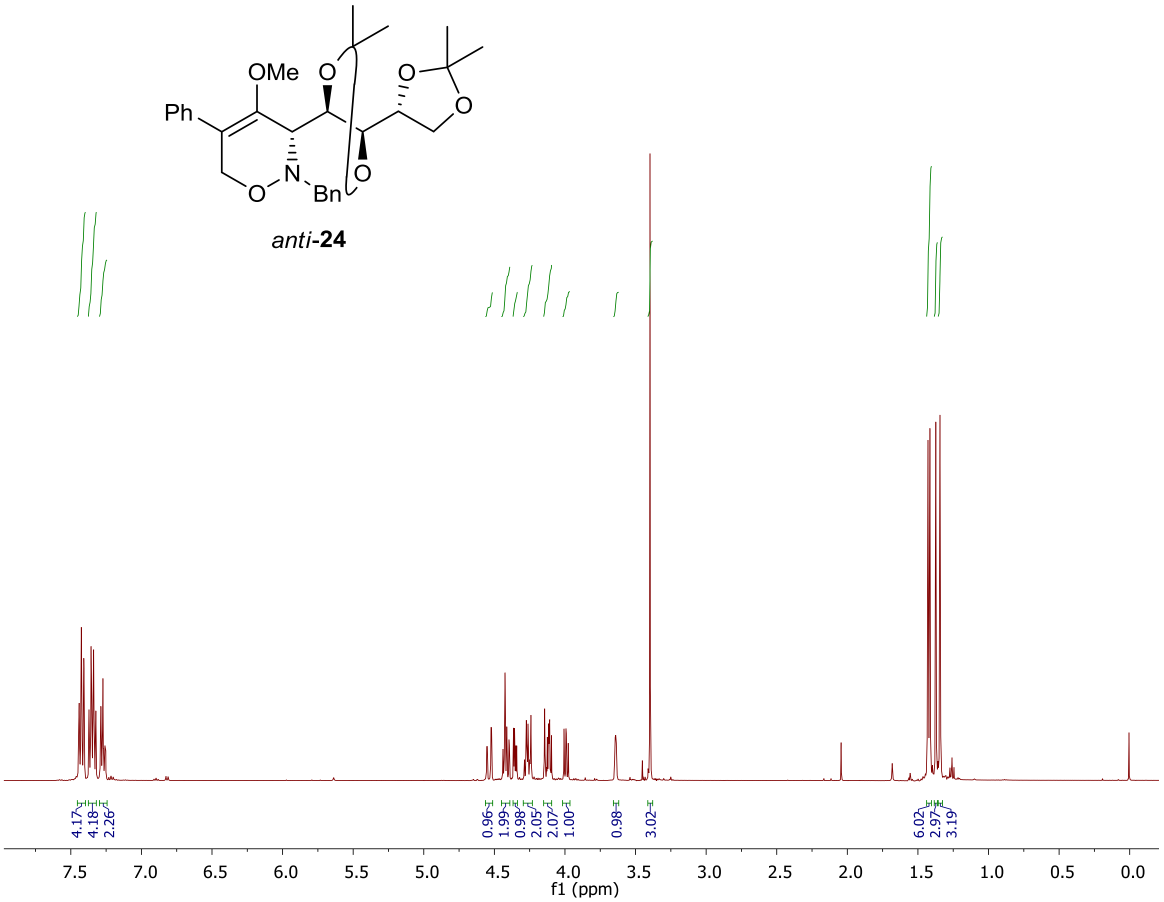

OMe
Ph
N
Bn
O
anti-24
4.17
4.18
2.26
0.96
1.99
0.98
2.05
2.07
1.00
0.98
3.02
6.02
2.97
3.19
7.5
7.0
6.5
6.0
5.5
5.0
4.5
4.0
3.5
3.0
2.5
2.0
1.5
1.0
0.5
0.0
f1 (ppm)


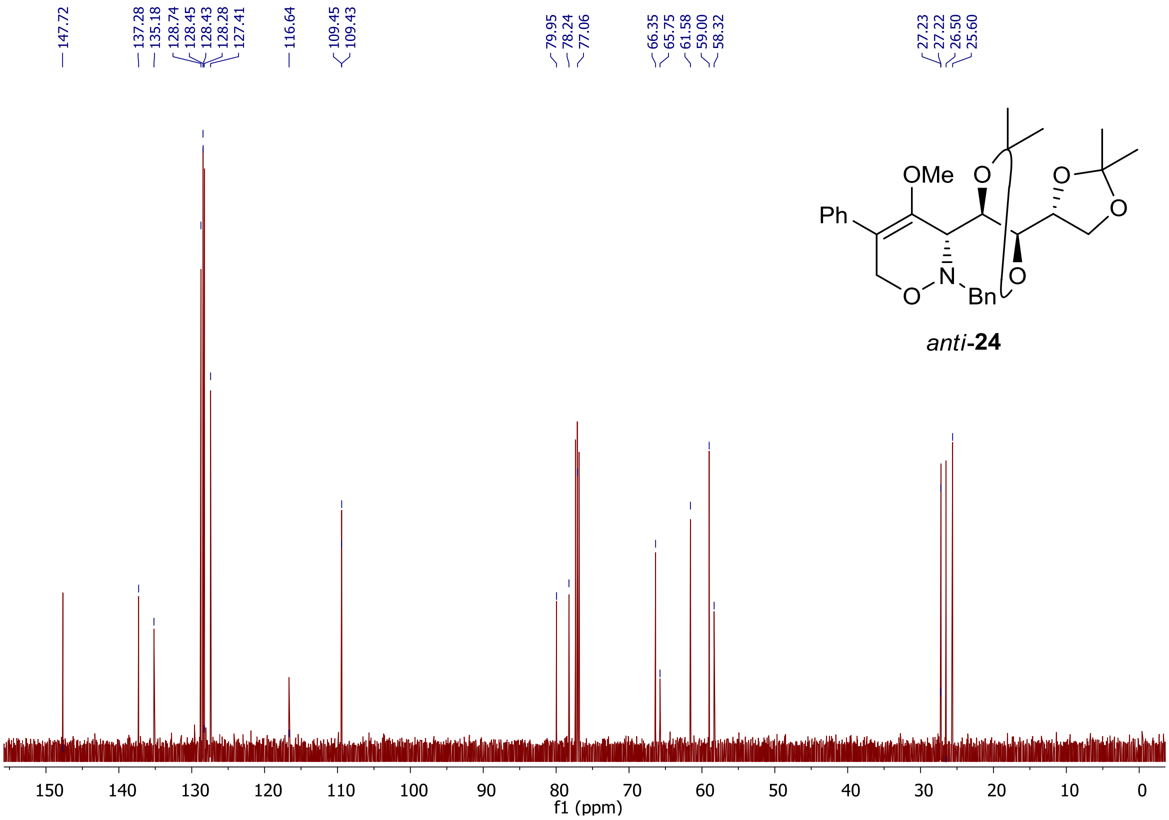

147.72
137.28
135.18
128.74
128.45
128.43
128.28
127.41
116.64
109.45
109.43
79.95
78.24
77.06
66.35
65.75
61.58
59.00
58.32
27.23
27.22
26.50
25.60
OMe
Ph
N
Bn
O
anti-24
150
140
130
120
110
100
90
80
70
60
50
40
30
20
10
0
f1 (ppm)

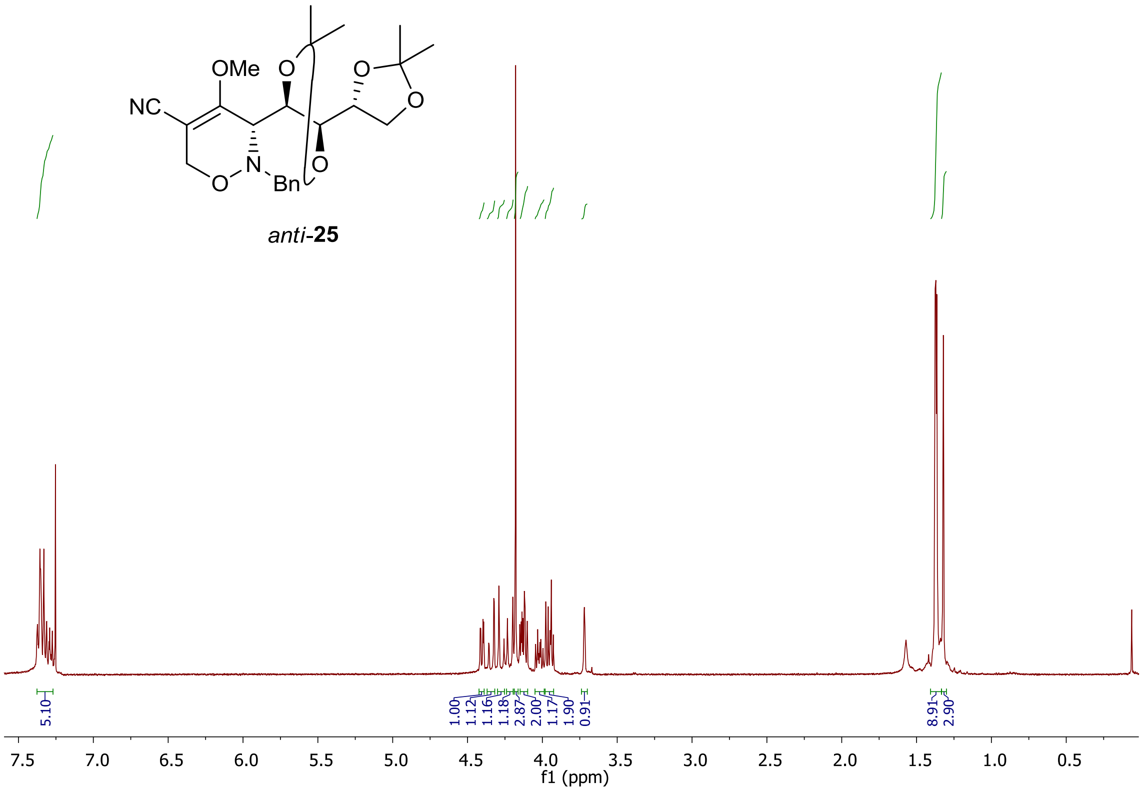
OMe
NC
O
O
O
O
O
N
Bn
anti-25
5.10
1.00
1.12
1.16
1.18
2.87
2.00
1.17
1.90
0.91
8.91
2.90
7.5 7.0 6.5 6.0 5.5 5.0 4.5 4.0 3.5 3.0 2.5 2.0 1.5 1.0 0.5
f1 (ppm)

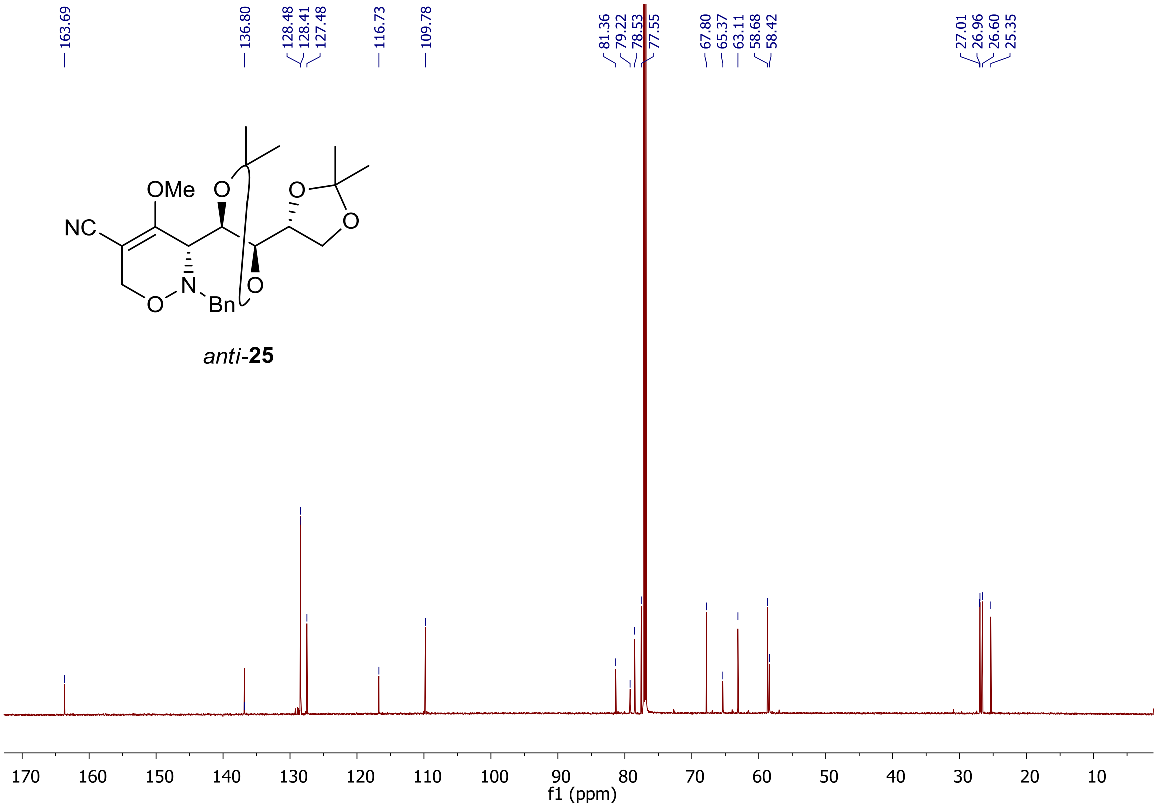
163.69
136.80
128.48
128.41
127.48
116.73
109.78
81.36
79.22
78.53
77.55
67.80
65.37
63.11
58.68
58.42
27.01
26.96
26.60
25.35
OMe
NC
O
O
O
O
O
N
Bn
anti-25
170 160 150 140 130 120 110 100 90 80 70 60 50 40 30 20 10
f1 (ppm)

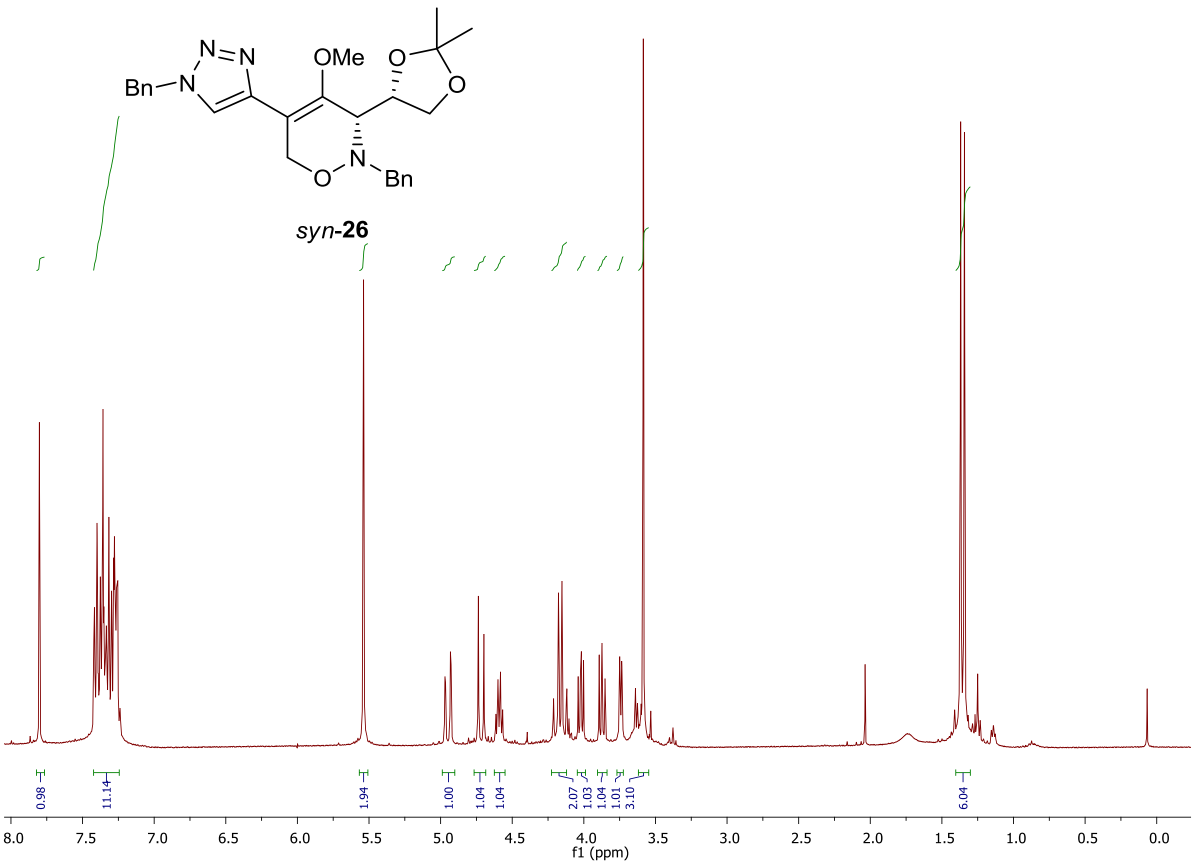
Bn–N N=N OMe O O
N Bn
syn-26
0.98 11.14 1.94 1.00 1.04 1.04 2.07 1.03 1.04 1.01 3.10 6.04
8.0 7.5 7.0 6.5 6.0 5.5 5.0 4.5 4.0 3.5 3.0 2.5 2.0 1.5 1.0 0.5 0.0
f1 (ppm)

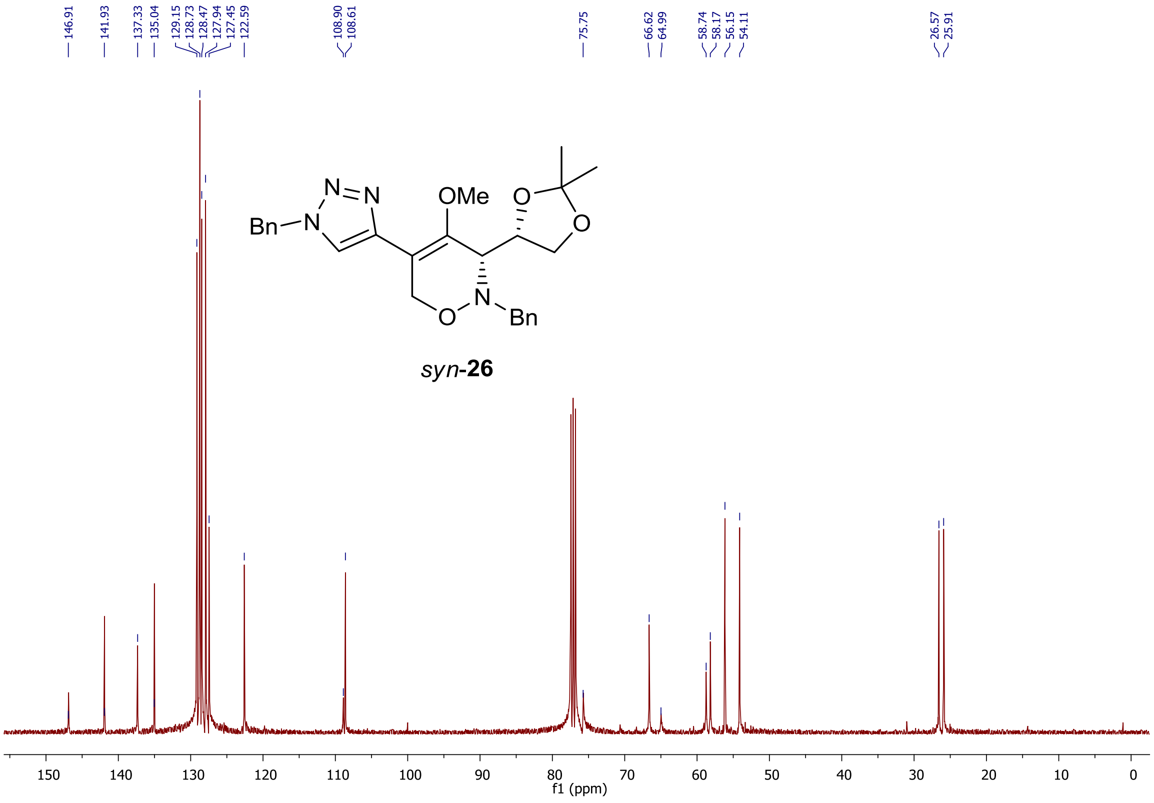
146.91
141.93
137.33
135.04
129.15
128.73
128.47
127.94
127.45
122.59
108.90
108.61
75.75
66.62
64.99
58.74
58.17
56.15
54.11
26.57
25.91
Bn–N N=N OMe O O
N Bn
syn-26
150 140 130 120 110 100 90 80 70 60 50 40 30 20 10 0
f1 (ppm)

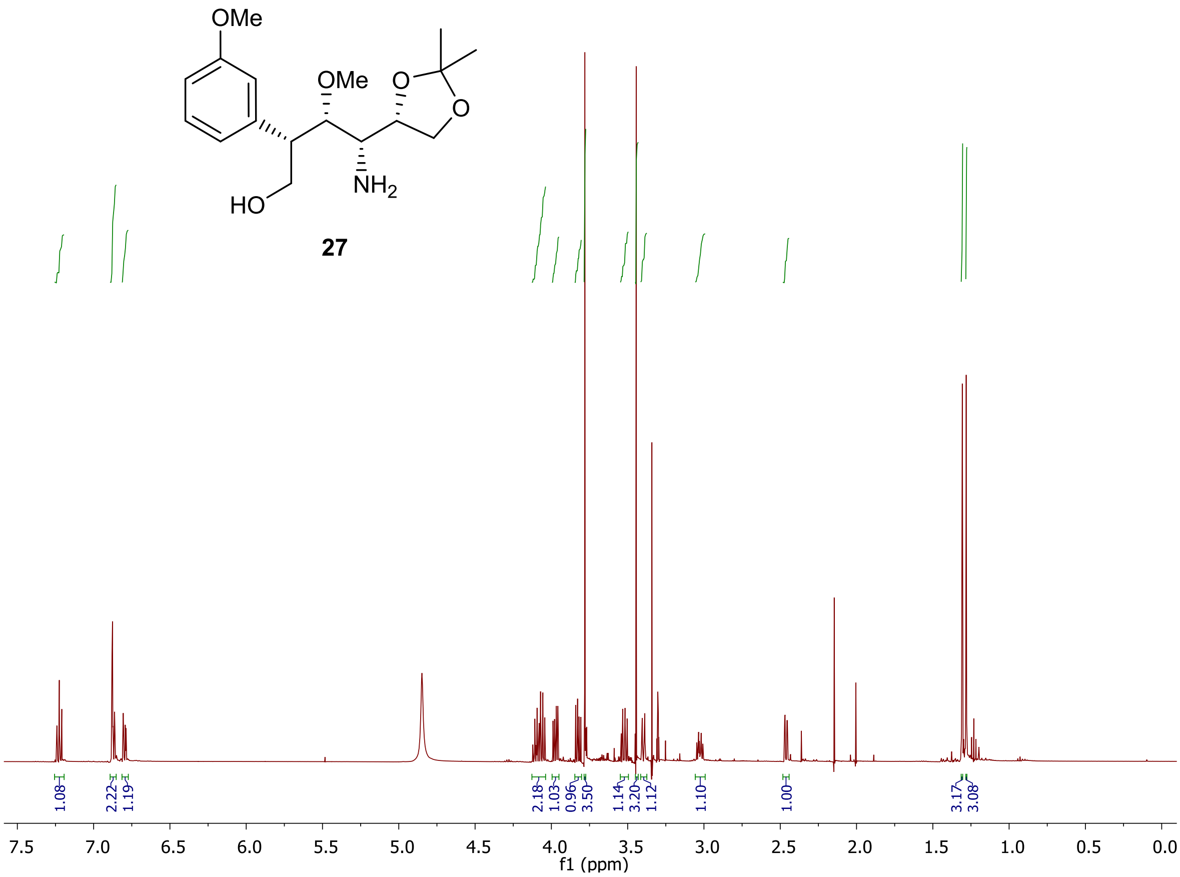
OMe
OMe
O
O
NH2
HO
27
1.08
2.22
1.19
2.18
1.03
0.96
3.50
1.14
3.20
1.12
1.10
1.00
3.17
3.08
7.5 7.0 6.5 6.0 5.5 5.0 4.5 4.0 3.5 3.0 2.5 2.0 1.5 1.0 0.5 0.0
f1 (ppm)

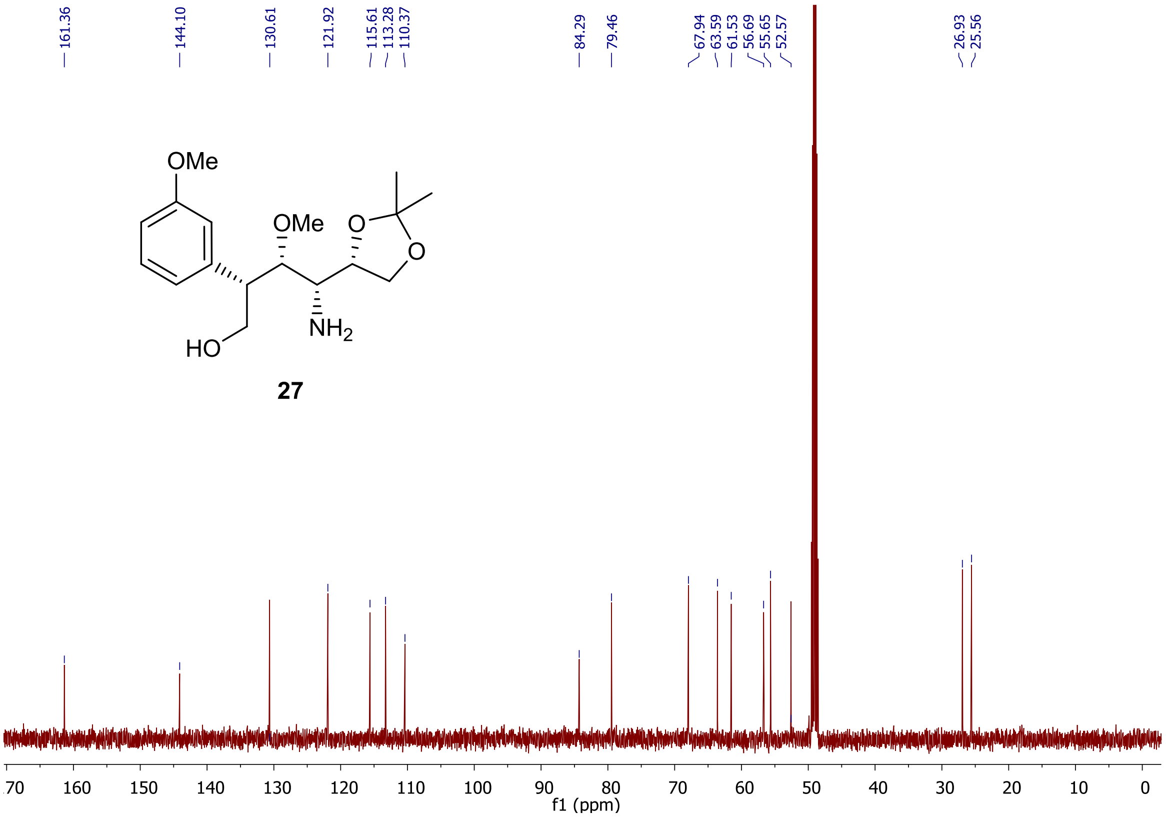
161.36
144.10
130.61
121.92
115.61
113.28
110.37
84.29
79.46
67.94
63.59
61.53
56.69
55.65
52.57
26.93
25.56
OMe
OMe
O
O
NH2
HO
27
70 160 150 140 130 120 110 100 90 80 70 60 50 40 30 20 10 0
f1 (ppm)

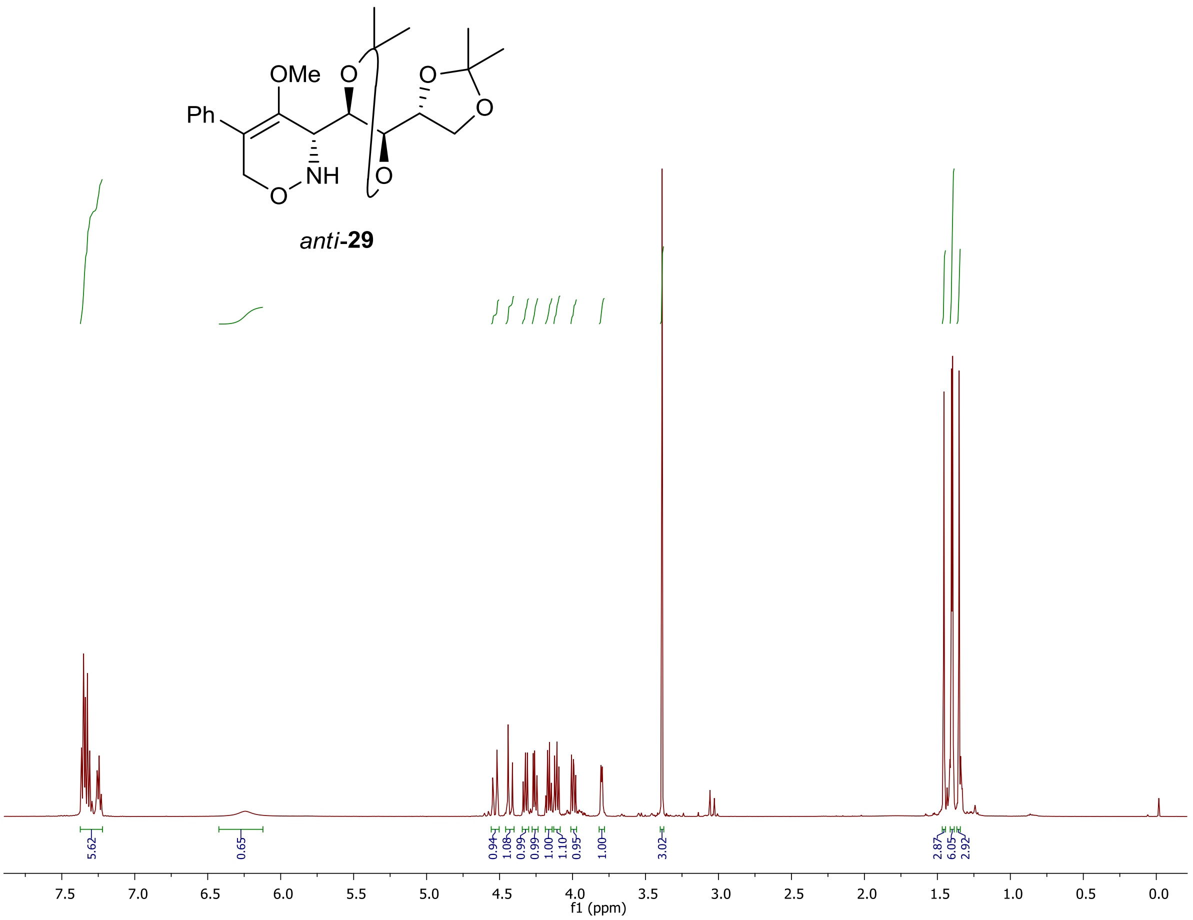
OMe
Ph
NH
O
anti-29
5.62
0.65
0.94
1.08
0.99
0.99
1.00
1.10
0.95
1.00
3.02
2.87
6.05
2.92
f1 (ppm)

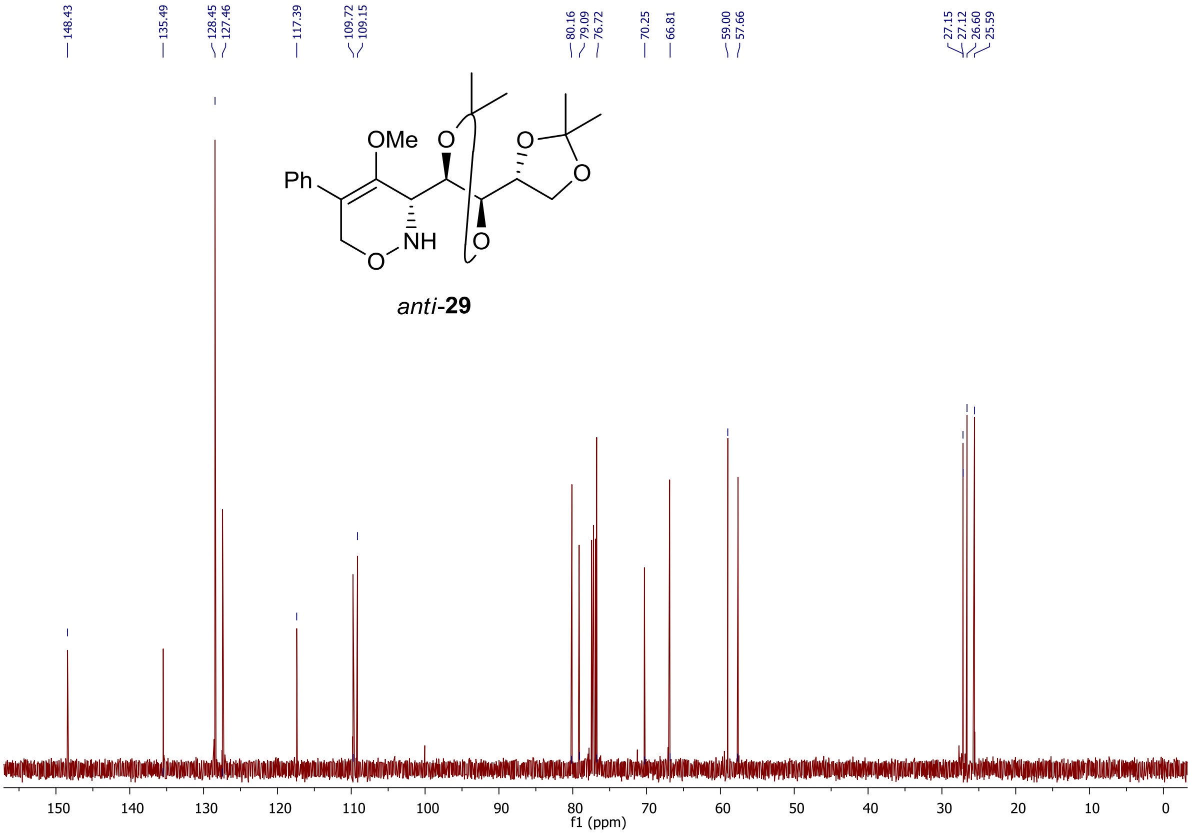
148.43
135.49
128.45
127.46
117.39
109.72
109.15
80.16
79.09
76.72
70.25
66.81
59.00
57.66
27.15
27.12
26.60
25.59
OMe
Ph
NH
O
anti-29
f1 (ppm)

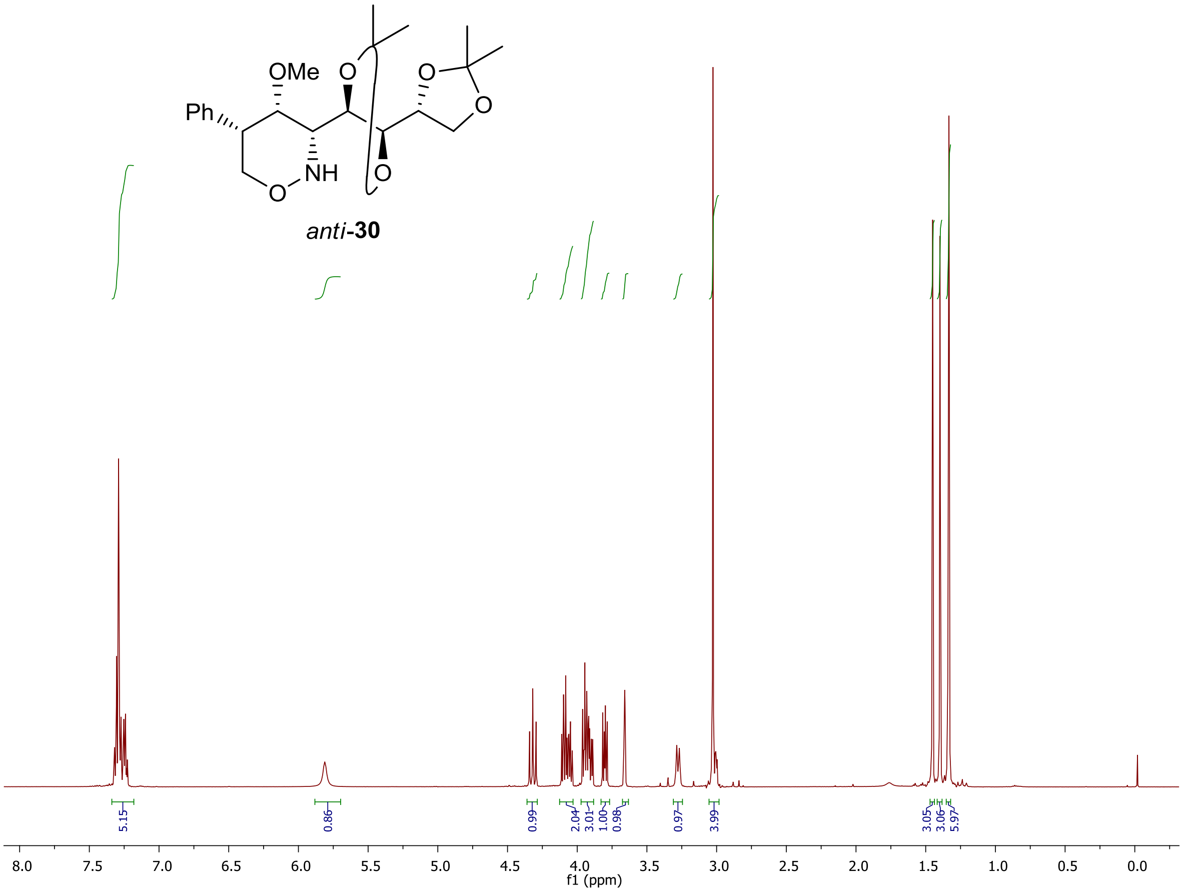
OMe
Ph
NH
O
anti-30
5.15
0.86
0.99
2.04
3.01
1.00
0.98
0.97
3.99
3.05
3.06
5.97
f1 (ppm)

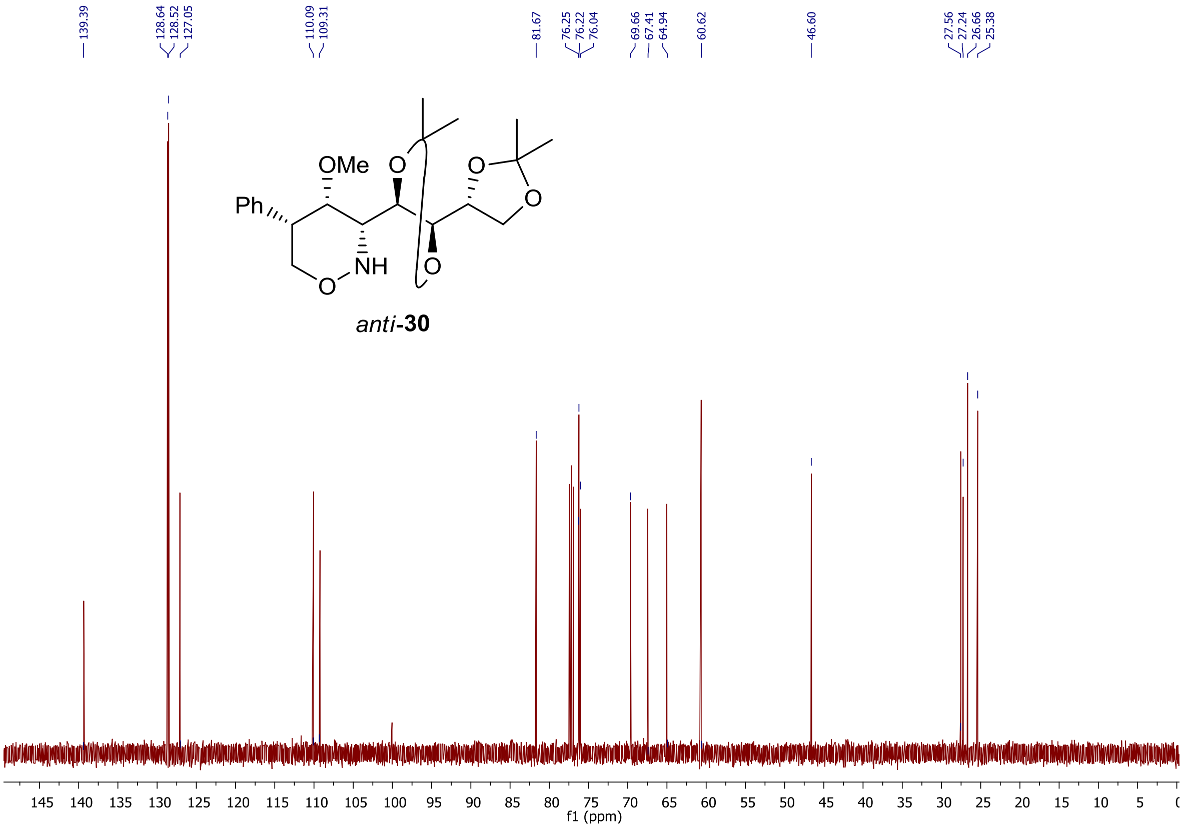
139.39
128.64
128.52
127.05
110.09
109.31
81.67
76.25
76.22
76.04
69.66
67.41
64.94
60.62
46.60
27.56
27.24
26.66
25.38
OMe
Ph
NH
O
anti-30
f1 (ppm)
